# Supplementary material for: Drug repurposing for aging research using model organisms
Source: Aging Cell. 2017 Jun 16;16(5):1006–15. doi: 10.1111/acel.12626 (PMC5595691; doi:10.1111/acel.12626)
Supplement: Supplementary file 7 — Data S1 Zip‐Archive of all report cards. [file ACEL-16-1006-s007.zip › RC_EDO.pdf]

EDO

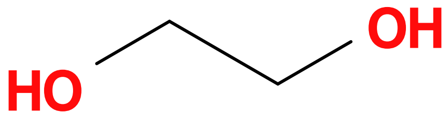

Database identifiers

ChEMBLCompound	CHEMBL457299
CHEBI	30742
ZINC	ZINC05224354
eMolecules	475071

Ranking

	Rank	Score
Drosophila	529/697	0.223
C. elegans	576/591	0.0

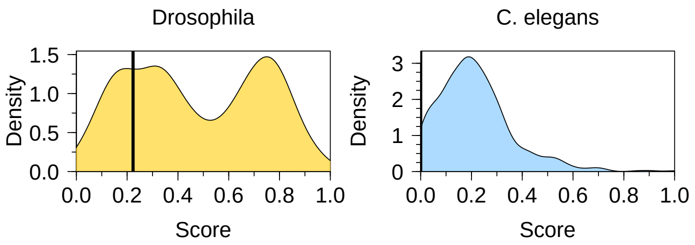

	Ageing implication	Domain conservation	Binding site conservation	Binding affinity	Bioavailability	Lipinski	Promiscuity	Purchasability	Drug approval	**Total**
Drosophila	1.0	0.982	1.0	0.366	(0.9)	0.0	-0.2	0.1	0.0	0.223
C. elegans	1.0	0.982	1.0	0.366	0.244	0.0	-0.2	0.1	0.0	0.0

Names

- **ethylene glycol**
- 1,2-Dihydroxyethane
- 2-Hydroxyethanol
- Ethanediol
- Glycol
- Ho-ch2-ch2-oh
- Monoethylene glycol

Roles

toxin, solvent, metabolite

Status

Approved drug (according to ChEMBL)	No
Number of Rule of 5 violations	0
Binding affinity to original target in log units (RF-Score prediction)	4.45
Burns *C. elegans* bioavailability prediction	-2.38

Compound Target Characteristics

Serine/threonine-protein kinase Chk1

Best gene implication in ageing for this target family came from gene O14757 annotated in UniProt release 2014_02. Annotation GO subterm of 7568 (aging) was Non-traceable Author Statement

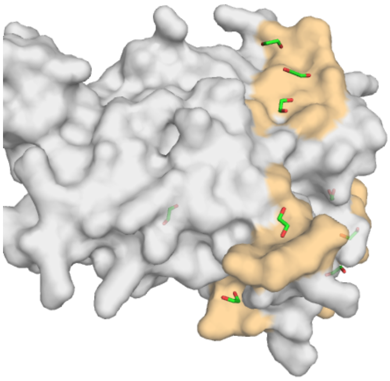

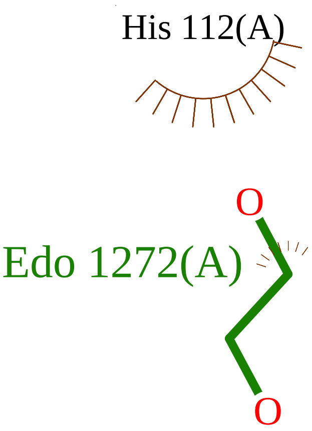

protein	amino acids contacts (binding site)
PDB:2xez:chainA:O14757	R E V K C Y R R E F D E D Q H A G I G R K P E T V R Y N N E L N K T Y R E F H
PDB:2xf0:chainA:O14757	R E V K C Y R R E F D E D Q H A G I G R K P E T V R Y N N E L N K T Y R E F H
PDB:2yer:chainA:O14757	R E V K C Y R R E F D E D Q H A G I G R K P E T V R Y N N E L N K T Y R E F H
PDB:2ym3:chainA:O14757	R E V K C Y R R E F D E D Q H A G I G R K P E T V R Y N N E L N K T Y R E F H
PDB:2ym4:chainA:O14757	R E V K C Y - - E F D E D Q H A G I G R K P E T V R Y N N E L N K T Y R E F H
PDB:2ym5:chainA:O14757	R E V K C Y R R E F D E D Q H A G I G R K P E T V R Y N N E L N K T Y R E F H
PDB:2ym6:chainA:O14757	R E V K C Y R R E F D E D Q H A G I G R K P E T V R Y N N E L N K T Y R E F H
PDB:2ym7:chainA:O14757	R E V K C Y R R E F D E D Q H A G I G R K P E T V R Y N N E L N K T Y R E F H
PDB:2ym8:chainA:O14757	R E V K C Y R R E F D E D Q H A G I G R K P E T V R Y N N E L N K T Y R E F H
tr:J3KN87:J3KN87_HUMAN	R E V K C Y R R E F D E D Q H A G I G R K P E T V R Y N N E L N K T Y R E F H
sp:O14757:CHK1_HUMAN	R E V K C Y R R E F D E D Q H A G I G R K P E T V R Y N N E L N K T Y R E F H
sp:Q91ZN7:CHK1_RAT	R E V K C Y R R E F D E D Q H A G I G R K P E T V R H N N E L N K T Y R E F H
tr:G3UYB1:G3UYB1_MOUSE	R E V K C Y R R E F D E D Q H A G I G R K P E T V R H N N E L N K T Y R E F H
tr:G3UYC5:G3UYC5_MOUSE	R E V K C Y R R E F D E D Q H A G I G R K P E T V R H N N E L N K T Y R E F H
sp:O35280:CHK1_MOUSE	R E V K C Y R R E F D E D Q H A G I G R K P E T V R H N N E L N K T Y R E F H
sp:O61661:CHK1_DROME	R E V K C F R S E F D E D Q T S Q R G R K P E T M R C K G E L D K T Y Q A Y H
sp:Q9N3Z3:CHK1_CAEEL	T E A K L I R N E F D E D Q K C D N D R K P E T L R N K G E L D L T Y G K Y R
sp:P38147:CHK1_YEAST	Q S L K V I N V D F D E D Q Q S E C G R K P E S Q R R K G L S M D S Y E G Y Y

protein	whole protein ident	simil	domain-based ident	simil	contact-based ident	simil
PDB:2xez:chainA:O14757	1.0	1.0	1.0	1.0	1.0	1.0
PDB:2xf0:chainA:O14757	1.0	1.0	1.0	1.0	1.0	1.0
PDB:2yer:chainA:O14757	1.0	1.0	1.0	1.0	1.0	1.0
PDB:2ym3:chainA:O14757	1.0	1.0	1.0	1.0	1.0	1.0
PDB:2ym4:chainA:O14757	1.0	1.0	1.0	1.0	0.97	0.94
PDB:2ym5:chainA:O14757	1.0	1.0	1.0	1.0	1.0	1.0
PDB:2ym6:chainA:O14757	1.0	1.0	1.0	1.0	1.0	1.0
PDB:2ym7:chainA:O14757	1.0	1.0	1.0	1.0	1.0	1.0
PDB:2ym8:chainA:O14757	1.0	1.0	1.0	1.0	1.0	1.0
tr:J3KN87:J3KN87_HUMAN	0.91	0.91	1.0	1.0	1.0	1.0
sp:O14757:CHK1_HUMAN	1.0	1.0	1.0	1.0	1.0	1.0
sp:Q91ZN7:CHK1_RAT	0.94	0.98	0.96	0.99	0.98	0.99
tr:G3UYB1:G3UYB1_MOUSE	0.84	0.89	0.95	0.99	0.97	0.98
tr:G3UYC5:G3UYC5_MOUSE	0.87	0.91	0.95	0.99	0.97	0.98
sp:O35280:CHK1_MOUSE	0.93	0.98	0.95	0.99	0.97	0.98
sp:O61661:CHK1_DROME	0.44	0.75	0.5	0.8	0.55	0.7
sp:Q9N3Z3:CHK1_CAEEL	0.31	0.62	0.41	0.7	0.45	0.61
sp:P38147:CHK1_YEAST	0.23	0.62	0.3	0.68	0.29	0.53

grp (FBgn0261278) associated phenotypes

cell cycle defective, chemical sensitive, decreased cell death, maternal effect, mitotic cell cycle defective, non-rescuable maternal effect, partially lethal - majority die, radiation resistant, radiation sensitive

(Information from FlyBase)

grp (UniProt:O61661) annotation

Function: Serine/threonine-protein kinase which is required for checkpoint-mediated cell cycle arrest and activation of DNA repair in response to the presence of DNA damage or unreplicated DNA. May also negatively regulate cell cycle progression during unperturbed cell cycles. May phosphorylate the CDC25 phosphatase stg, which promotes its degradation. This results in increased inhibitory tyrosine phosphorylation of CDC2-cyclin complexes and consequent inhibition of cell cycle progression. (PubMed:10209095, PubMed:10469601, PubMed:10980701, PubMed:12919679, PubMed:14711410, PubMed:15723794, PubMed:15860729, PubMed:16079276, PubMed:7925016, PubMed:9197245, PubMed:9214509).
Subcellular location: Nucleus ECO:0000269—PubMed:10980701, PubMed:16079276).
Developmental stage: Expressed both maternally and zygotically. Maternally supplied mRNA is degraded during progression from nuclear stage 12 to nuclear stage 13. Zygotic expression is seen at reduced levels later in embryogenesis and during larval development. Higher expression is seen in pupae, coincident with ovarian differentiation. May be activated during the syncytial blastoderm divisions which precede cellularization, the Drosophila equivalent of the midblastula transition (MBT). Developmentally regulated activation of the DNA replication checkpoint may occur as the nucleo-cytoplasmic ratio increases and maternal replication factors are depleted. Elongation of the embryonic cell cycle may allow time for the transcription of genes that initiate the switch from maternal to zygotic control of embryogenesis. (PubMed:9197245, PubMed:9214509).
Ptm: Phosphorylated in a MEI-41/ATR dependent manner in response to DNA damage or the presence of unreplicated DNA. (PubMed:15860729).

(Information from UniProt)

chk-1 (WBGene00000498) associated phenotypes

lethal, locomotion variant, mid larval lethal, sterile

(Information from WormBase)

chk-1 (UniProt:Q9N3Z3) annotation

Function: Serine/threonine-protein kinase which is required for checkpoint-mediated cell cycle arrest and activation of DNA repair in response to the presence of DNA damage or unreplicated

DNA. May also negatively regulate cell cycle progression during unperturbed cell cycles. Required for checkpoint mediated cell cycle arrest in response to DNA damage in germline cells. Essential for embryogenesis. (PubMed:15326393).
Subcellular location: Cytoplasm Nucleus
Tissue specificity: Expressed in the germline. (PubMed:15326393).
Developmental stage: Highly expressed in the embryo. (PubMed:15326393).

(Information from UniProt)

Serine protease HTRA2, mitochondrial

Best gene implication in ageing for this target family came from gene F6TCV0 via mapping the annotation from Ensembl ENSMUSG00000068329 via mapping the annotation from EntrezGene 64704 via mapping the annotation from GenAgeModels 1802 annotated in GenAge release 17.

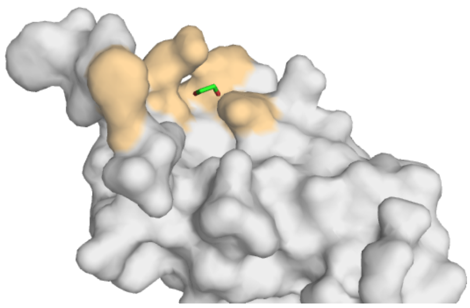

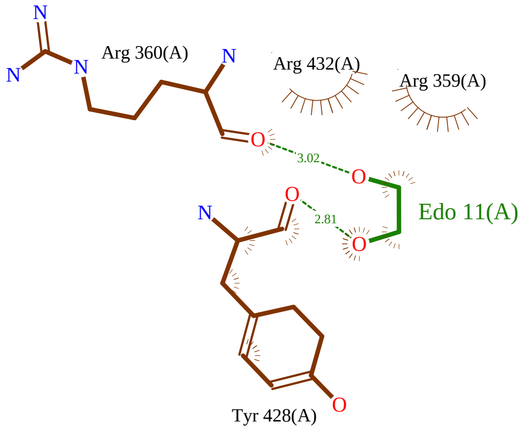

protein	amino acids contacts (binding site)
PDB:2pzd:chainA:O43464	R R Y R F
sp:O43464:HTRA2_HUMAN	R R Y R -
tr:B0BNB9:B0BNB9_RAT	R R Y R -
tr:F6TCV0:F6TCV0_MOUSE	R R Y R -
tr:D3YX28:D3YX28_MOUSE	R R Y R -
tr:D3YX27:D3YX27_MOUSE	R R Y R -
tr:Q3TXN0:Q3TXN0_MOUSE	R R Y R -
sp:Q9JIY5:HTRA2_MOUSE	R R Y R -
sp:Q9VFJ3:HTRA2_DROME	K R Y A -

	whole protein		domain-based		contact-based	
protein	ident	simil	ident	simil	ident	simil
PDB:2pzd:chainA:O43464	0.93	0.94	0.93	0.94	1.0	1.0
sp:O43464:HTRA2_HUMAN	1.0	1.0	1.0	1.0	0.8	0.5
tr:B0BNB9:B0BNB9_RAT	0.87	0.95	0.94	0.98	0.8	0.5
tr:F6TCV0:F6TCV0_MOUSE	0.6	0.63	0.91	0.96	0.8	0.5
tr:D3YX28:D3YX28_MOUSE	0.64	0.73	0.62	0.67	0.8	0.5
tr:D3YX27:D3YX27_MOUSE	0.78	0.87	0.62	0.67	0.8	0.5
tr:Q3TXN0:Q3TXN0_MOUSE	0.83	0.92	0.93	0.98	0.8	0.5
sp:Q9JIY5:HTRA2_MOUSE	0.85	0.94	0.93	0.98	0.8	0.5
sp:Q9VFJ3:HTRA2_DROME	0.39	0.71	0.44	0.81	0.4	0.24

HtrA2 (FBgn0038233) associated phenotypes

chemical resistant, chemical sensitive, decreased cell death, dominant, flightless, heat sensitive, locomotor behavior defective, male fertile, partially lethal - majority die, radiation resistant, short lived

(Information from FlyBase)

HtrA2 (UniProt:Q9VFJ3) annotation

Function: Serine protease that shows proteolytic activity against a non-specific substrate beta-casein. Promotes or induces cell death either by direct binding to and inhibition of BIRC proteins (also called inhibitor of apoptosis proteins, IAPs), leading to an increase in caspase activity, or by a BIRC inhibition-independent, caspase-independent and serine protease activity-dependent mechanism. Can antagonize antiapoptotic activity of th by directly inducing the degradation of th. (PubMed:17397804, PubMed:17557079, PubMed:18259196).
Subunit: Interacts with th/DIAP1 (via BIR 2 domain). (PubMed:18259196).
Subcellular location: Mitochondrion intermembrane space (PubMed:17397804, PubMed:18259196); Single-pass membrane protein. Mitochondrion membrane ; Single-pass membrane protein Note=Predominantly present in the intermembrane space. Released into the cytosol following apoptotic stimuli, such as UV treatment. The extramitochondrial protein does not diffuse throughout the cytosol but stays near the mitochondria. (, PubMed:17397804, PubMed:18259196).
Developmental stage: Expressed during all stages of development; high in embryos, declined in larvae and pupae and high again in adults. (PubMed:17397804, PubMed:17557079).
Ptm: Ubiquitinated by th, thereby antagonizing induced cell death.
Ptm: Autoproteolytically cleaved into active forms in the mitochondria. (PubMed:17557079, PubMed:18259196).

(Information from UniProt)

Mitotic checkpoint serine/threonine-protein kinase BUB1 beta

Best gene implication in ageing for this target family came from gene Q9Z1S0 via mapping the annotation from Ensembl ENSMUSG00000040084 via mapping the annotation from EntrezGene 12236 via mapping the annotation from GenAgeModels 0050 annotated in GenAge release 17.

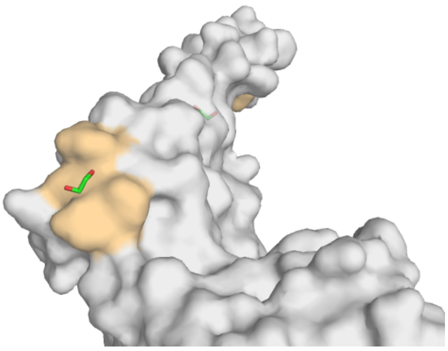

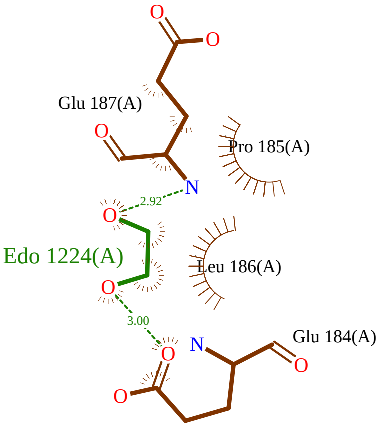

protein	amino acids contacts (binding site)
PDB:2wvi:chainA:O60566	E P L E E E E
sp:O60566:BUB1B_HUMAN	E P L E E E E
tr:F1LMI1:F1LMI1_RAT	E P L D N E E
sp:Q9Z1S0:BUB1B_MOUSE	E P L D N E E
tr:A1Z6I7:A1Z6I7_DROME	E P L E E N A
tr:Q7KN56:Q7KN56_DROME	E P L E E N A
tr:Q21776:Q21776_CAEEL	- - R D N S G
sp:P47074:MAD3_YEAST	R P N K L E I
sp:P41695:BUB1_YEAST	R P Y N N P D

protein	whole protein ident	simil	domain-based ident	simil	contact-based ident	simil
PDB:2wvi:chainA:O60566	1.0	1.0	1.0	1.0	1.0	1.0
sp:O60566:BUB1B_HUMAN	1.0	1.0	1.0	1.0	1.0	1.0
tr:F1LMI1:F1LMI1_RAT	0.76	0.91	0.88	0.97	0.71	0.87
sp:Q9Z1S0:BUB1B_MOUSE	0.75	0.91	0.84	0.96	0.71	0.87
tr:A1Z6I7:A1Z6I7_DROME	0.13	0.45	0.37	0.78	0.71	0.77
tr:Q7KN56:Q7KN56_DROME	0.13	0.46	0.37	0.78	0.71	0.77
tr:Q21776:Q21776_CAEEL	0.1	0.47	0.16	0.61	0.0	0.18
sp:P47074:MAD3_YEAST	0.1	0.31	0.32	0.71	0.29	0.34
sp:P41695:BUB1_YEAST	0.12	0.5	0.22	0.68	0.14	0.7

BubR1 (FBgn0263855) associated phenotypes

cell cycle defective, male fertile, meiotic cell cycle defective, mitotic cell cycle defective, neuroanatomy defective, partially lethal - majority live

(Information from FlyBase)

bub-1 (WBGene00000275) associated phenotypes

aldicarb hypersensitive, lethal, locomotion variant, sterile

(Information from WormBase)

bub-1 (UniProt:Q21776) annotation

Function: Serine/threonine-protein kinase essential for spindle- assembly checkpoint signaling. Plays a key role in the recruitment of the checkpoint proteins bub-3, mdf-1 and mdf-2 to unattached kinetochores (PubMed:19109417, PubMed:24567362). mdf-1 recruitment is independent of bub-1 kinase activity (PubMed:24567362). In addition, during meiotic anaphase I, controls the recruitment of hcp-1/2 and klp-19 to the ring-shaped domain formed between chromosomes and is involved in chromosome homolog segregation and spindle assembly (PubMed:20729837). Required maternally during embryogenesis and zygotycally for the postembryonic development of several tissues including ventral cord neurons, gonad, intestine and seam cells (PubMed:19526056). (PubMed:19109417, PubMed:19526056, PubMed:20729837, PubMed:24567362).
Subunit: Interacts (via kinase domain) with mdf-1 (via coiled coil domain); the interaction recruits mdf-1 to unattached kinetochores during mitosis and between homologous chromosomes in early anaphase of meiosis I. Interacts with bub-3. (PubMed:24567362).
Subcellular location: Cytoplasm, cell cortex (PubMed:20729837). Nucleus (PubMed:19526056). Chromosome, centromere, kinetochore (PubMed:20729837, PubMed:24567362). Note=Localizes to kinetochores during prophase and metaphase. In metaphase, localizes also to a matrix-like structure around the spindle (PubMed:11402065). In anaphase, localizes only to unattached kinetochores which is kln-1-dependent (PubMed:11402065, PubMed:24567362). Localizes also in a ring-shaped domain between kinetochores, during anaphase of meiosis I which is air-2 and kln- 1-dependent and between sister chromatids during meiosis II, which is kln-1-independent (PubMed:20729837). (PubMed:11402065, PubMed:20729837, PubMed:24567362).
Developmental stage: Expressed troughout embryogenesis mainly in dividing cells. (PubMed:19526056).
Disruption phenotype: RNAi-mediated knockdown in oocytes results in aberrant homologous chromosome segregation during meiosis due to severe pre-anaphase spindle defects characterized by disorganized microtubule bundles. (PubMed:20729837).

(Information from UniProt)

Apoptosis-inducing factor 1, mitochondrial

Best gene implication in ageing for this target family came from gene O95831 via mapping the annotation from Ensembl ENSG00000156709 via mapping the annotation from EntrezGene 9131 via mapping the annotation from GenAgeHuman 0135 annotated in GenAge release 17.

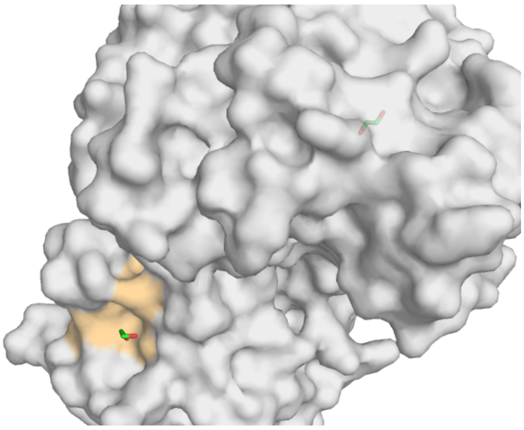

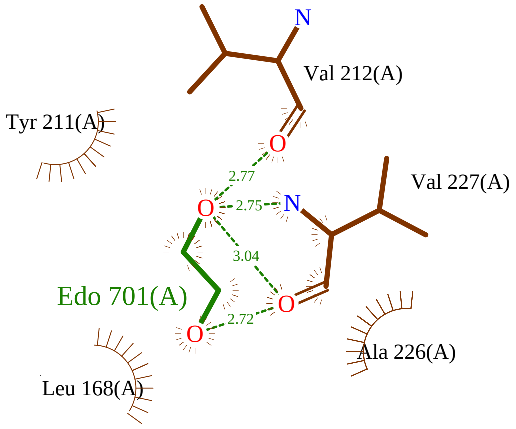

protein	amino acids contacts (binding site)									
PDB:4lii:chainA:O95831	L	Y	V	A	V	G	M	G	K	I
sp:O95831:AIFM1_HUMAN	L	Y	V	A	V	G	M	G	K	I
sp:Q9JM53:AIFM1_RAT	L	Y	V	A	V	G	M	G	K	I
tr:B1AU25:B1AU25_MOUSE	L	Y	V	A	V	G	M	G	K	I
sp:Q9Z0X1:AIFM1_MOUSE	L	Y	V	A	V	G	M	G	K	I
sp:Q9VQ79:AIFM1_DROME	K	F	I	A	V	G	M	S	K	V
tr:E8NH83:E8NH83_DROME	K	F	I	A	V	G	M	S	K	V
tr:Q9N5V9:Q9N5V9_CAEEL	L	F	V	A	L	G	A	E	D	I
tr:K8ESD4:K8ESD4_CAEEL	L	F	V	A	L	G	A	E	D	I
tr:Q8HJ11:Q8HJ11_CAEEL	L	F	V	A	L	G	A	E	D	I
tr:Q9U229:Q9U229_CAEEL	L	F	V	A	L	G	A	E	D	I

	whole protein		domain-based		contact-based	
protein	ident	simil	ident	simil	ident	simil
PDB:4lii:chainA:O95831	1.0	1.0	1.0	1.0	1.0	1.0
sp:O95831:AIFM1_HUMAN	1.0	1.0	1.0	1.0	1.0	1.0
sp:Q9JM53:AIFM1_RAT	0.93	0.98	0.94	0.98	1.0	1.0
tr:B1AU25:B1AU25_MOUSE	0.88	0.95	0.93	0.98	1.0	1.0
sp:Q9Z0X1:AIFM1_MOUSE	0.92	0.98	0.93	0.98	1.0	1.0
sp:Q9VQ79:AIFM1_DROME	0.37	0.66	0.44	0.81	0.5	0.77
tr:E8NH83:E8NH83_DROME	0.36	0.64	0.44	0.81	0.5	0.77
tr:Q9N5V9:Q9N5V9_CAEEL	0.22	0.41	0.35	0.68	0.5	0.69
tr:K8ESD4:K8ESD4_CAEEL	0.29	0.58	0.41	0.79	0.5	0.69
tr:Q8HJ11:Q8HJ11_CAEEL	0.25	0.55	0.4	0.79	0.5	0.69
tr:Q9U229:Q9U229_CAEEL	0.26	0.55	0.41	0.79	0.5	0.69

AIF (FBgn0031392) associated phenotypes

decreased cell death, dominant, immune response defective, increased cell death, partially, small body

(Information from FlyBase)

AIF (UniProt:Q9VQ79) annotation

Function: Probable NADH oxidoreductase (By similarity). Mitochondrial effector of cell death that plays roles in developmentally regulated cell death and normal mitochondrial function. (, PubMed:18309327).

Cofactor: FAD

Subcellular location: Mitochondrion intermembrane space (PubMed:18309327).

Developmental stage: Expressed both maternally and zygotically throughout development with slightly lower levels during pupation. (PubMed:18309327).

Disruption phenotype: Loss of zygotic expression results in decreased embryonic cell death and the persistence of differentiated neuronal cells along the ventral nerve cord at late embryonic stages. Embryos that do hatch undergo growth arrest at early larval stages, accompanied by mitochondrial respiratory dysfunction. (PubMed:18309327).

(Information from UniProt)

Serine/threonine-protein kinase Chk2

Best gene implication in ageing for this target family came from gene O96017 annotated in UniProt release 2014_02. Annotation GO subterm of 7568 (aging) was Non-traceable Author Statement

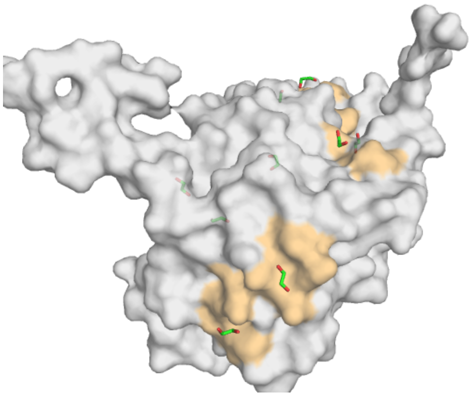

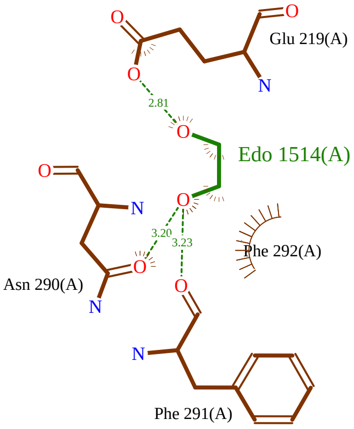

protein	amino acids contacts (binding site)
PDB:2w7x:chainA:O96017	- S V Y P A E R K K K N E L K L P I K I K N F F D A L E L Y Q E N R D L K T D
PDB:2wtd:chainA:O96017	- S V Y P A E R K K K N E L K L P I K I K N F F D A L E L Y Q E N R D L K T D
PDB:2wti:chainA:O96017	- S V Y P A E R K K K N E L K L P I K I K N F F D A L E L Y Q E N R D L K T D
PDB:2wtj:chainA:O96017	M S V Y P A E R K K K N E L K L P I K I K N F F D A L E L Y Q E N R D L K T D
PDB:4a9r:chainA:O96017	- S V Y P A E R K K K N E L K L P I K I K N F F D A L E L Y Q E N R D L K T D
PDB:4a9t:chainA:O96017	M S V Y P A E R K K K N E L K L P I K I K N F F D A L E L Y Q E N R D L K T D
PDB:4a9u:chainA:O96017	M S V Y P A E R K K K N E L K L P I K I K N F F D A L E L Y Q E N R D L K T D
PDB:4bda:chainA:O96017	M S V Y P A E R K K K N E L K L P I K I K N F F D A L E L Y Q E N R D L K T D
PDB:4bdb:chainA:O96017	M S V Y P A E R K K K N E L K L P I K I K N F F D A L E L Y Q E N R D L K T D
PDB:4bdc:chainA:O96017	- S V Y P A E R K K K N E L K L P I K I K N F F D A L E L Y Q E N R D L K T D
PDB:4bdd:chainA:O96017	M S V Y P A E R K K K N E L K L P I K I K N F F D A L E L Y Q E N R D L K T D
PDB:4bde:chainA:O96017	M S V Y P A E R K K K N E L K L P I K I K N F F D A L E L Y Q E N R D L K T D
PDB:4bdf:chainA:O96017	M S V Y P A E R K K K N E L K L P I K I K N F F D A L E L Y Q E N R D L K T D
PDB:4bdg:chainA:O96017	M S V Y P A E R K K K N E L K L P I K I K N F F D A L E L Y Q E N R D L K T D
PDB:4bdh:chainA:O96017	- S V Y P A E R K K K N E L K L P I K I K N F F D A L E L Y Q E N R D L K T D
PDB:4bdi:chainA:O96017	M S V Y P A E R K K K N E L K L P I K I K N F F D A L E L Y Q E N R D L K T D
PDB:4bdk:chainA:O96017	- - V Y P A E R K K K N E L K L P I K I K N F F D A L E L Y Q E N R D L K T D
sp:O96017:CHK2_HUMAN	Q S V Y P A E R K K K N E L K L P I K I K N F F D A L E L Y Q E N R D L K T D
tr:Q9R019:Q9R019_RAT	Q S V Y P E E R K K K S E L K L P I K I K D V F D V L E L Y Q E N R D L K T D
sp:Q9Z265:CHK2_MOUSE	Q S V Y P E E R K K K S E L K L P I K I K D V F D A L E L Y Q E N R D L K T D
tr:Q543W6:Q543W6_MOUSE	Q S V Y P E E R K K K S E L K L P I K I K D V F D A L E L Y Q E N R D L K T D
tr:A4V0X0:A4V0X0_DROME	S I G L P E T T R Q K R E M K L P V R M H D I V D K L E F Y K D R R D L K S D
sp:O61267:LOK_DROME	S I G L P E T T R Q K R E M K L P V R M H D I V D K L E F Y K D R R D L K S D
sp:Q9U1Y5:CHK2_CAEEL	- - - H P E K K S V K D E M K L P V A I Y D W I T V I E Y F L S V R D I K T D
sp:P22216:RAD53_YEAST	K F K Q C E D R T T K G E L Q L P V R L K G F Y E D M E F R K S M R D L K T D

protein	whole protein ident	simil	domain-based ident	simil	contact-based ident	simil
PDB:2w7x:chainA:O96017	1.0	1.0	1.0	1.0	0.99	0.97
PDB:2wtd:chainA:O96017	1.0	1.0	1.0	1.0	0.99	0.97
PDB:2wti:chainA:O96017	1.0	1.0	1.0	1.0	0.99	0.97
PDB:2wtj:chainA:O96017	0.99	1.0	0.99	1.0	1.0	1.0
PDB:4a9r:chainA:O96017	1.0	1.0	1.0	1.0	0.99	0.97
PDB:4a9t:chainA:O96017	1.0	1.0	1.0	1.0	1.0	1.0
PDB:4a9u:chainA:O96017	0.99	1.0	0.99	1.0	1.0	1.0
PDB:4bda:chainA:O96017	0.99	1.0	0.99	1.0	1.0	1.0
PDB:4bdb:chainA:O96017	1.0	1.0	1.0	1.0	1.0	1.0
PDB:4bdc:chainA:O96017	1.0	1.0	1.0	1.0	0.99	0.97
PDB:4bdd:chainA:O96017	1.0	1.0	1.0	1.0	1.0	1.0
PDB:4bde:chainA:O96017	1.0	1.0	1.0	1.0	1.0	1.0
PDB:4bdf:chainA:O96017	1.0	1.0	1.0	1.0	1.0	1.0
PDB:4bdg:chainA:O96017	1.0	1.0	1.0	1.0	1.0	1.0
PDB:4bdh:chainA:O96017	1.0	1.0	1.0	1.0	0.99	0.97
PDB:4bdi:chainA:O96017	1.0	1.0	1.0	1.0	1.0	1.0
PDB:4bdk:chainA:O96017	1.0	1.0	1.0	1.0	0.97	0.94
sp:O96017:CHK2_HUMAN	1.0	1.0	1.0	1.0	0.99	0.99
tr:Q9R019:Q9R019_RAT	0.82	0.92	0.87	0.96	0.82	0.91
sp:Q9Z265:CHK2_MOUSE	0.82	0.92	0.88	0.96	0.83	0.91
tr:Q543W6:Q543W6_MOUSE	0.82	0.92	0.88	0.96	0.83	0.91
tr:A4V0X0:A4V0X0_DROME	0.31	0.64	0.39	0.75	0.33	0.63
sp:O61267:LOK_DROME	0.3	0.63	0.39	0.75	0.33	0.63
sp:Q9U1Y5:CHK2_CAEEL	0.28	0.64	0.34	0.71	0.32	0.56
sp:P22216:RAD53_YEAST	0.16	0.39	0.29	0.64	0.4	0.56

lok (FBgn0019686) associated phenotypes

cell death defective, chemical resistant, conditional, decreased cell death, increased cell death, long lived, maternal effect, radiation sensitive

(Information from FlyBase)

lok (UniProt:O61267) annotation

Function: May have a role in germline establishment. (PubMed:9507063).
Subcellular location: Nucleus speckle(PubMed:9507063). Note=Speckled subnuclear compartment.
Tissue specificity: In stage 3 embryos, both isoforms are expressed in both somatic and pole cell nuclei. Expression in pole cell nuclei is sustained until stage 9 and weakly expressed after pole cell invagination into the abdominal cavity. (PubMed:9507063).
Developmental stage: Expressed both maternally and zygotically in adult females. Levels of the long isoform remain fairly constant from ovaries to embryos, the levels of short isoform decrease dramatically. (PubMed:9507063).

(Information from UniProt)

chk-2 (UniProt:Q9U1Y5) annotation

Function: Serine/threonine-protein kinase which is required for checkpoint-mediated cell cycle arrest, activation of DNA repair and apoptosis in response to the presence of DNA double-strand breaks. May also negatively regulate cell cycle progression during unperturbed cell cycles. Phosphorylates and inhibits cdc25 phosphatase, preventing entry into mitosis. Required for nuclear reorganization and homologous chromosome pairing during meiotic prophase. (PubMed:11086161, PubMed:11158318).
Cofactor: Mg(2+)
Subcellular location: Nucleus (PubMed:11086161, PubMed:11158318).
Tissue specificity: Highly expressed in germline tissue. (PubMed:11086161).
Developmental stage: Low expression in embryos and L2 larvae, high expression in hermaphrodite adults. (PubMed:11086161).

(Information from UniProt)

GTPase KRas

Best gene implication in ageing for this target family came from gene P01119 annotated in UniProt release 2014_02. Annotation GO subterm of 7568 (aging) was Inferred from Mutant Phenotype

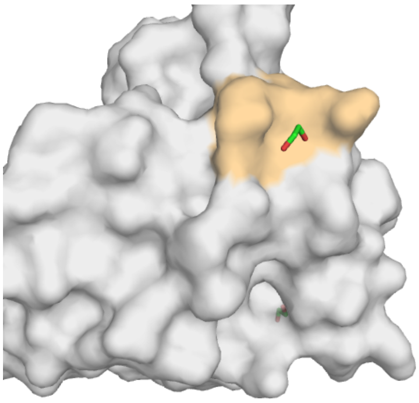

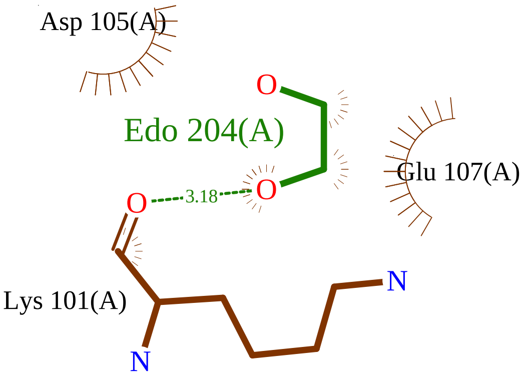

protein	amino acids contacts (binding site)								
PDB:4dsn:chainA:P01116	T	I	E	D	K	R	D	S	E
PDB:4dst:chainA:P01116	T	I	E	D	K	R	D	S	E
tr:L7RSL8:L7RSL8_HUMAN	T	I	E	D	K	R	D	S	E
sp:P01116:RASK_HUMAN	T	I	E	D	K	R	D	S	E
tr:I1SRC5:I1SRC5_HUMAN	T	I	E	D	K	R	D	S	E
sp:P08644:RASK_RAT	T	I	E	D	K	R	D	S	E
tr:A0JN17:A0JN17_RAT	T	I	E	D	K	R	D	S	E
sp:P32883:RASK_MOUSE	T	I	E	D	K	R	D	S	E
tr:Q0VDV7:Q0VDV7_MOUSE	T	I	E	D	K	R	D	S	E
tr:Q5J7N1:Q5J7N1_MOUSE	T	I	E	D	K	R	D	S	E
sp:P08646:RAS1_DROME	T	I	E	D	K	R	D	A	E
sp:P22981:LET60_CAEEL	T	I	E	D	R	R	D	S	D
sp:P01119:RAS1_YEAST	T	I	E	D	Q	R	D	S	D

	whole protein		domain-based		contact-based	
protein	ident	simil	ident	simil	ident	simil
PDB:4dsn:chainA:P01116	0.91	0.96	0.95	0.98	1.0	1.0
PDB:4dst:chainA:P01116	0.91	0.96	0.95	0.98	1.0	1.0
tr:L7RSL8:L7RSL8_HUMAN	1.0	1.0	1.0	1.0	1.0	1.0
sp:P01116:RASK_HUMAN	1.0	1.0	1.0	1.0	1.0	1.0
tr:I1SRC5:I1SRC5_HUMAN	0.64	0.64	1.0	1.0	1.0	1.0
sp:P08644:RASK_RAT	0.99	1.0	0.99	1.0	1.0	1.0
tr:A0JN17:A0JN17_RAT	0.89	0.95	0.94	0.98	1.0	1.0
sp:P32883:RASK_MOUSE	0.99	1.0	0.99	1.0	1.0	1.0
tr:Q0VDV7:Q0VDV7_MOUSE	0.99	1.0	0.99	1.0	1.0	1.0
tr:Q5J7N1:Q5J7N1_MOUSE	0.88	0.95	0.94	0.98	1.0	1.0
sp:P08646:RAS1_DROME	0.76	0.91	0.81	0.93	0.89	0.88
sp:P22981:LET60_CAEEL	0.75	0.89	0.77	0.92	0.78	0.92
sp:P01119:RAS1_YEAST	0.35	0.51	0.6	0.86	0.78	0.89

Ras85D (FBgn0003205) associated phenotypes

RU486 conditional, cell autonomous, cell death defective, cell non-autonomous, cell polarity defective, cell size defective, conditional, decreased cell death, decreased cell number, decreased cell size, developmental rate defective, dominant, endocytosis defective, female semi-sterile, germline clone, heat sensitive, hyperplasia, increased cell death, increased cell growth, increased cell number, increased cell size, large body, learning defective, lethal - all die before end of P-stage, lethal - all die before end of pupal stage, majority die during P-stage, maternal effect, melanotic mass phenotype, mitotic cell cycle defective, neoplasia, neuroanatomy defective, neurophysiology defective, partially, partially lethal - majority die, short lived, size defective, small body, somatic clone, some die during P-stage, some die during pharate adult stage, some die during pupal stage, temperature conditional,

terminal phenotype, tumorigenic

(Information from FlyBase)

Ras85D (UniProt:P08646) annotation

Function: May mediate a signal that determines the fate of photoreceptor cells in the developing compound eye. Ras proteins bind GDP/GTP and possess intrinsic GTPase activity.

Enzyme regulation: Alternates between an inactive form bound to GDP and an active form bound to GTP. Activated by a guanine nucleotide-exchange factor (GEF) and inactivated by a GTPase- activating protein (GAP).

Subcellular location: Cell membrane (PubMed:18503409); Lipid-anchor (PubMed:18503409); Cytoplasmic side (PubMed:18503409). Note=Loss of prenylation causes protein location to the cytoplasm.

Tissue specificity: Expressed in the posterior termini of the embryo, restricted mainly to the embryonic central nervous system, and in the eye imaginal disk. (PubMed:7873789).

(Information from UniProt)

let-60 (WBGene00002335) associated phenotypes

AWA odorant chemotaxis defective, AWC odorant chemotaxis defective, L2 arrest, L3 arrest, antibody staining reduced, antibody staining variant, axon outgrowth variant, bloated, cell differentiation variant, egg laying defective, egg laying imipramine resistant, egg laying variant, germ cell development variant, gonad arm morphology variant, lethal, locomotion variant, male mating defective, mid larval lethal, multivulva, neuron degeneration, oocyte accumulation, oocytes small, organism starvation response variant, protein expression absent, reduced brood size, rod like larval lethal, sterile, unfertilized oocytes laid, vulval cell induction increased, vulvaless

(Information from WormBase)

let-60 (UniProt:P22981) annotation

Function: The level of let-60 controls the switch between vulval and hypodermal cell fates during C.elegans vulval induction. May stimulate the guanine nucleotide exchange factor (GEF) activity of rap-1.

Subunit: Interacts with soc-2. (PubMed:9674433).

Subcellular location: Cell membrane; Lipid-anchor.

(Information from UniProt)

Thymidylate synthase

Best gene implication in ageing for this target family came from gene P45352 annotated in UniProt release 2014_02. Annotation GO 7568 (aging) was Inferred from Expression Pattern

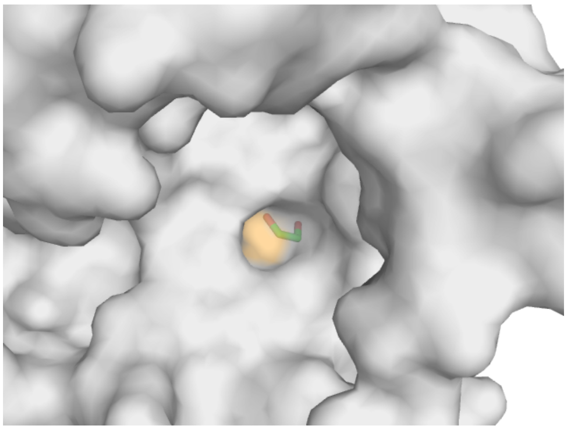

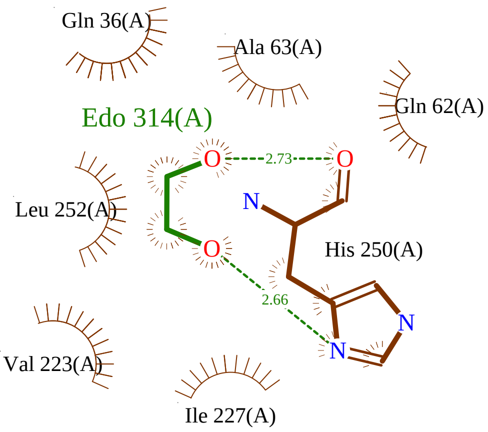

protein	amino acids contacts (binding site)
PDB:2onb:chainA:P04818	Q Q A V I H L
sp:P04818:TYSY_HUMAN	Q Q A V I H L
tr:Q53Y97:Q53Y97_HUMAN	Q Q A V I H L
sp:P45352:TYSY_RAT	Q Q A V I H L
sp:P07607:TYSY_MOUSE	Q Q A V I H L
tr:Q544L2:Q544L2_MOUSE	Q Q A V I H L
tr:Q541C9:Q541C9_DROME	L Q M V I H M
sp:O76511:TYSY_DROME	L Q M V I H M
tr:Q9Y052:Q9Y052_CAEEL	Q Q S V L H L
tr:Q9N588:Q9N588_CAEEL	Q Q S V L H L
sp:P06785:TYSY_YEAST	L Q L V I H L

	whole protein		domain-based		contact-based	
protein	ident	simil	ident	simil	ident	simil
PDB:2onb:chainA:P04818	0.98	0.99	0.98	0.99	1.0	1.0
sp:P04818:TYSY_HUMAN	1.0	1.0	1.0	1.0	1.0	1.0
tr:Q53Y97:Q53Y97_HUMAN	1.0	1.0	1.0	1.0	1.0	1.0
sp:P45352:TYSY_RAT	0.88	0.95	0.92	0.98	1.0	1.0
sp:P07607:TYSY_MOUSE	0.88	0.95	0.93	0.98	1.0	1.0
tr:Q544L2:Q544L2_MOUSE	0.88	0.95	0.93	0.98	1.0	1.0
tr:Q541C9:Q541C9_DROME	0.62	0.84	0.65	0.86	0.57	0.67
sp:O76511:TYSY_DROME	0.62	0.84	0.65	0.86	0.57	0.67
tr:Q9Y052:Q9Y052_CAEEL	0.61	0.84	0.65	0.87	0.71	0.84
tr:Q9N588:Q9N588_CAEEL	0.61	0.83	0.65	0.87	0.71	0.84
sp:P06785:TYSY_YEAST	0.57	0.78	0.61	0.84	0.71	0.68

Ts (FBgn0024920) associated phenotypes

neuroanatomy defective

(Information from FlyBase)

Ts (UniProt:O76511) annotation

Pathway: Pyrimidine metabolism; dTTP biosynthesis.
Subunit: Homodimer.

(Information from UniProt)

WBGene00022455 associated phenotypes

lethal, sterile

(Information from WormBase)

Phosphoenolpyruvate carboxykinase, cytosolic [GTP]

Best gene implication in ageing for this target family came from gene Q9Z2V4 via mapping the annotation from Ensembl ENSMUSG00000027513 via mapping the annotation from EntrezGene 18534 via mapping the annotation from GenAgeModels 0497 annotated in GenAge release 17.

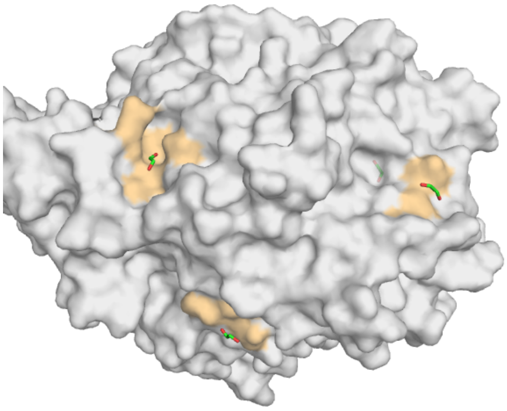

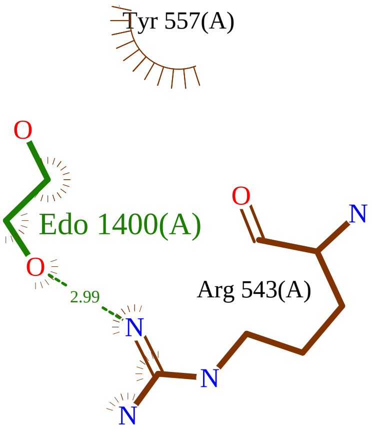

protein	amino acids contacts (binding site)																																	
PDB:2qey:chainA:P07379	F	N	R	A	P	K	I	E	F	I	K	E	G	K	K	F	R	S	C	P	I	R	P	A	A	S	W	I	P	K	I	R	Y	F
PDB:3moe:chainA:P07379	F	N	R	A	P	K	I	E	F	I	K	E	G	K	K	F	R	S	C	P	I	R	P	A	A	S	W	I	P	K	I	R	Y	F
PDB:3mof:chainA:P07379	F	N	R	A	P	K	I	E	F	I	K	E	G	K	K	F	R	S	C	P	I	R	P	A	A	S	W	I	P	K	I	R	Y	F
PDB:3moh:chainA:P07379	F	N	R	A	P	K	I	E	F	I	K	E	G	K	K	F	R	S	C	P	I	R	P	A	A	S	W	I	P	K	I	R	Y	F
sp:P35558:PCKGC_HUMAN	F	N	R	A	P	K	I	E	F	V	K	E	G	K	K	F	R	S	C	P	I	R	P	A	A	S	W	I	P	K	I	R	Y	F
sp:P07379:PCKGC_RAT	F	N	R	A	P	K	I	E	F	I	K	E	G	K	K	F	R	S	C	P	I	R	P	A	A	S	W	I	P	K	I	R	Y	F
sp:Q9Z2V4:PCKGC_MOUSE	F	N	R	A	P	K	I	E	F	I	K	E	G	K	K	F	R	S	C	P	I	R	P	E	A	S	W	I	P	K	I	R	Y	F
tr:Q7JXB5:Q7JXB5_DROME	Y	C	R	E	P	K	I	Q	F	V	K	E	G	K	K	F	R	S	C	P	I	R	P	S	A	D	W	I	P	K	I	R	R	F
sp:P20007:PCKG_DROME	F	G	R	Q	P	K	I	E	F	V	R	L	G	K	K	F	R	A	C	P	I	R	P	A	A	D	W	I	P	K	I	R	H	F
tr:Q7JKI2:Q7JKI2_CAEEL	F	K	R	S	P	K	I	D	F	V	R	E	G	K	K	F	R	N	C	P	I	R	P	Q	T	S	W	V	P	K	I	R	T	Y
tr:O44906:O44906_CAEEL	F	K	R	S	P	K	N	D	F	V	R	E	G	K	K	F	R	K	C	P	I	R	P	E	S	S	W	V	P	K	I	R	Y	Y
tr:O02286:O02286_CAEEL	F	K	R	S	P	K	I	D	F	V	R	E	G	K	K	F	R	N	C	P	I	R	P	Q	T	S	W	V	P	K	I	R	T	Y

	whole protein		domain-based		contact-based	
protein	ident	simil	ident	simil	ident	simil
PDB:2qey:chainA:P07379	1.0	1.0	1.0	1.0	1.0	1.0
PDB:3moe:chainA:P07379	1.0	1.0	1.0	1.0	1.0	1.0
PDB:3mof:chainA:P07379	1.0	1.0	1.0	1.0	1.0	1.0
PDB:3moh:chainA:P07379	1.0	1.0	1.0	1.0	1.0	1.0
sp:P35558:PCKGC_HUMAN	0.91	0.98	0.91	0.98	0.97	0.99
sp:P07379:PCKGC_RAT	1.0	1.0	1.0	1.0	1.0	1.0
sp:Q9Z2V4:PCKGC_MOUSE	0.98	0.99	0.97	0.99	0.97	0.97
tr:Q7JXB5:Q7JXB5_DROME	0.62	0.85	0.64	0.88	0.76	0.84
sp:P20007:PCKG_DROME	0.61	0.84	0.65	0.88	0.76	0.83
tr:Q7JKI2:Q7JKI2_CAEEL	0.56	0.84	0.59	0.87	0.68	0.83
tr:O44906:O44906_CAEEL	0.54	0.81	0.58	0.85	0.68	0.78
tr:O02286:O02286_CAEEL	0.54	0.82	0.59	0.87	0.68	0.83

Pepck (UniProt:P20007) annotation

Function: Catalyzes the conversion of oxaloacetate (OAA) to phosphoenolpyruvate (PEP), the rate-limiting step in the metabolic pathway that produces glucose from lactate and other precursors derived from the citric acid cycle.

Cofactor: Mn(2+)Note=Binds 1 Mn(2+) ion per subunit. ;

Pathway: Carbohydrate biosynthesis; gluconeogenesis.

Subunit: Monomer.

(Information from UniProt)

Alcohol dehydrogenase 4

Best gene implication in ageing for this target family came from gene A1L128 via mapping the annotation from RGD 71028 annotated in RGD 2014-03-11. Annotation GO 7568 (aging) was Inferred from Expression Pattern

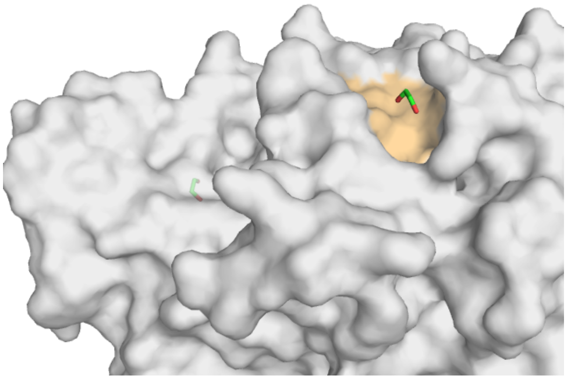

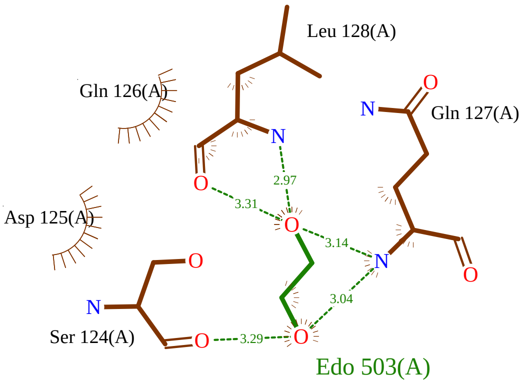

protein	amino acids contacts (binding site)									
PDB:3cos:chainA:P08319	S	D	Q	Q	L	F	P	I	E	A
sp:P08319:ADH4_HUMAN	S	D	Q	Q	L	F	P	I	E	A
tr:A1L128:A1L128_RAT	I	D	Q	E	L	F	P	V	Q	A
sp:Q9QYY9:ADH4_MOUSE	I	D	Q	E	L	F	P	V	Q	A
sp:P46415:ADHX_DROME	Q	G	A	G	V	W	P	I	N	S
sp:Q17335:ADHX_CAEEL	Q	G	N	G	F	W	P	F	H	Q
tr:Q965R0:Q965R0_CAEEL	Q	G	D	G	F	W	P	F	H	Q
sp:P32771:FADH_YEAST	Q	G	K	G	V	F	P	I	K	D

	whole protein		domain-based		contact-based	
protein	ident	simil	ident	simil	ident	simil
PDB:3cos:chainA:P08319	0.99	1.0	1.0	1.0	1.0	1.0
sp:P08319:ADH4_HUMAN	1.0	1.0	1.0	1.0	1.0	1.0
tr:A1L128:A1L128_RAT	0.72	0.92	0.74	0.92	0.6	0.79
sp:Q9QYY9:ADH4_MOUSE	0.71	0.91	0.74	0.92	0.6	0.79
sp:P46415:ADHX_DROME	0.55	0.85	0.59	0.85	0.2	0.59
sp:Q17335:ADHX_CAEEL	0.51	0.82	0.53	0.82	0.1	0.64
tr:Q965R0:Q965R0_CAEEL	0.33	0.55	0.31	0.5	0.1	0.63
sp:P32771:FADH_YEAST	0.46	0.78	0.51	0.8	0.3	0.6

Fdh (FBgn0011768) associated phenotypes
heat sensitive, memory defective

(Information from FlyBase)

Fdh (UniProt:P46415) annotation
Function: Class-III ADH is remarkably ineffective in oxidizing ethanol, but it readily catalyzes the oxidation of long-chain primary alcohols and the oxidation of S-(hydroxymethyl) glutathione.
Cofactor:Zn(2+)Note=Binds 2 Zn(2+) ions per subunit. ;

(Information from UniProt)

H24K24.3 (UniProt:Q17335) annotation
Function: Class-III ADH is remarkably ineffective in oxidizing ethanol, but it readily catalyzes the oxidation of long-chain primary alcohols and the oxidation of S-(hydroxymethyl) glutathione.
Cofactor: Zn(2+)Note=Binds 2 Zn(2+) ions per subunit. ;
Subunit: Homodimer.
Subcellular location: Cytoplasm

(Information from UniProt)

CELE$_Y 50D4C.2(UniProt:Q965R0)annotation$
Cofactor: Zn(2+) Evidence=(RuleBase:RU361277);

(Information from UniProt)

Poly [ADP-ribose] polymerase 1

Best gene implication in ageing for this target family came from gene Q921K2 via mapping the annotation from Ensembl ENSMUSG00000026496 via mapping the annotation from EntrezGene 11545 via mapping the annotation from GenAgeModels 1010 annotated in GenAge release 17.

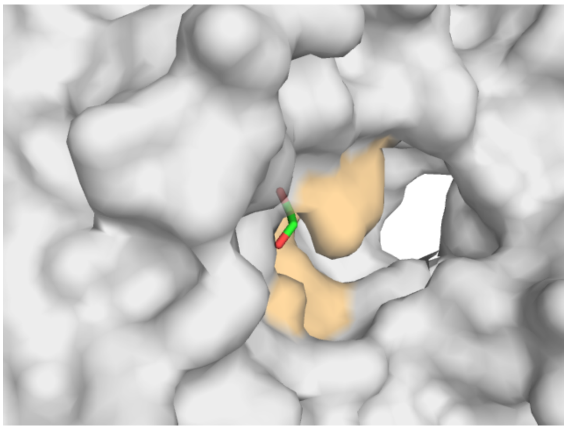

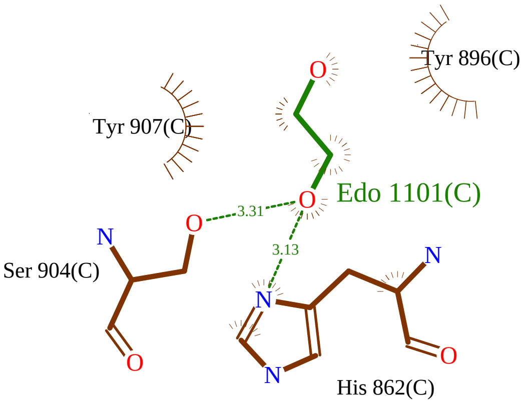

protein	amino acids contacts (binding site)
PDB:4dqy:chainC:P09874	H G Y S Y E
sp:P09874:PARP1_HUMAN	H G Y S Y E
sp:P27008:PARP1_RAT	H G Y S Y E
tr:Q921K2:Q921K2_MOUSE	H G Y S Y E
sp:P35875:PARP_DROME	H G Y S Y E
sp:Q9N4H4:PME1_CAEEL	H G Y S Y E

	whole protein		domain-based		contact-based	
protein	ident	simil	ident	simil	ident	simil
PDB:4dqy:chainC:P09874	1.0	1.0	1.0	1.0	1.0	1.0
sp:P09874:PARP1_HUMAN	1.0	1.0	1.0	1.0	1.0	1.0
sp:P27008:PARP1_RAT	0.92	0.97	0.96	0.99	1.0	1.0
tr:Q921K2:Q921K2_MOUSE	0.92	0.98	0.96	0.99	1.0	1.0
sp:P35875:PARP_DROME	0.41	0.77	0.62	0.88	1.0	1.0
sp:Q9N4H4:PME1_CAEEL	0.29	0.63	0.46	0.8	1.0	1.0

Parp (FBgn0010247) associated phenotypes

aging defective, heat sensitive, heat stress response defective, immune response defective, increased cell death, lethal - all die before end of pupal stage, non-enhancer of variegation, non-suppressor of variegation, planar polarity defective, some die during pupal stage

(Information from FlyBase)

Parp (UniProt:P35875) annotation

Function: Poly[ADP-ribose] polymerase modifies various nuclear proteins by poly(ADP-ribosyl)ation. The modification is dependent on DNA and is involved in the regulation of various important cellular processes such as differentiation, proliferation, and tumor transformation and also in the regulation of the molecular events involved in the recovery of cell from DNA damage. Plays a fundamental role in organizing chromatin on a global scale; isoform e autoregulates Parp transcription by influencing the chromatin structure of its heterochromatic environment. (PubMed:12183365).
Subcellular location: Nucleus ECO:0000255—PROSITE- ProRule:PRU00264, PubMed:12183365). Nucleus, nucleolus (PubMed:12183365). Note=Highly enriched in nucleoli, heterochromatic chromosomal regions, and diverse euchromatic sites in the cells of most embryonic and adult tissues.
Tissue specificity: Expressed in adult female oocytes, anal plates of stage 12 embryos and in cells around the central nervous system in later embryos. (PubMed:9565614).
Developmental stage: Expressed both maternally and zygotically in embryos, pupae and adults. Expression is highest in embryos. (PubMed:12183365, PubMed:9565614).
Miscellaneous: The ADP-D-ribosyl group of NAD(+) is transferred to an acceptor carboxyl group

on a histone or the enzyme itself, and further ADP-ribosyl groups are transferred to the 2'-position of the terminal adenosine moiety, building up a polymer with an average chain length of 20-30 units.

(Information from UniProt)

pme-1 (UniProt:Q9N4H4) annotation

Function: Poly[ADP-ribose] polymerase modifies various nuclear proteins by poly(ADP-ribosyl)ation, a post-translational modification synthesized after DNA damage that appears as an obligatory step in a detection/signaling pathway leading to the reparation of DNA strand breaks and programmed cell death. Involved in protection of the genome against mutations, probably via some participation in DNA repair. (PubMed:12600937).

Subcellular location: Nucleus ECO:0000305.

Developmental stage: Predominantly expressed at early embryonic stages and later in L4 and adult stages. (PubMed:12145714).

(Information from UniProt)

Polyubiquitin-C

Best gene implication in ageing for this target family came from gene H9BVD7 via mapping the annotation from Ensembl FBgn0029856 via mapping the annotation from FlyBase FBgn0029856 annotated in FlyBase 2014-03-11. Annotation GO 8340 (determination of adult lifespan) was Inferred from Mutant Phenotype

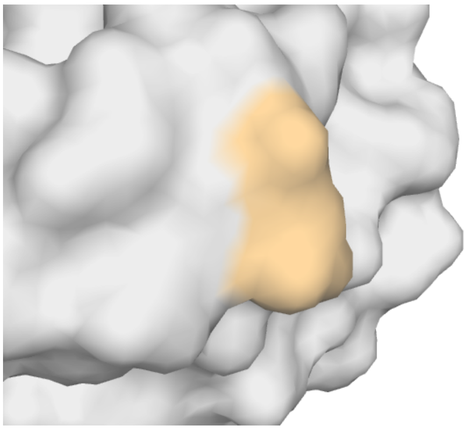

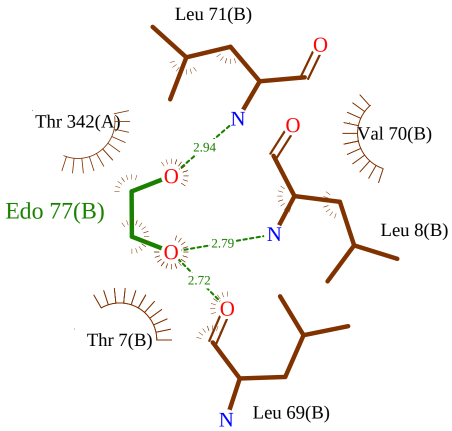

protein	amino acids contacts (binding site)
PDB:2znv:chainB:P0CG50	T L D G P Q L V L
PDB:2znv:chainC:P0CG50	T L D G P Q L V L
tr:J3QS39:J3QS39_HUMAN	T L D G P Q L V L
tr:F5H6Q2:F5H6Q2_HUMAN	T L D G P Q L V L
tr:F5H2Z3:F5H2Z3_HUMAN	T L D G P Q L V L
tr:F5GYU3:F5GYU3_HUMAN	T L D G P Q L V L
tr:F5H265:F5H265_HUMAN	T L D G P Q L V L
tr:F5H388:F5H388_HUMAN	T L D G P Q L V L
tr:B4DV12:B4DV12_HUMAN	T L D G P Q L V L
tr:F5GXK7:F5GXK7_HUMAN	T L D G P Q L V L
tr:F5H747:F5H747_HUMAN	T L D G P Q L V L
tr:J3QKN0:J3QKN0_HUMAN	T L D G P Q L V L
sp:P0CG47:UBB_HUMAN	T L D G P Q L V L
tr:Q5PY61:Q5PY61_HUMAN	T L D G P Q L V L
tr:Q5U5U6:Q5U5U6_HUMAN	T L D G P Q L V L
tr:Q9UFQ0:Q9UFQ0_HUMAN	T L D G P Q L V L
tr:Q96C32:Q96C32_HUMAN	T L D G P Q L V L
tr:F5H041:F5H041_HUMAN	T L D G P Q L V L
sp:P0CG48:UBC_HUMAN	T L D G P Q L V L
tr:Q6P7R7:Q6P7R7_RAT	T L D G P Q L V L
sp:P62986:RL40_RAT	T L D G P Q L V L
tr:A5JUZ1:A5JUZ1_MOUSE	T L D G P Q L V L
tr:E9Q9J0:E9Q9J0_MOUSE	T L D G P Q L V L
tr:Q66JP1:Q66JP1_MOUSE	T L D G P Q L V L
sp:P62984:RL40_MOUSE	T L D G P Q L V L
tr:Q922B0:Q922B0_MOUSE	T L D G P Q L V L
tr:E9Q5F6:E9Q5F6_MOUSE	T L D G P Q L V L
tr:Q5SX22:Q5SX22_MOUSE	T L D G P Q L V L
tr:Q3TH47:Q3TH47_MOUSE	T L D G P Q L V L
tr:Q78XY9:Q78XY9_MOUSE	T L D G P Q L V L
sp:P0CG49:UBB_MOUSE	T L D G P Q L V L
tr:Q8R0Z9:Q8R0Z9_MOUSE	T L D G P Q L V L
tr:Q922Z8:Q922Z8_MOUSE	T L D G P Q L V L
tr:Q8VC46:Q8VC46_MOUSE	T L D G P Q L V L
sp:P0CG50:UBC_MOUSE	T L D G P Q L V L
tr:Q7JPZ2:Q7JPZ2_DROME	T L D G P Q L V L
tr:Q7JPZ3:Q7JPZ3_DROME	T L D G P Q L V L
tr:Q27927:Q27927_DROME	T L D G P Q L V L
tr:Q24577:Q24577_DROME	T L D G P Q L V L
tr:Q24575:Q24575_DROME	T L D G P Q L V L
tr:Q24576:Q24576_DROME	T L D G P Q L V L
tr:Q7JYK1:Q7JYK1_DROME	T L D G P Q L V L
sp:P18101:RL40_DROME	T L D G P Q L V L
tr:H9BVD7:H9BVD7_DROME	T L D G P Q L V L
tr:H9BVD5:H9BVD5_DROME	T L D G P Q L V L
tr:H9BVD4:H9BVD4_DROME	T L D G P Q L V L
tr:H9BVD3:H9BVD3_DROME	T L D G P Q L V L
tr:H9BVD2:H9BVD2_DROME	T L D G P Q L V L
sp:P49632:RL40_CAEEL	T L D G P Q L V L
tr:G8JY83:G8JY83_CAEEL	T L D G P Q L V L
sp:P0CG71:UBIQ1_CAEEL	T L D G P Q L V L
tr:E9PA11:E9PA11_YEASX	T L D G P Q - - -
sp:P0CH09:RL402_YEAST	T L D G P Q L V L
sp:P0CH08:RL401_YEAST	T L D G P Q L V L

protein	whole protein ident	simil	domain-based ident	simil	contact-based ident	simil
PDB:2znv:chainB:P0CG50	0.99	1.0	0.99	1.0	1.0	1.0
PDB:2znv:chainC:P0CG50	0.99	0.99	1.0	1.0	1.0	1.0
tr:J3QS39:J3QS39_HUMAN	0.13	0.13	1.0	1.0	1.0	1.0
tr:F5H6Q2:F5H6Q2_HUMAN	0.17	0.17	1.0	1.0	1.0	1.0
tr:F5H2Z3:F5H2Z3_HUMAN	0.19	0.19	1.0	1.0	1.0	1.0
tr:F5GYU3:F5GYU3_HUMAN	0.18	0.18	1.0	1.0	1.0	1.0
tr:F5H265:F5H265_HUMAN	0.2	0.2	1.0	1.0	1.0	1.0
tr:F5H388:F5H388_HUMAN	0.21	0.21	1.0	1.0	1.0	1.0
tr:B4DV12:B4DV12_HUMAN	0.21	0.21	1.0	1.0	1.0	1.0
tr:F5GXK7:F5GXK7_HUMAN	0.23	0.23	1.0	1.0	1.0	1.0
tr:F5H747:F5H747_HUMAN	0.22	0.22	1.0	1.0	1.0	1.0
tr:J3QKN0:J3QKN0_HUMAN	0.24	0.26	1.0	1.0	1.0	1.0
sp:P0CG47:UBB_HUMAN	0.23	0.25	1.0	1.0	1.0	1.0
tr:Q5PY61:Q5PY61_HUMAN	0.23	0.25	1.0	1.0	1.0	1.0
tr:Q5U5U6:Q5U5U6_HUMAN	0.23	0.25	1.0	1.0	1.0	1.0
tr:Q9UFQ0:Q9UFQ0_HUMAN	0.24	0.26	1.0	1.0	1.0	1.0
tr:Q96C32:Q96C32_HUMAN	0.21	0.23	1.0	1.0	1.0	1.0
tr:F5H041:F5H041_HUMAN	0.33	0.35	0.33	0.33	1.0	1.0
sp:P0CG48:UBC_HUMAN	0.41	0.42	0.33	0.33	1.0	1.0
tr:Q6P7R7:Q6P7R7_RAT	0.12	0.15	1.0	1.0	1.0	1.0
sp:P62986:RL40_RAT	0.12	0.15	1.0	1.0	1.0	1.0
tr:A5JUZ1:A5JUZ1_MOUSE	0.1	0.1	1.0	1.0	1.0	1.0
tr:E9Q9J0:E9Q9J0_MOUSE	0.11	0.12	1.0	1.0	1.0	1.0
tr:Q66JP1:Q66JP1_MOUSE	0.12	0.15	1.0	1.0	1.0	1.0
sp:P62984:RL40_MOUSE	0.12	0.15	1.0	1.0	1.0	1.0
tr:Q922B0:Q922B0_MOUSE	0.28	0.28	1.0	1.0	1.0	1.0
tr:E9Q5F6:E9Q5F6_MOUSE	0.24	0.26	1.0	1.0	1.0	1.0
tr:Q5SX22:Q5SX22_MOUSE	0.23	0.25	1.0	1.0	1.0	1.0
tr:Q3TH47:Q3TH47_MOUSE	0.38	0.38	1.0	1.0	1.0	1.0
tr:Q78XY9:Q78XY9_MOUSE	0.21	0.23	1.0	1.0	1.0	1.0
sp:P0CG49:UBB_MOUSE	0.21	0.23	1.0	1.0	1.0	1.0
tr:Q8R0Z9:Q8R0Z9_MOUSE	0.48	0.48	1.0	1.0	1.0	1.0
tr:Q922Z8:Q922Z8_MOUSE	0.79	0.79	1.0	1.0	1.0	1.0
tr:Q8VC46:Q8VC46_MOUSE	0.9	0.9	1.0	1.0	1.0	1.0
sp:P0CG50:UBC_MOUSE	1.0	1.0	1.0	1.0	1.0	1.0
tr:Q7JPZ2:Q7JPZ2_DROME	0.1	0.1	0.96	0.98	1.0	1.0
tr:Q7JPZ3:Q7JPZ3_DROME	0.1	0.1	0.99	0.99	1.0	1.0
tr:Q27927:Q27927_DROME	0.1	0.1	0.99	0.99	1.0	1.0
tr:Q24577:Q24577_DROME	0.1	0.1	0.99	1.0	1.0	1.0
tr:Q24575:Q24575_DROME	0.1	0.1	0.97	0.99	1.0	1.0
tr:Q24576:Q24576_DROME	0.1	0.1	0.97	0.98	1.0	1.0
tr:Q7JYK1:Q7JYK1_DROME	0.12	0.15	1.0	1.0	1.0	1.0
sp:P18101:RL40_DROME	0.12	0.15	1.0	1.0	1.0	1.0
tr:H9BVD7:H9BVD7_DROME	0.28	0.31	0.95	0.99	1.0	1.0
tr:H9BVD5:H9BVD5_DROME	0.29	0.31	0.96	0.99	1.0	1.0
tr:H9BVD4:H9BVD4_DROME	0.28	0.31	0.96	0.99	1.0	1.0
tr:H9BVD3:H9BVD3_DROME	0.28	0.31	0.96	0.99	1.0	1.0
tr:H9BVD2:H9BVD2_DROME	0.29	0.31	0.96	0.99	1.0	1.0
sp:P49632:RL40_CAEEL	0.12	0.15	0.99	1.0	1.0	1.0
tr:G8JY83:G8JY83_CAEEL	0.16	0.18	0.99	1.0	1.0	1.0
sp:P0CG71:UBIQ1_CAEEL	0.13	0.14	0.99	1.0	1.0	1.0
tr:E9PA11:E9PA11_YEASX	0.06	0.06	0.57	0.6	0.67	0.25
sp:P0CH09:RL402_YEAST	0.12	0.15	0.96	0.99	1.0	1.0
sp:P0CH08:RL401_YEAST	0.12	0.15	0.96	0.99	1.0	1.0

Ubi-p63E (FBgn0003943) associated phenotypes

cell autonomous, increased cell death, male limited, meiotic cell cycle defective, somatic clone

(Information from FlyBase)

RpL40 (FBgn0003941) associated phenotypes

partially lethal - majority die

(Information from FlyBase)

RpL40 (UniProt:P18101) annotation
Function: Ubiquitin: exists either covalently attached to another protein, or free (unanchored). When covalently bound, it is conjugated to target proteins via an isopeptide bond either as a monomer (monoubiquitin), a polymer linked via different Lys residues of the ubiquitin (polyubiquitin chains) or a linear polymer linked via the initiator Met of the ubiquitin (linear polyubiquitin chains). Polyubiquitin chains, when attached to a target protein, have different functions depending on the Lys residue of the ubiquitin that is linked: Lys-48-linked is involved in protein degradation via the proteasome. Linear polymer chains formed via attachment by the initiator Met lead to cell signaling. Ubiquitin is usually conjugated to Lys residues of target proteins, however, in rare cases, conjugation to Cys or Ser residues has been observed. When polyubiquitin is free (unanchored-polyubiquitin), it also has distinct roles, such as in activation of protein kinases, and in signaling (By similarity).
Function: 60S ribosomal protein L40: component of the 60S subunit of the ribosome.
Subunit: 60S ribosomal protein is part of the 60S ribosomal subunit.
Subcellular location: Ubiquitin: Cytoplasm Nucleus
Subcellular location: 60S ribosomal protein L40: Cytoplasm
Miscellaneous: In Drosophila ubiquitin is encoded by 3 different genes. RpL40 and RpS27A genes code for a single copy of ubiquitin fused to the ribosomal proteins L40 and S27a, respectively. Ubi-p63E gene codes for a polyubiquitin precursor with 10 exact head to tail repeats.
Miscellaneous: For a better understanding, features related to ubiquitin are only indicated for the first chain.

(Information from UniProt)

ubq-2 (UniProt:P49632) annotation
Function: Ubiquitin: exists either covalently attached to another protein, or free (unanchored). When covalently bound, it is conjugated to target proteins via an isopeptide bond either as a monomer (monoubiquitin), a polymer linked via different Lys residues of the ubiquitin (polyubiquitin chains) or a linear polymer linked via the initiator Met of the ubiquitin (linear polyubiquitin chains). Polyubiquitin chains, when attached to a target protein, have different functions depending on the Lys residue of the ubiquitin that is linked: Lys-48-linked is involved in protein degradation via the proteasome. Linear polymer chains formed via attachment by the initiator Met lead to cell signaling. Ubiquitin is usually conjugated to Lys residues of target proteins, however, in rare cases, conjugation to Cys or Ser residues has been observed. When polyubiquitin is free (unanchored-polyubiquitin), it also has distinct roles, such as in activation of protein kinases, and in signaling (By similarity).
Function: 60S ribosomal protein L40: component of the 60S subunit of the ribosome.
Subunit: Ribosomal protein L40 is part of the 60S ribosomal subunit.
Subcellular location: Ubiquitin: Cytoplasm Nucleus
Subcellular location: 60S ribosomal protein L40: Cytoplasm
Miscellaneous: In C.elegans ubiquitin is encoded by 2 different genes. ubq-2 gene codes for a single copy of ubiquitin fused to the ribosomal proteins L40. ubq-1 gene codes for a polyubiquitin precursor with exact head to tail repeats.
Miscellaneous: For a better understanding, features related to ubiquitin are only indicated for the first chain.

(Information from UniProt)

ubq-1 (UniProt:P0CG71) annotation
Function: Ubiquitin exists either covalently attached to another protein, or free (unanchored). When covalently bound, it is conjugated to target proteins via an isopeptide bond either as a monomer (monoubiquitin), a polymer linked via different Lys residues of the ubiquitin (polyubiquitin chains) or a linear polymer linked via the initiator Met of the ubiquitin (linear polyubiquitin chains). Polyubiquitin chains, when attached to a target protein, have different functions depending

on the Lys residue of the ubiquitin that is linked: Lys-48-linked is involved in protein degradation via the proteasome. Linear polymer chains formed via attachment by the initiator Met lead to cell signaling. Ubiquitin is usually conjugated to Lys residues of target proteins, however, in rare cases, conjugation to Cys or Ser residues has been observed. When polyubiquitin is free (unanchored-polyubiquitin), it also has distinct roles, such as in activation of protein kinases, and in signaling (By similarity).
Subcellular location: Ubiquitin: Cytoplasm Nucleus
Miscellaneous: In C.elegans ubiquitin is encoded by 2 different genes. ubq-2 gene codes for a single copy of ubiquitin fused to the ribosomal proteins L40. ubq-1 gene codes for a polyubiquitin precursor with exact head to tail repeats.
Miscellaneous: For the sake of clarity sequence features are annotated only for the first chain, and are not repeated for each of the following chains.

(Information from UniProt)

Fibroblast growth factor receptor 1

Best gene implication in ageing for this target family came from gene P11362 via mapping the annotation from Ensembl ENSG00000077782 via mapping the annotation from EntrezGene 2260 via mapping the annotation from GenAgeHuman 0169 annotated in GenAge release 17.

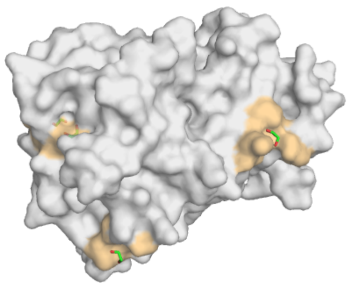

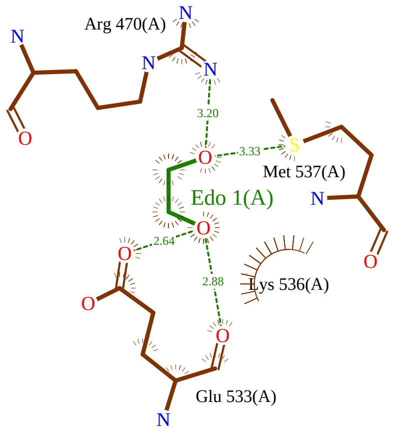

protein	amino acids contacts (binding site)
PDB:3rhx:chainB:P11362	E R E E M K M Q R A K A D F G H W W G G S Y P G V P H R M D W T F K E D R
PDB:3rhx:chainA:P11362	- R E E M K M Q R A K A D F G H W W G G S Y P G V P H R M D W T F K E D R
PDB:4f63:chainA:P11362	- R E E M K M Q R A K A D F G H W W G G S Y P G V P H R M D W T F K E D R
PDB:4f64:chainA:P11362	- R E E M K M Q R A K A D F G H W W G G S Y P G V P H R M D W T F K E D R
PDB:4f65:chainA:P11362	- R E E M K M Q R A K A D F - H W W G G S Y P G V P H R M D W T F K E D R
tr:D3DSX2:D3DSX2_HUMAN	E R E E M K M Q R A K A D F G H W W G G S Y P G V P H R M D W T F K E D R
tr:E7EU09:E7EU09_HUMAN	E R E E M K M Q R A K A D F G H W W G G S Y P G V P H R M D W T F K E D R
tr:J3KNT4:J3KNT4_HUMAN	E R E E M K M Q R A K A D F G H W W G G S Y P G V P H R M D W T F K E D R
sp:P11362:FGFR1_HUMAN	E R E E M K M Q R A K A D F G H W W G G S Y P G V P H R M D W T F K E D R
tr:Q63827:Q63827_RAT	E R E E M K M Q R A K A D F G H W W G G S Y P G V P H R M D W T F K E D R
tr:F1LM54:F1LM54_RAT	E R E E M K M Q R A K A D F G H W W G G S Y P G V P H R M D W T F K E D R
sp:P16092:FGFR1_MOUSE	E R E E M K M Q R A K A D F G H W W G G S Y P G V P H R M D W T F K E D R
tr:Q8CIM9:Q8CIM9_MOUSE	E R E E M K M Q R A K A D F G H W W G G S Y P G V P H R M D W T F K E D R
tr:J3QN85:J3QN85_MOUSE	E R E E M K M Q R A K A D F G H W W G G S Y P G V P H R M D W T F K E D R
sp:Q09147:FGFR2_DROME	E N E E M K M K R A R A D F G S W W G D Q Y P H I S Q R M E W T F A E S G
sp:Q10656:EGL15_CAEEL	E V E E F K V R R A K S D F G V W W G G T Y P T I A Y R M E W S F K D Y W

protein	whole protein ident	simil	domain-based ident	simil	contact-based ident	simil
PDB:3rhx:chainB:P11362	1.0	1.0	1.0	1.0	1.0	1.0
PDB:3rhx:chainA:P11362	1.0	1.0	1.0	1.0	0.97	0.94
PDB:4f63:chainA:P11362	1.0	1.0	1.0	1.0	0.97	0.94
PDB:4f64:chainA:P11362	1.0	1.0	1.0	1.0	0.97	0.94
PDB:4f65:chainA:P11362	1.0	1.0	1.0	1.0	0.95	0.89
tr:D3DSX2:D3DSX2_HUMAN	0.47	0.47	0.85	0.85	1.0	1.0
tr:E7EU09:E7EU09_HUMAN	0.85	0.87	1.0	1.0	1.0	1.0
tr:J3KNT4:J3KNT4_HUMAN	0.96	0.97	1.0	1.0	1.0	1.0
sp:P11362:FGFR1_HUMAN	1.0	1.0	1.0	1.0	1.0	1.0
tr:Q63827:Q63827_RAT	0.88	0.88	0.99	1.0	1.0	1.0
tr:F1LM54:F1LM54_RAT	0.98	1.0	1.0	1.0	1.0	1.0
sp:P16092:FGFR1_MOUSE	0.98	1.0	0.99	1.0	1.0	1.0
tr:Q8CIM9:Q8CIM9_MOUSE	0.98	0.99	0.99	1.0	1.0	1.0
tr:J3QN85:J3QN85_MOUSE	0.97	0.98	0.99	1.0	1.0	1.0
sp:Q09147:FGFR2_DROME	0.25	0.54	0.41	0.75	0.62	0.75
sp:Q10656:EGL15_CAEEL	0.25	0.57	0.36	0.72	0.57	0.73

btl (FBgn0005592) associated phenotypes
cell migration defective, neuroanatomy defective, somatic clone

(Information from FlyBase)

btl (UniProt:Q09147) annotation
Function: May be required for patterning of muscle precursor cells: generation of mesodermal and endodermal layers, invaginations of various types of cells, and CNS formation. Essential for the ability of the migrating tracheal and midline cells to recognize external guiding cues. (PubMed:1325393, PubMed:1849109, PubMed:8330538).
Subcellular location: Membrane; Single-pass type I membrane protein.
Tissue specificity: During embryogenesis, expression is seen in mesoderm, endodermal precursor cells, CNS midline cells and trachea and salivary duct ectodermal cells. (PubMed:1325393, PubMed:1849109, PubMed:8330538).
Developmental stage: All stages of development. (PubMed:1849109).

(Information from UniProt)

egl-15 (WBGene00001184) associated phenotypes
L1 arrest, SM migration variant, axon midline crossing variant, bag of worms, bloated, egg laying defective, egg laying imipramine resistant, egg laying levamisole resistant, egg laying phentolamine resistant, egg laying serotonin resistant, egg laying variant, hermaphrodite sex muscle development variant, hermaphrodite sex muscle morphology variant, hermaphrodite sexual development variant, imipramine resistant, larval arrest, larval lethal, scrawny, serotonin resistant, ventral nerve cord maintenance variant

(Information from WormBase)

egl-15 (UniProt:Q10656) annotation
Function: Activation leads to the phosphorylation of SOC-1 on Tyr- 408 triggering PTP-2 recruitment and/or activation. Activates the SEM-5/SOS-1/RAS/MAPK pathway (PubMed:11689700). Acts in the hypodermis to regulate axon growth (PubMed:12835392). Activates protein degradation in muscles (PubMed:14517244). Plays a role in fluid homeostasis by acting in the hypodermis and also has a role in larval development (PubMed:7585964). Isoform b has no effect on axon growth but affects the maintenance of axon position. Isoform b interaction with egl-17 is required for the guidance of sex myoblast migration during gonad development (PubMed:12835392). Isoforms a, c, d and e interaction with let-756 appears to play a role in maintaining body morphology at higher temperatures (PubMed:12835392). (PubMed:11689700, PubMed:12835392, PubMed:14517244, PubMed:7585964).
Cofactor: Mn(2+)
Subcellular location: Membrane; Single-pass type I membrane protein.
Developmental stage: Expressed in the hypodermal membrane during the larval stage. (PubMed:11689700).

Ptm: Activity is regulated by the phosphatase clr-1, however it is not known whether clr-1 acts directly on egl-15. (PubMed:9585503).
Disruption phenotype: Early arrest in larval development. Impaired guided migration of sex myoblasts. (PubMed:7585964).

(Information from UniProt)

Beta-enolase

Best gene implication in ageing for this target family came from gene Q27527 via mapping the annotation from WormBase WBGene00011884 annotated in WormBase 2014-03-11. Annotation GO 8340 (determination of adult lifespan) was Inferred from Mutant Phenotype

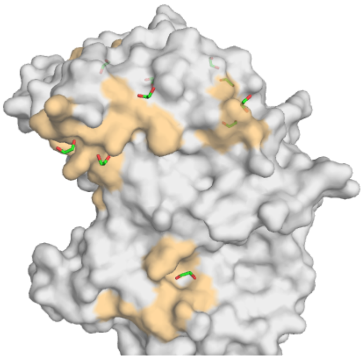

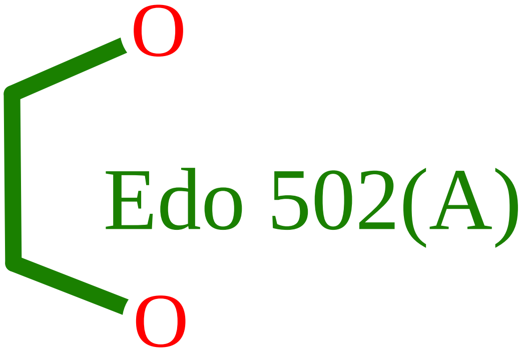

protein	amino acids contacts (binding site)
PDB:2xsx:chainA:P13929	R E E R A A G E R I H K G K D A T N V D E N N Y G E G F T S F S G V N K N G E
sp:P13929:ENOB_HUMAN	R E E R A A G E R I H K G K D A T N V D E N N Y G E G F T S F S G V N K N G E
sp:P15429:ENOB_RAT	R E E R A A G E R I H K G K D A T N V D E N N Y G E G F T S F S G V D K N G E
sp:P21550:ENOB_MOUSE	R E E R A A G E R I H K G K D A T N V D E N N Y G E G F T S F S G V D K N G E
tr:E1JHR5:E1JHR5_DROME	R Q E R A A G E K M H K N L D A T A V D S N K Y A D A F A N L G C T D K N G E
sp:P15007:ENO_DROME	R Q E R A A G E K M H K N L D A T A V D S N K Y A D A F A N L G C T D K N G E
sp:Q27527:ENO_CAEEL	R Q E R A A G E R M H K A L D A T A V D D N K Y G E T F N K F G A T S K N G E
sp:P42222:ERR3_YEAST	R T E R I A G E R M H K I P S A G N V D T A E Y P A A Y S A F K T V N K N G E
sp:P0CX10:ERR1_YEAST	R T E R I A G E R M H K I P S A G N V D T A E Y P A A Y S A F K T V N K N G E
sp:P0CX11:ERR2_YEAST	R T E R I A G E R M H K I P S A G N V D T A E Y P A A Y S A F K T V N K N G E

	whole protein		domain-based		contact-based	
protein	ident	simil	ident	simil	ident	simil
PDB:2xsx:chainA:P13929	1.0	1.0	1.0	1.0	1.0	1.0
sp:P13929:ENOB_HUMAN	1.0	1.0	1.0	1.0	1.0	1.0
sp:P15429:ENOB_RAT	0.98	1.0	0.98	1.0	0.97	0.99
sp:P21550:ENOB_MOUSE	0.98	0.99	0.98	0.99	0.97	0.99
tr:E1JHR5:E1JHR5_DROME	0.72	0.91	0.73	0.91	0.55	0.74
sp:P15007:ENO_DROME	0.63	0.79	0.73	0.91	0.55	0.74
sp:Q27527:ENO_CAEEL	0.73	0.91	0.73	0.91	0.65	0.78
sp:P42222:ERR3_YEAST	0.59	0.86	0.59	0.86	0.55	0.69
sp:P0CX10:ERR1_YEAST	0.59	0.86	0.59	0.87	0.55	0.69
sp:P0CX11:ERR2_YEAST	0.59	0.86	0.59	0.87	0.55	0.69

Eno (UniProt:P15007) annotation
Cofactor: Mg(2+)Note=Mg(2+) is required for catalysis and for stabilizing the dimer.;
Pathway: Carbohydrate degradation; glycolysis; pyruvate from D- glyceraldehyde 3-phosphate: step 4/5.
Subunit: Homodimer.
Subcellular location: Cytoplasm.

(Information from UniProt)

enol-1 (UniProt:Q27527) annotation
Cofactor: Mg(2+)Note=Mg(2+) is required for catalysis and for stabilizing the dimer. ;
Pathway: Carbohydrate degradation; glycolysis; pyruvate from D- glyceraldehyde 3-phosphate: step 4/5.
Subunit: Homodimer.
Subcellular location: Cytoplasm

(Information from UniProt)

Macrophage migration inhibitory factor

Best gene implication in ageing for this target family came from gene P34884 annotated in UniProt release 2014_02. Annotation GO subterm of 7568 (aging) was Inferred from Mutant Phenotype

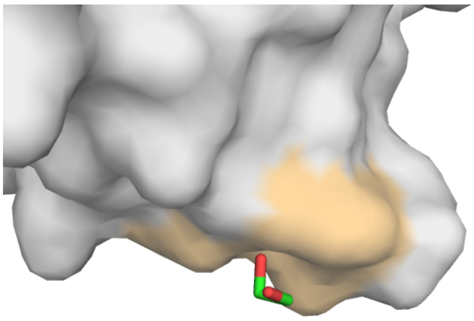

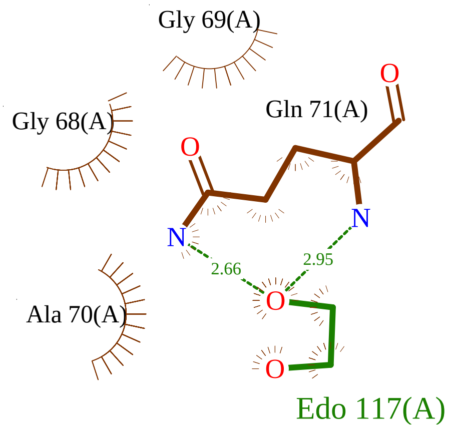

protein	amino acids contacts (binding site)			
PDB:3u18:chainA:P14174	G	G	A	Q
sp:P14174:MIF_HUMAN	G	G	A	Q
tr:I4AY87:I4AY87_HUMAN	G	G	A	Q
sp:P30904:MIF_RAT	G	G	A	Q
tr:D3ZQU2:D3ZQU2_RAT	G	G	A	Q
tr:D3ZXK6:D3ZXK6_RAT	G	G	A	Q
sp:P34884:MIF_MOUSE	G	G	A	Q
tr:Q545F0:Q545F0_MOUSE	G	G	A	Q
tr:Q9U228:Q9U228_CAEEL	G	S	A	K

	whole protein		domain-based		contact-based	
protein	ident	simil	ident	simil	ident	simil
PDB:3u18:chainA:P14174	1.0	1.0	1.0	1.0	1.0	1.0
sp:P14174:MIF_HUMAN	1.0	1.0	1.0	1.0	1.0	1.0
tr:I4AY87:I4AY87_HUMAN	1.0	1.0	1.0	1.0	1.0	1.0
sp:P30904:MIF_RAT	0.9	0.98	0.9	0.98	1.0	1.0
tr:D3ZQU2:D3ZQU2_RAT	0.7	0.87	0.7	0.87	1.0	1.0
tr:D3ZXK6:D3ZXK6_RAT	0.77	0.89	0.77	0.89	1.0	1.0
sp:P34884:MIF_MOUSE	0.9	0.97	0.89	0.97	1.0	1.0
tr:Q545F0:Q545F0_MOUSE	0.9	0.97	0.89	0.97	1.0	1.0
tr:Q9U228:Q9U228_CAEEL	0.33	0.78	0.33	0.78	0.5	0.75

Matrix metalloproteinase-9

Best gene implication in ageing for this target family came from gene D3ZYK8 via mapping the annotation from RGD 621320 annotated in RGD 2014-03-11. Annotation GO 7568 (aging) was

Inferred from Expression Pattern

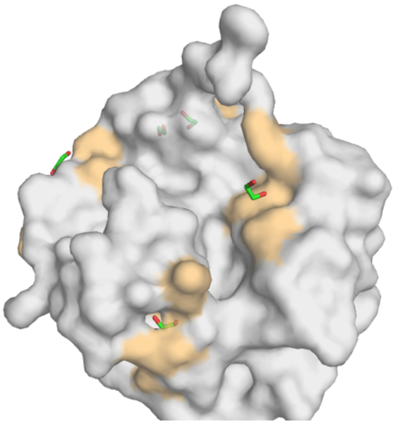

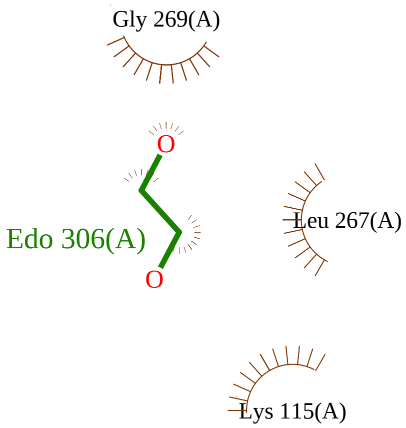

protein	amino acids contacts (binding site)
PDB:4jij:chainA:P14780	K W H H Y L H G P A L M R T R H L Y G
PDB:4jqg:chainA:P14780	K W H H Y L H G P A L M R T R H L Y G
sp:P14780:MMP9_HUMAN	K W H H Y L H G P A L M R T R H L Y G
tr:D3ZYK8:D3ZYK8_RAT	K W H H Y L H G P A L M H H Q H L Y G
sp:P41245:MMP9_MOUSE	K W D H Y L H G A A L L S L Q Y L Y G
tr:Q95X57:Q95X57_CAEEL	K W A H H V H G Y A L Y K V I W N Y G
tr:O44836:O44836_CAEEL	K W A H H V H G Y A L Y K V I W N Y G
tr:G5EBU3:G5EBU3_CAEEL	K W S K W V H E S A I F Q T Q A I Y G

	whole protein		domain-based		contact-based	
protein	ident	simil	ident	simil	ident	simil
PDB:4jij:chainA:P14780	0.99	0.99	0.99	0.99	1.0	1.0
PDB:4jqg:chainA:P14780	0.99	0.99	0.99	0.99	1.0	1.0
sp:P14780:MMP9_HUMAN	1.0	1.0	1.0	1.0	1.0	1.0
tr:D3ZYK8:D3ZYK8_RAT	0.75	0.92	0.84	0.95	0.84	0.94
sp:P41245:MMP9_MOUSE	0.72	0.89	0.82	0.94	0.63	0.77
tr:Q95X57:Q95X57_CAEEL	0.12	0.34	0.25	0.57	0.47	0.6
tr:O44836:O44836_CAEEL	0.13	0.38	0.27	0.6	0.47	0.6
tr:G5EBU3:G5EBU3_CAEEL	0.19	0.46	0.33	0.67	0.37	0.72

WBGene00019212 associated phenotypes
lethal, sterile

(Information from WormBase)

Tyrosine-protein phosphatase non-receptor type 1

Best gene implication in ageing for this target family came from gene P18031 via mapping the annotation from Ensembl ENSG00000196396 via mapping the annotation from EntrezGene 5770 via mapping the annotation from GenAgeHuman 0033 annotated in GenAge release 17.

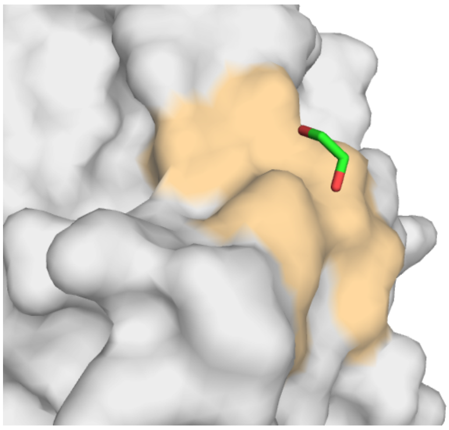

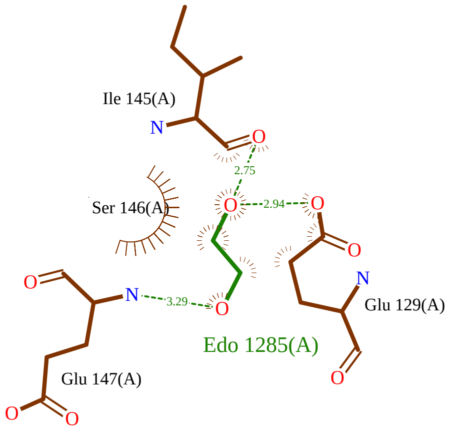

protein	amino acids contacts (binding site)
PDB:4bjo:chainA:P18031	E I S E
tr:B4DSN5:B4DSN5_HUMAN	E I S E
sp:P18031:PTN1_HUMAN	E I S E
tr:A8K3M3:A8K3M3_HUMAN	E I S E
sp:P20417:PTN1_RAT	E I S E
tr:Q3T9Y9:Q3T9Y9_MOUSE	E I S E
tr:Q3TB93:Q3TB93_MOUSE	E I S E
tr:Q3TZW9:Q3TZW9_MOUSE	E I S E
sp:P35821:PTN1_MOUSE	E I S E
tr:B8A420:B8A420_DROME	A V R L
tr:Q8IRH4:Q8IRH4_DROME	A V R L
sp:Q9W0G1:PTP61_DROME	A V R L
tr:D3DMI1:D3DMI1_DROME	A V R L
tr:I0B1P6:I0B1P6_DROME	A V R L

	whole protein		domain-based		contact-based	
protein	ident	simil	ident	simil	ident	simil
PDB:4bjo:chainA:P18031	0.98	0.98	1.0	1.0	1.0	1.0
tr:B4DSN5:B4DSN5_HUMAN	0.83	0.83	0.76	0.76	1.0	1.0
sp:P18031:PTN1_HUMAN	1.0	1.0	1.0	1.0	1.0	1.0
tr:A8K3M3:A8K3M3_HUMAN	1.0	1.0	1.0	1.0	1.0	1.0
sp:P20417:PTN1_RAT	0.84	0.94	0.96	0.99	1.0	1.0
tr:Q3T9Y9:Q3T9Y9_MOUSE	0.54	0.56	0.72	0.75	1.0	1.0
tr:Q3TB93:Q3TB93_MOUSE	0.7	0.73	0.95	0.99	1.0	1.0
tr:Q3TZW9:Q3TZW9_MOUSE	0.83	0.94	0.95	0.99	1.0	1.0
sp:P35821:PTN1_MOUSE	0.83	0.94	0.95	0.99	1.0	1.0
tr:B8A420:B8A420_DROME	0.23	0.46	0.35	0.55	0.0	0.42
tr:Q8IRH4:Q8IRH4_DROME	0.23	0.46	0.35	0.55	0.0	0.42
sp:Q9W0G1:PTP61_DROME	0.3	0.59	0.49	0.8	0.0	0.42
tr:D3DMI1:D3DMI1_DROME	0.3	0.59	0.49	0.8	0.0	0.42
tr:I0B1P6:I0B1P6_DROME	0.3	0.59	0.49	0.8	0.0	0.42

Ptp61F (UniProt:Q9W0G1) annotation

Function: Non-receptor protein tyrosine phosphatase required for maintaining Dock in its non-phosphorylated state. (PubMed:12014990, PubMed:8463208).

Subcellular location: Isoform A: Cytoplasm. Endomembrane system. Note=Associates with the membranes of the reticular network and the mitochondria.

Subcellular location: Isoform B: Nucleus.

Tissue specificity: Expressed during oogenesis and embryogenesis. Isoform A and isoform B are expressed in distinct patterns. Isoform B is expressed in the mesoderm and neuroblast layer during germband extension and later in the gut epithelia. Isoform A accumulates in 16 segmentally repeated stripes in the ectoderm during germband extension. These stripes are flanked by, and adjacent to, the domains of engrailed and wingless gene expression in the anterior/posterior axis. In stage 10 embryos, isoform A colocalizes with the area lateral to the denticle belts that will give rise to naked cuticle. Isoform A is also expressed later in embryogenesis in the central nervous system.

(PubMed:9256342).

(Information from UniProt)

Transcription initiation factor TFIID subunit 1

Best gene implication in ageing for this target family came from gene P21675 via mapping the annotation from Ensembl ENSG00000147133 via mapping the annotation from EntrezGene 6872 via mapping the annotation from GenAgeHuman 0180 annotated in GenAge release 17.

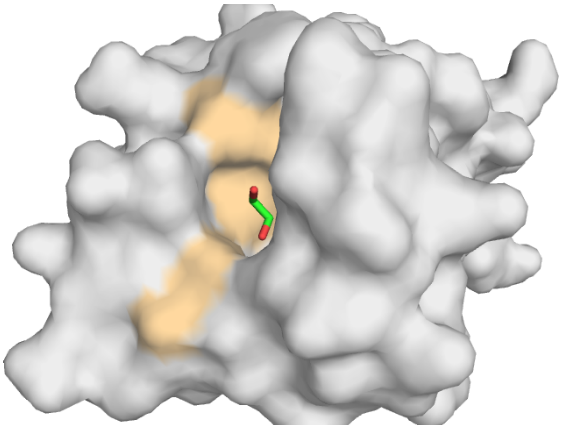

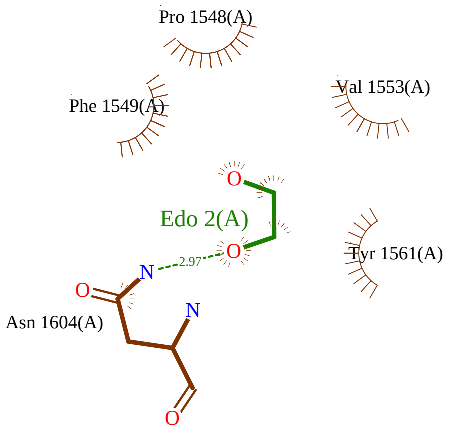

protein	amino acids contacts (binding site)
PDB:3uv4:chainA:P21675	P F V Y N
sp:P21675:TAF1_HUMAN	P F V Y N
tr:D3ZJI8:D3ZJI8_RAT	P F V Y N
tr:S4R1B9:S4R1B9_MOUSE	P F V Y N
tr:B1Q2W7:B1Q2W7_MOUSE	P F V Y N
sp:Q80UV9:TAF1_MOUSE	P F V Y N
tr:D3YZK4:D3YZK4_MOUSE	P F V Y N
tr:Q29R44:Q29R44_DROME	P F V Y N
tr:E1JJ72:E1JJ72_DROME	P F V Y N
sp:P51123:TAF1_DROME	P F V Y N
tr:G5EGM3:G5EGM3_CAEEL	L F V Y N
sp:P46677:TAF1_YEAST	- - - - -

	whole protein		domain-based		contact-based	
protein	ident	simil	ident	simil	ident	simil
PDB:3uv4:chainA:P21675	1.0	1.0	1.0	1.0	1.0	1.0
sp:P21675:TAF1_HUMAN	1.0	1.0	1.0	1.0	1.0	1.0
tr:D3ZJI8:D3ZJI8_RAT	0.93	0.97	0.99	1.0	1.0	1.0
tr:S4R1B9:S4R1B9_MOUSE	0.85	0.88	0.98	0.99	1.0	1.0
tr:B1Q2W7:B1Q2W7_MOUSE	0.95	0.97	0.98	0.99	1.0	1.0
sp:Q80UV9:TAF1_MOUSE	0.95	0.98	0.98	0.99	1.0	1.0
tr:D3YZK4:D3YZK4_MOUSE	0.94	0.97	0.98	0.99	1.0	1.0
tr:Q29R44:Q29R44_DROME	0.44	0.71	0.47	0.81	1.0	1.0
tr:E1JJ72:E1JJ72_DROME	0.42	0.68	0.47	0.81	1.0	1.0
sp:P51123:TAF1_DROME	0.42	0.68	0.47	0.81	1.0	1.0
tr:G5EGM3:G5EGM3_CAEEL	0.3	0.59	0.35	0.77	0.8	0.77
sp:P46677:TAF1_YEAST	0.15	0.36	0.0	0.0	0.0	0.0

Taf1 (FBgn0010355) associated phenotypes

cell autonomous, neuroanatomy defective, partially lethal - majority die, some die during pupal stage

(Information from FlyBase)

Taf1 (UniProt:P51123) annotation

Function: TFIID is a multimeric protein complex that plays a central role in mediating promoter responses to various activators and repressors. Largest component and core scaffold of the complex. Contains N- and C-terminal Ser/Thr kinase domains which can autophosphorylate or transphosphorylate other transcription factors. Possesses DNA-binding activity. Essential for progression of the G1 phase of the cell cycle. Negative regulator of the TATA box-binding activity of Tbp. (PubMed:8504928, PubMed:8625415, PubMed:9741622).

Cofactor:Mg(2+)

Enzyme regulation: Autophosphorylates on Ser residues.

Subunit: Belongs to the TFIID complex which is composed of TATA binding protein (Tbp) and a number of TBP-associated factors (Tafs). Taf1 is the largest component of the TFIID complex. Interacts with Tbp, Taf2, Taf4 and Taf6. (PubMed:8178153, PubMed:8262073, PubMed:8327460, PubMed:8464492).

Subcellular location: Nucleus.

Caution: The C-terminal Ser/Thr kinase domain was reported tophosphorylate histone H2B at 'Ser-33' (PubMed:15143281). However, the paper was retracted because some data, results and conclusions in the paper are not reliable. (PubMed:15143281).

(Information from UniProt)

taf-1 (WBGene00006382) associated phenotypes

lethal, sterile

(Information from WormBase)

Nitric oxide synthase, inducible

Best gene implication in ageing for this target family came from gene M0RDG2 via mapping the annotation from RGD 3185 annotated in RGD 2014-03-11. Annotation GO 7568 (aging) was Inferred from Expression Pattern

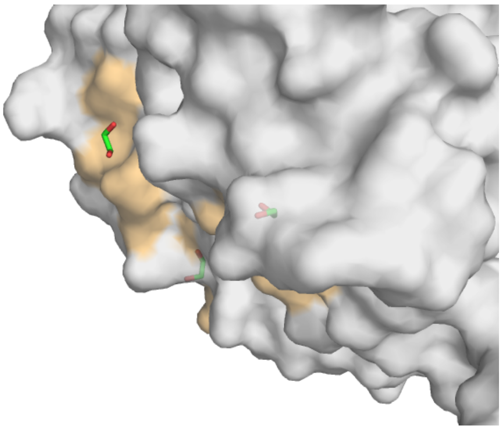

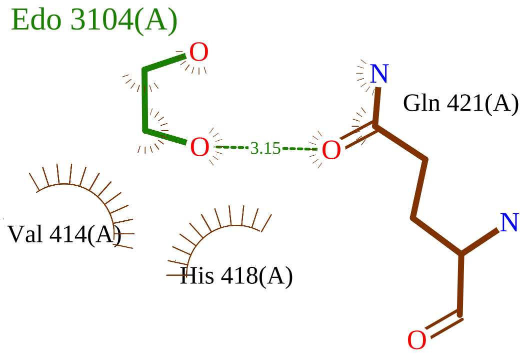

protein	amino acids contacts (binding site)
PDB:1jwk:chainA:P29477	Y R K I H K N P K Q D I F K M G I Q W N L W Y V L H Q Q V M S N R V L F Y
PDB:1m9t:chainA:P29477	Y R K I H K N P K Q D I F K M G I Q W N L W Y V L H Q Q V M S N R V L F Y
PDB:2orr:chainA:P29477	- - - - - - N P K Q D I F K M G I Q W N L W Y V L H Q Q V M S N - V L F Y
PDB:2ors:chainA:P29477	- - - - - - N P K Q D I F K M G I Q W N L W Y V L H Q Q V M S N - V L F Y
PDB:3e68:chainA:P29477	Y R K I H K N P K Q D I F K M G I Q W N L W Y V L H Q Q V M S N R V L F Y
PDB:3e7m:chainA:P29477	Y R K I H K N P K Q D I F K M G I Q W N L W Y V L H Q Q V M S N R V L F Y
sp:P35228:NOS2_HUMAN	H R K T Q K T P K Q D I F K Q G I Q W N L W Y I L H Q Q V M S N R V L F Y
tr:F1LSH9:F1LSH9_RAT	H R K I H K N S K Q D I F K M G I Q W S L W Y A L H Q Q V M S N R V L F Y
tr:MORDG2:MORDG2_RAT	H R K I H K N S K Q D I F K M G I Q W S L W Y A L H Q Q V M S N R V L F Y
sp:P29477:NOS2_MOUSE	Y R K I H K N P K Q D I F K M G I Q W N L W Y V L H Q Q V M S N R V L F Y
sp:Q27571:NOS_DROME	P R K E Y K N I G Q T I Y K L G I Q W K L W Y I L H Q R V V S N R Y L S E
sp:P16603:NCPR_YEAST	- - - - - - - - - A - - - - - - - - - D - - - - - - - - - - - - - - - - -

	whole protein		domain-based		contact-based	
protein	ident	simil	ident	simil	ident	simil
PDB:1jwk:chainA:P29477	1.0	1.0	1.0	1.0	1.0	1.0
PDB:1m9t:chainA:P29477	1.0	1.0	1.0	1.0	1.0	1.0
PDB:2orr:chainA:P29477	1.0	1.0	1.0	1.0	0.81	0.61
PDB:2ors:chainA:P29477	1.0	1.0	1.0	1.0	0.81	0.61
PDB:3e68:chainA:P29477	1.0	1.0	1.0	1.0	1.0	1.0
PDB:3e7m:chainA:P29477	1.0	1.0	1.0	1.0	1.0	1.0
sp:P35228:NOS2_HUMAN	0.8	0.93	0.82	0.94	0.84	0.9
tr:F1LSH9:F1LSH9_RAT	0.91	0.95	0.95	0.98	0.89	0.93
tr:MORDG2:MORDG2_RAT	0.92	0.95	0.95	0.98	0.89	0.93
sp:P29477:NOS2_MOUSE	1.0	1.0	1.0	1.0	1.0	1.0
sp:Q27571:NOS_DROME	0.37	0.68	0.49	0.81	0.59	0.69
sp:P16603:NCPR_YEAST	0.14	0.38	0.02	0.07	0.0	0.0

Nos (FBgn0011676) associated phenotypes

chemical resistant, developmental rate defective, dominant, eclosion defective, increased cell size, large body, neuroanatomy defective, partially lethal - majority die, small body, some die during pupal stage

(Information from FlyBase)

Nos (UniProt:Q27571) annotation

Function: Produces nitric oxide (NO) which is a messenger molecule with diverse functions throughout the body. Truncated isoforms (isoform 3-isoform 6) are able to form intracellular complexes with the full length protein and serve as dominant negative inhibitors of the enzyme activity. (PubMed:11526108, PubMed:12804606, PubMed:7568075).
Cofactor: heme
Cofactor: FADNote=Binds 1 FAD. ;
Cofactor: FMNNote=Binds 1 FMN. ;
Enzyme regulation: Stimulated by calcium/calmodulin. (PubMed:7568075).
Developmental stage: Isoform 3 is expressed in larvae only. Isoform 4, isoform 5, isoform 6 and isoform 10 are expressed throughout development from embryos to adults. (PubMed:11526108).

(Information from UniProt)

RAC-beta serine/threonine-protein kinase

Best gene implication in ageing for this target family came from gene Q9XTG7 annotated in UniProt release 2014_02. Annotation GO 8340 (determination of adult lifespan) was Inferred from Mutant Phenotype

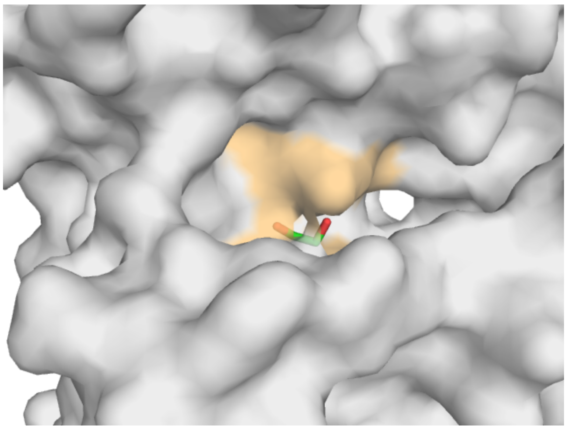

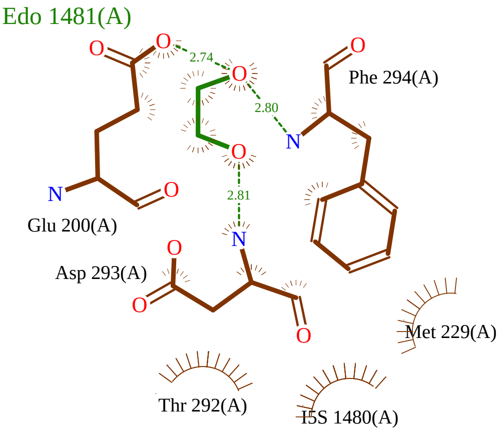

protein	amino acids contacts (binding site)				
PDB:2jdo:chainA:P31751	E	M	T	D	F
tr:B3KP61:B3KP61_HUMAN	E	M	T	D	F
tr:B7Z5R1:B7Z5R1_HUMAN	E	M	T	D	F
tr:B4DG79:B4DG79_HUMAN	E	M	T	D	F
tr:M0R0P9:M0R0P9_HUMAN	E	M	T	D	F
sp:Q9Y243:AKT3_HUMAN	E	M	T	D	F
sp:P31749:AKT1_HUMAN	E	M	T	D	F
tr:B0LPE5:B0LPE5_HUMAN	E	M	T	D	F
sp:P31751:AKT2_HUMAN	E	M	T	D	F
tr:F1M6A8:F1M6A8_RAT	E	M	T	D	F
sp:P47196:AKT1_RAT	E	M	T	D	F
tr:Q3HSE5:Q3HSE5_RAT	E	M	T	D	F
tr:Q8CE74:Q8CE74_MOUSE	E	M	T	D	F
sp:Q9WUA6:AKT3_MOUSE	E	M	T	D	F
sp:Q60823:AKT2_MOUSE	E	M	T	D	F
sp:P31750:AKT1_MOUSE	E	M	T	D	F
tr:Q3TY95:Q3TY95_MOUSE	E	M	T	D	F
tr:H1ZY64:H1ZY64_DROME	E	M	A	D	F
sp:Q8INB9:AKT1_DROME	E	M	A	D	F
tr:H1ZY63:H1ZY63_DROME	E	M	A	D	F
sp:Q9XTG7:AKT2_CAEEL	E	M	T	D	F
sp:P11792:SCH9_YEAST	E	T	C	D	F

protein	whole protein ident	simil	domain-based ident	simil	contact-based ident	simil
PDB:2jdo:chainA:P31751	0.97	0.99	1.0	1.0	1.0	1.0
tr:B3KP61:B3KP61_HUMAN	0.79	0.79	1.0	1.0	1.0	1.0
tr:B7Z5R1:B7Z5R1_HUMAN	0.71	0.82	0.88	0.97	1.0	1.0
tr:B4DG79:B4DG79_HUMAN	0.87	0.87	1.0	1.0	1.0	1.0
tr:M0R0P9:M0R0P9_HUMAN	0.78	0.8	1.0	1.0	1.0	1.0
sp:Q9Y243:AKT3_HUMAN	0.76	0.92	0.85	0.95	1.0	1.0
sp:P31749:AKT1_HUMAN	0.81	0.94	0.88	0.97	1.0	1.0
tr:B0LPE5:B0LPE5_HUMAN	0.81	0.94	0.88	0.97	1.0	1.0
sp:P31751:AKT2_HUMAN	1.0	1.0	1.0	1.0	1.0	1.0
tr:F1M6A8:F1M6A8_RAT	0.76	0.92	0.85	0.95	1.0	1.0
sp:P47196:AKT1_RAT	0.81	0.94	0.88	0.97	1.0	1.0
tr:Q3HSE5:Q3HSE5_RAT	0.98	0.99	0.99	1.0	1.0	1.0
tr:Q8CE74:Q8CE74_MOUSE	0.72	0.73	0.99	1.0	1.0	1.0
sp:Q9WUA6:AKT3_MOUSE	0.76	0.92	0.85	0.95	1.0	1.0
sp:Q60823:AKT2_MOUSE	0.98	0.99	0.99	1.0	1.0	1.0
sp:P31750:AKT1_MOUSE	0.81	0.94	0.88	0.97	1.0	1.0
tr:Q3TY95:Q3TY95_MOUSE	0.98	0.99	0.99	1.0	1.0	1.0
tr:H1ZY64:H1ZY64_DROME	0.48	0.68	0.73	0.92	0.8	0.87
sp:Q8INB9:AKT1_DROME	0.48	0.68	0.73	0.92	0.8	0.87
tr:H1ZY63:H1ZY63_DROME	0.48	0.68	0.73	0.92	0.8	0.87
sp:Q9XTG7:AKT2_CAEEL	0.48	0.75	0.62	0.87	1.0	1.0
sp:P11792:SCH9_YEAST	0.2	0.42	0.46	0.82	0.6	0.47

Akt1 (FBgn0010379) associated phenotypes

RU486 conditional, body size defective, cell death defective, cell size defective, chemical resistant, chemical sensitive, circadian rhythm defective, decreased cell death, decreased cell number, decreased cell size, developmental rate defective, germline clone, heat sensitive, hyperplasia, increased cell death, increased cell size, lethal - all die before end of pupal stage, long lived, maternal effect, neuroanatomy defective, neurophysiology defective, nutrition conditional, oxidative stress response defective, partially lethal - majority die, planar polarity defective, polyphasic, short lived, size defective, small body, somatic clone, some die during pupal stage

(Information from FlyBase)

Akt1 (UniProt:Q8INB9) annotation

Function: Serine/threonine kinase involved in various developmental processes. During early embryogenesis, acts as a survival protein. During mid-embryogenesis, phosphorylates and activates trh, a transcription factor required for tracheal cell fate determination. Also regulates tracheal cell migration. Later in development, acts downstream of PI3K and Pk61C/PDK1 in the insulin receptor transduction pathway which regulates cell growth and organ size, by phosphorylating and antagonizing FOXO transcription factor. Controls follicle cell size during oogenesis. May also stimulate cell growth by phosphorylating Gig/Tsc2 and inactivating the Tsc complex. Dephosphorylation of 'Ser-586' by Phlpp triggers apoptosis and suppression of tumor growth. (PubMed:10587646, PubMed:10962553, PubMed:11740943, PubMed:11752451, PubMed:11872800, PubMed:12172554, PubMed:12893776, PubMed:14525946, PubMed:15466161, PubMed:15712201, PubMed:9601646).
Subcellular location: Cytoplasm, cytosol. Cell membrane. Note=Recruited to plasma membrane upon activation.
Tissue specificity: Ubiquitously expressed. Present in ovary, where it is concentrated at the basal side of follicle cells. (PubMed:7876156).
Developmental stage: Expressed both maternally and zygotically. Strongly expressed in embryo and pupa. Weakly expressed in larva. Mildly expressed in adult. (PubMed:15712201, PubMed:7876156).
Domain: Binding of the PH domain to the phosphatidylinositol 3- kinase alpha (PI(3)K) results in its targeting to the plasma membrane.
Ptm: Phosphorylated and activated by Pk61C/PDK1. Phosphorylated on Ser-586 by the rictor-Tor complex. (PubMed:10962553, PubMed:11344272, PubMed:15718470, PubMed:18327897).
Disruption phenotype: Death at the first instar larval stage. (PubMed:9601646).

(Information from UniProt)

akt-2 (WBGene00000103) associated phenotypes
fat content increased, germ cell hypersensitive ionizing radiation, hypersensitivity to mutagen

(Information from WormBase)

akt-2 (UniProt:Q9XTG7) annotation
Function: Acts downstream of age-1 and pdk-1 in the daf-2/insulin receptor-like transduction pathway. Essential role in regulating developmental arrest at the dauer stage. Phosphorylates Forkhead-related daf-16 and the longevity-promoting skn-1 transcription factors, which inhibits their entry into the nucleus and antagonizes their functions. Role in immune function and pathogen resistance. (PubMed:10364160, PubMed:11381260, PubMed:11747825, PubMed:15068796, PubMed:18358814, PubMed:18782349, PubMed:9716402).
Cofactor:Mg(2+)
Enzyme regulation: Phosphorylated and activated by pdk-1. (PubMed:15068796).
Subunit: Interacts with pdk-1, sgk-1, akt-1 and daf-16. Part of a complex containing sgk-1, akt-1 and akt-2. (PubMed:15068796).
Tissue specificity: Expressed in neurons, muscle cells of the pharynx, rectal gland cells, and spermatheca. (PubMed:9716402).
Developmental stage: Expressed in late stage embryos and throughout life. (PubMed:9716402).
Disruption phenotype: Defective egg-laying and increased resistance to pathogens. Simultaneous knockdown of akt-1 and akt-2 result in dauer formation and a weak extension to life span. (PubMed:15068796, PubMed:18782349).

(Information from UniProt)

Pyrroline-5-carboxylate reductase 1, mitochondrial

Best gene implication in ageing for this target family came from gene P32322 via mapping the annotation from Ensembl ENSG00000183010 via mapping the annotation from EntrezGene 5831 via mapping the annotation from GenAgeHuman 0280 annotated in GenAge release 17.

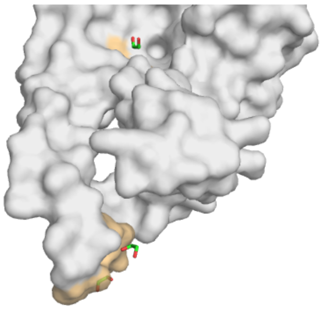

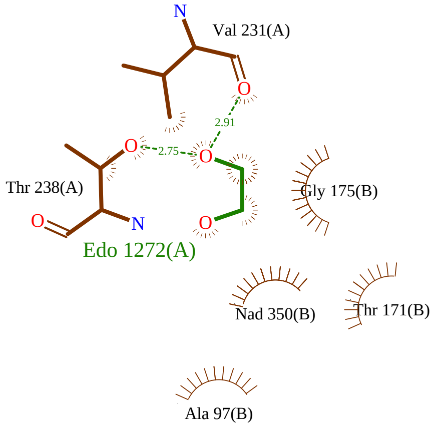

protein	amino acids contacts (binding site)
PDB:2izz:chainA:P32322	T G D N V S P T
tr:Q8TBX0:Q8TBX0_HUMAN	T G D N V S P T
tr:J3QKT4:J3QKT4_HUMAN	T G D N V S P T
tr:J3KQ22:J3KQ22_HUMAN	T G D N V S P T
tr:E2QRB3:E2QRB3_HUMAN	T G D N V S P T
sp:P32322:P5CR1_HUMAN	T G D N V S P T
tr:D3ZXI0:D3ZXI0_RAT	T G D N V S P T
tr:A2ABZ2:A2ABZ2_MOUSE	T G D N V S P T
tr:Q3UTR5:Q3UTR5_MOUSE	T G D N V S P T
sp:Q922W5:P5CR1_MOUSE	T G D N V S P T
tr:Q8IN96:Q8IN96_DROME	T G D G V S P T
tr:Q9VEJ3:Q9VEJ3_DROME	T G D G V S P T
tr:Q21544:Q21544_CAEEL	T G D D V S P S
sp:P32263:P5CR_YEAST	T G H Q V T P T

	whole protein		domain-based		contact-based	
protein	ident	simil	ident	simil	ident	simil
PDB:2izz:chainA:P32322	1.0	1.0	1.0	1.0	1.0	1.0
tr:Q8TBX0:Q8TBX0_HUMAN	0.54	0.54	1.0	1.0	1.0	1.0
tr:J3QKT4:J3QKT4_HUMAN	0.76	0.76	1.0	1.0	1.0	1.0
tr:J3KQ22:J3KQ22_HUMAN	0.79	0.79	0.84	0.84	1.0	1.0
tr:E2QRB3:E2QRB3_HUMAN	0.9	0.9	0.7	0.7	1.0	1.0
sp:P32322:P5CR1_HUMAN	1.0	1.0	1.0	1.0	1.0	1.0
tr:D3ZXI0:D3ZXI0_RAT	0.87	0.94	0.97	0.99	1.0	1.0
tr:A2ABZ2:A2ABZ2_MOUSE	0.7	0.73	0.66	0.68	1.0	1.0
tr:Q3UTR5:Q3UTR5_MOUSE	0.87	0.94	0.96	0.99	1.0	1.0
sp:Q922W5:P5CR1_MOUSE	0.87	0.94	0.96	0.99	1.0	1.0
tr:Q8IN96:Q8IN96_DROME	0.27	0.45	0.54	0.85	0.88	0.91
tr:Q9VEJ3:Q9VEJ3_DROME	0.38	0.69	0.54	0.85	0.88	0.91
tr:Q21544:Q21544_CAEEL	0.42	0.72	0.57	0.87	0.75	0.91
sp:P32263:P5CR_YEAST	0.25	0.58	0.38	0.78	0.63	0.78

CG5840 (UniProt:Q9VEJ3) annotation
Pathway: Amino-acid biosynthesis; L-proline biosynthesis; L- proline from L-glutamate 5-semialdehyde: step 1/1. (RuleBase:RU003903).

(Information from UniProt)

Catenin beta-1

Best gene implication in ageing for this target family came from gene P35222 via mapping the annotation from Ensembl ENSG00000168036 via mapping the annotation from EntrezGene 1499 via mapping the annotation from GenAgeHuman 0223 annotated in GenAge release 17.

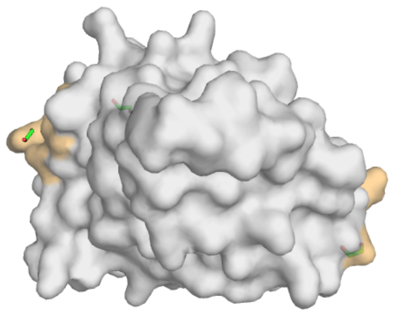

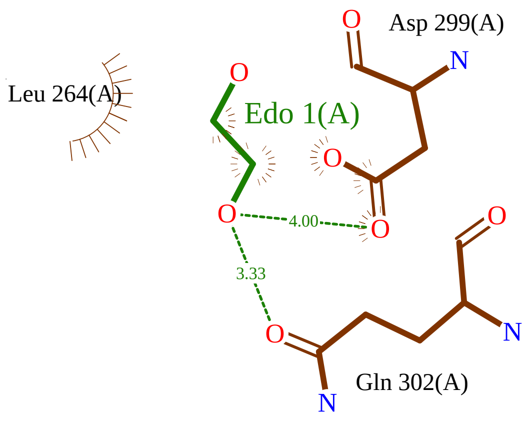

protein	amino acids contacts (binding site)
PDB:3sl9:chainA:P35222	E D Q L R L D Q
tr:B4DGU4:B4DGU4_HUMAN	E D Q L R L D Q
sp:P35222:CTNB1_HUMAN	E D Q L R L D Q
sp:Q9WU82:CTNB1_RAT	E D Q L R L D Q
tr:E9Q6A9:E9Q6A9_MOUSE	E D Q L R L D Q
sp:Q02248:CTNB1_MOUSE	E D Q L R L D Q
sp:P18824:ARM_DROME	E D Q L R L D Q

	whole protein		domain-based		contact-based	
protein	ident	simil	ident	simil	ident	simil
PDB:3sl9:chainA:P35222	1.0	1.0	1.0	1.0	1.0	1.0
tr:B4DGU4:B4DGU4_HUMAN	0.99	0.99	1.0	1.0	1.0	1.0
sp:P35222:CTNB1_HUMAN	1.0	1.0	1.0	1.0	1.0	1.0
sp:Q9WU82:CTNB1_RAT	1.0	1.0	1.0	1.0	1.0	1.0
tr:E9Q6A9:E9Q6A9_MOUSE	0.74	0.76	0.82	0.83	1.0	1.0
sp:Q02248:CTNB1_MOUSE	1.0	1.0	1.0	1.0	1.0	1.0
sp:P18824:ARM_DROME	0.65	0.83	0.79	0.94	1.0	1.0

arm (FBgn0000117) associated phenotypes

RU486 conditional, cell autonomous, cell migration defective, cell non-autonomous, cell polarity defective, cell shape defective, germline clone, heat sensitive, hyperplasia, increased cell death, lethal - all die before end of pupal stage, memory defective, mitotic cell cycle defective, neuroanatomy defective, partially lethal - majority die, planar polarity defective, segment polarity phenotype, size defective, somatic clone, some die during pupal stage

(Information from FlyBase)

arm (UniProt:P18824) annotation

Function: Isoform neural may associate with CadN and participate in the transmission of developmental information. Can associate with alpha-catenin. Isoform cytoplasmic accumulates through wg signaling; arm function in wg signal transduction is required early in development for determination of neuroblast fate. Arm and Abl proteins function cooperatively at adherens junctions in both the CNS and epidermis. (PubMed:9635189).
Subunit: Interacts with Mer and Moe at the adherens junction. Interacts with Inx2. (PubMed:15047872, PubMed:8666669).
Subcellular location: Cytoplasm ECO:0000269—PubMed:8666669. Cell membrane (PubMed:8666669); Peripheral membrane protein (PubMed:8666669); Cytoplasmic side (PubMed:8666669). Cell junction, adherens junction Note=Inner surface of cell membrane and adherens junction.
Tissue specificity: Isoform cytoplasmic accumulates at low levels in axons, at high levels in specific cells along the CNS midline and in leg and eye imaginal disks. Isoform neural accumulates in the axon tracts of the CNS. Both isoforms accumulate in the peripheral nervous system. (PubMed:9635189).
Developmental stage: Present at all stages, but reaches the highest levels during early to mid-embryogenesis. Isoform cytoplasmic is the predominant one from the cellular blastoderm stage until germ-band retraction. Isoform neural is first seen after germ band retraction.
Ptm: Phosphorylated on Ser, Thr and Tyr residues. Level of phosphorylation varies both during embryonic development and from embryonic tissue to tissue. Sgg is required for phosphorylation and wg signal negatively regulates arm phosphorylation. Hypophosphorylated form of arm increases in steady-state levels. (PubMed:18327897, PubMed:7529201).

(Information from UniProt)

Cystathionine beta-synthase

Best gene implication in ageing for this target family came from gene Q9VRD9 annotated in UniProt release 2014_02. Annotation GO 8340 (determination of adult lifespan) was Inferred from Mutant

Phenotype

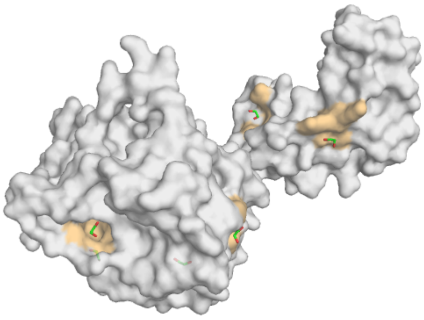

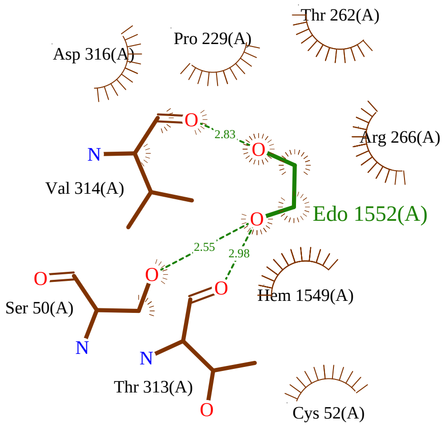

protein	amino acids contacts (binding site)
PDB:4coo:chainA:P35520	S C I E V A F S H P T R Y D F I T V L D R T W K Q F L L
sp:P35520:CBS_HUMAN	S C I E V A F S H P T R Y D F I T V L D R T W K Q F L L
sp:P32232:CBS_RAT	S C I E V A F S H P T R Y D F I T V L D R A W K Q F L L
sp:Q91WT9:CBS_MOUSE	S C I E V A F S H P T R Y D F I T V L D R A W K Q F L L
tr:Q9VRD9:Q9VRD9_DROME	S C I K I A Y G L P S R Y D F P T V F D D T W K R V V T
tr:Q9N4K2:Q9N4K2_CAEEL	K L L T V A F S H P T R Y D F I G T L D E K - - - - - -
sp:P32582:CBS_YEAST	- - I E I A W S H P S K Y D F V Q V L D R K F K K F L L
tr:E9P980:E9P980_YEASX	- - I E I A W S H P S K Y D F V Q V L D R K F K K F L L

protein	whole protein ident	whole protein simil	domain-based ident	domain-based simil	contact-based ident	contact-based simil
PDB:4coo:chainA:P35520	1.0	1.0	1.0	1.0	1.0	1.0
sp:P35520:CBS_HUMAN	1.0	1.0	1.0	1.0	1.0	1.0
sp:P32232:CBS_RAT	0.82	0.92	0.83	0.93	0.96	0.98
sp:Q91WT9:CBS_MOUSE	0.82	0.92	0.84	0.93	0.96	0.98
tr:Q9VRD9:Q9VRD9_DROME	0.47	0.77	0.5	0.81	0.54	0.75
tr:Q9N4K2:Q9N4K2_CAEEL	0.24	0.39	0.34	0.55	0.5	0.36
sp:P32582:CBS_YEAST	0.36	0.64	0.37	0.67	0.61	0.73
tr:E9P980:E9P980_YEASX	0.35	0.64	0.37	0.67	0.61	0.73

Cbs (FBgn0031148) associated phenotypes
RU486 conditional, long lived, short lived

(Information from FlyBase)

Cbs (UniProt:Q9VRD9) annotation
Cofactor: pyridoxal 5'-phosphate Evidence=(RuleBase:RU361204);

(Information from UniProt)

Phosphoenolpyruvate carboxykinase, cytosolic [GTP]

Best gene implication in ageing for this target family came from gene Q9Z2V4 via mapping the annotation from Ensembl ENSMUSG00000027513 via mapping the annotation from EntrezGene 18534 via mapping the annotation from GenAgeModels 0497 annotated in GenAge release 17.

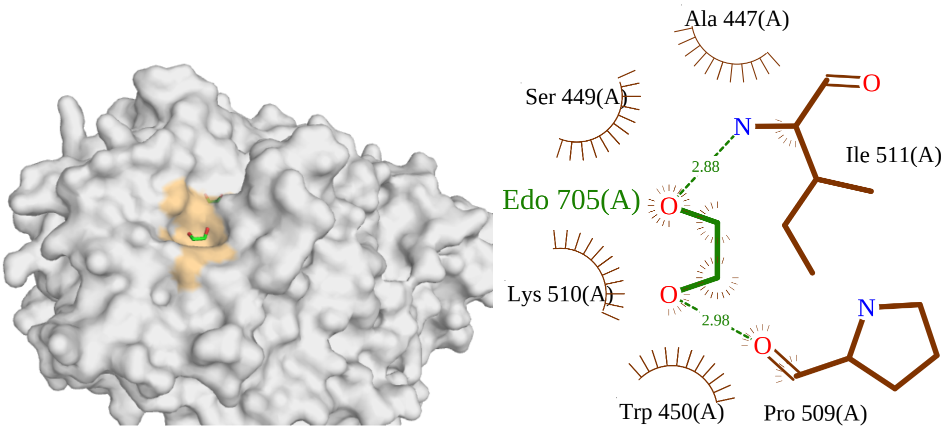

protein	amino acids contacts (binding site)												
PDB:1khb:chainA:P35558	Y	I	I	Q	V	I	E	A	S	W	P	K	I
PDB:1khf:chainA:P35558	Y	I	I	Q	V	I	E	A	S	W	P	K	I
PDB:1khg:chainA:P35558	Y	I	I	Q	V	I	E	A	S	W	P	K	I
PDB:1m51:chainA:P35558	Y	I	I	Q	V	I	E	A	S	W	P	K	I
PDB:1nhx:chainA:P35558	Y	I	I	Q	V	I	E	A	S	W	P	K	I
sp:P35558:PCKGC_HUMAN	Y	I	I	Q	V	I	E	A	S	W	P	K	I
sp:P07379:PCKGC_RAT	Y	I	I	Q	V	I	E	A	S	W	P	K	I
sp:Q9Z2V4:PCKGC_MOUSE	Y	I	I	Q	V	I	E	A	S	W	P	K	I
tr:A8DYI3:A8DYI3_DROME	Y	M	I	F	V	M	K	A	D	W	P	K	I
tr:Q7JXB5:Q7JXB5_DROME	Y	M	I	F	V	M	K	A	D	W	P	K	I
tr:Q7JKI3:Q7JKI3_CAEEL	Y	V	F	Q	Y	L	D	T	S	W	P	K	I
tr:Q7JKI2:Q7JKI2_CAEEL	Y	V	F	Q	Y	L	D	T	S	W	P	K	I
tr:002286:002286_CAEEL	Y	V	F	Q	Y	L	D	T	S	W	P	K	I

	whole protein		domain-based		contact-based	
protein	ident	simil	ident	simil	ident	simil
PDB:1khb:chainA:P35558	0.99	1.0	0.99	1.0	1.0	1.0
PDB:1khf:chainA:P35558	0.99	1.0	0.99	1.0	1.0	1.0
PDB:1khg:chainA:P35558	0.99	1.0	0.99	1.0	1.0	1.0
PDB:1m51:chainA:P35558	0.99	1.0	0.99	1.0	1.0	1.0
PDB:1nhx:chainA:P35558	0.99	1.0	0.99	1.0	1.0	1.0
sp:P35558:PCKGC_HUMAN	1.0	1.0	1.0	1.0	1.0	1.0
sp:P07379:PCKGC_RAT	0.91	0.98	0.91	0.97	1.0	1.0
sp:Q9Z2V4:PCKGC_MOUSE	0.91	0.98	0.91	0.98	1.0	1.0
tr:A8DYI3:A8DYI3_DROME	0.6	0.8	0.61	0.82	0.62	0.8
tr:Q7JXB5:Q7JXB5_DROME	0.62	0.85	0.64	0.88	0.62	0.8
tr:Q7JKI3:Q7JKI3_CAEEL	0.55	0.81	0.56	0.83	0.54	0.83
tr:Q7JKI2:Q7JKI2_CAEEL	0.56	0.84	0.57	0.86	0.54	0.83
tr:002286:002286_CAEEL	0.54	0.82	0.57	0.86	0.54	0.83

TGF-beta receptor type-1

Best gene implication in ageing for this target family came from gene P80204 annotated in UniProt release 2014_02. Annotation GO 7568 (aging) was Inferred from Expression Pattern

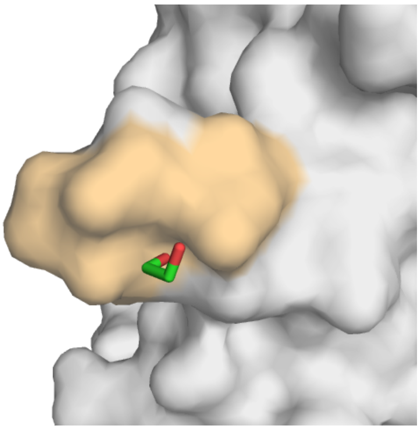

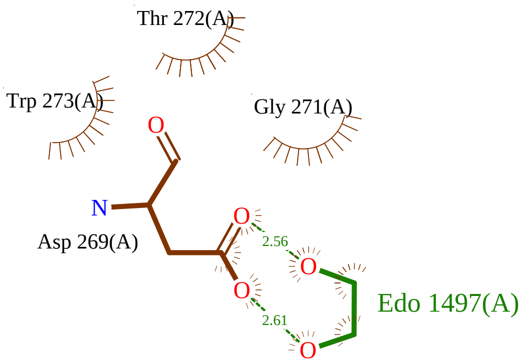

protein	amino acids contacts (binding site)
PDB:2wot:chainA:P36897	S D G T W Q
PDB:2wou:chainA:P36897	S D G T W Q
tr:F8W1R9:F8W1R9_HUMAN	S D G T W Q
tr:B4DY26:B4DY26_HUMAN	S D G T W Q
tr:Q5T7S2:Q5T7S2_HUMAN	S D G T W Q
sp:P36897:TGFR1_HUMAN	S D G T W Q
tr:Q5M9H3:Q5M9H3_RAT	S D G T W Q
sp:P80204:TGFR1_RAT	S D G T W Q
tr:Q3U485:Q3U485_MOUSE	S D G T W Q
tr:Q9D5H8:Q9D5H8_MOUSE	S D G T W Q
tr:E9Q418:E9Q418_MOUSE	S D G T W Q
sp:Q64729:TGFR1_MOUSE	S D G T W Q
tr:Q4FJL1:Q4FJL1_MOUSE	S D G T W Q
tr:Q7YU60:Q7YU60_DROME	S D G T W Q
tr:A1Z7L9:A1Z7L9_DROME	S D G T W Q
tr:Q23975:Q23975_DROME	S D G T W Q
tr:A1Z7L8:A1Z7L8_DROME	S D G T W Q

	whole protein		domain-based		contact-based	
protein	ident	simil	ident	simil	ident	simil
PDB:2wot:chainA:P36897	0.99	1.0	1.0	1.0	1.0	1.0
PDB:2wou:chainA:P36897	0.99	1.0	1.0	1.0	1.0	1.0
tr:F8W1R9:F8W1R9_HUMAN	0.74	0.75	1.0	1.0	1.0	1.0
tr:B4DY26:B4DY26_HUMAN	0.86	0.86	1.0	1.0	1.0	1.0
tr:Q5T7S2:Q5T7S2_HUMAN	1.0	1.0	1.0	1.0	1.0	1.0
sp:P36897:TGFR1_HUMAN	1.0	1.0	1.0	1.0	1.0	1.0
tr:Q5M9H3:Q5M9H3_RAT	0.96	0.98	1.0	1.0	1.0	1.0
sp:P80204:TGFR1_RAT	0.95	0.97	1.0	1.0	1.0	1.0
tr:Q3U485:Q3U485_MOUSE	0.82	0.83	1.0	1.0	1.0	1.0
tr:Q9D5H8:Q9D5H8_MOUSE	0.77	0.8	1.0	1.0	1.0	1.0
tr:E9Q418:E9Q418_MOUSE	0.85	0.86	1.0	1.0	1.0	1.0
sp:Q64729:TGFR1_MOUSE	0.96	0.97	1.0	1.0	1.0	1.0
tr:Q4FJL1:Q4FJL1_MOUSE	0.96	0.97	1.0	1.0	1.0	1.0
tr:Q7YU60:Q7YU60_DROME	0.47	0.69	0.88	0.96	1.0	1.0
tr:A1Z7L9:A1Z7L9_DROME	0.46	0.69	0.88	0.96	1.0	1.0
tr:Q23975:Q23975_DROME	0.46	0.69	0.88	0.96	1.0	1.0
tr:A1Z7L8:A1Z7L8_DROME	0.45	0.67	0.88	0.96	1.0	1.0

babo (FBgn0011300) associated phenotypes

cell growth defective, decreased cell number, developmental rate defective, dominant, flight defective, germline clone, long lived, mitotic cell cycle defective, neuroanatomy defective, phototaxis defective, progressive, size defective, somatic clone

(Information from FlyBase)

DNA mismatch repair protein MLH1

Best gene implication in ageing for this target family came from gene Q5GJ64 via mapping the annotation from Ensembl ENSG00000076242 via mapping the annotation from EntrezGene 4292 via mapping the annotation from GenAgeHuman 0243 annotated in GenAge release 17.

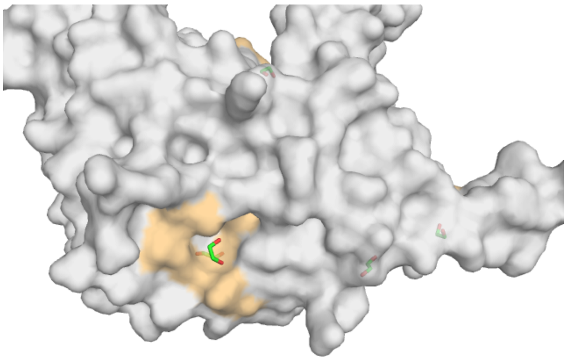

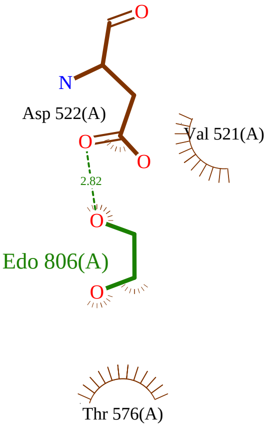

protein	amino acids contacts (binding site)
PDB:4e4w:chainA:P38920	L R K V D I Y Q T V R D M L K E I A N
PDB:4fmn:chainA:P38920	L R K V D I Y Q T V R D M L K E I A N
tr:Q5GJ64:Q5GJ64_HUMAN	L Q E I N M Y Q Y E K S I T E Q L A N
sp:P40692:MLH1_HUMAN	L Q E I N M Y Q Y E K S I T E Q L A N
tr:F1LSD8:F1LSD8_RAT	L Q E I N M Y Q Y E K S I T E Q L A N
sp:Q9JK91:MLH1_MOUSE	L Q E I S M Y Q Y E K S I T E Q L A N
tr:Q8T0N1:Q8T0N1_DROME	M R R V E T Y Q Y A R - - K D E L T N
tr:A1Z7C1:A1Z7C1_DROME	M R R V E T Y Q Y A R - - K D E L T N
tr:O61917:O61917_DROME	M R R V E T Y Q Y A R - - K D E L T N
tr:Q9XU10:Q9XU10_CAEEL	L R E I I M Y Q F E R T L K Q Q L A D
sp:P38920:MLH1_YEAST	L R K V D I Y Q T V R D M L K E I A N

	whole protein		domain-based		contact-based	
protein	ident	simil	ident	simil	ident	simil
PDB:4e4w:chainA:P38920	1.0	1.0	1.0	1.0	1.0	1.0
PDB:4fmn:chainA:P38920	1.0	1.0	1.0	1.0	1.0	1.0
tr:Q5GJ64:Q5GJ64_HUMAN	0.36	0.73	0.31	0.71	0.26	0.7
sp:P40692:MLH1_HUMAN	0.35	0.73	0.31	0.71	0.26	0.7
tr:F1LSD8:F1LSD8_RAT	0.35	0.73	0.31	0.71	0.26	0.7
sp:Q9JK91:MLH1_MOUSE	0.36	0.73	0.31	0.72	0.26	0.68
tr:Q8T0N1:Q8T0N1_DROME	0.2	0.49	0.27	0.65	0.37	0.49
tr:A1Z7C1:A1Z7C1_DROME	0.32	0.66	0.27	0.65	0.37	0.49
tr:O61917:O61917_DROME	0.32	0.66	0.27	0.65	0.37	0.49
tr:Q9XU10:Q9XU10_CAEEL	0.28	0.7	0.24	0.66	0.32	0.61
sp:P38920:MLH1_YEAST	1.0	1.0	1.0	1.0	1.0	1.0

Neurogenic locus notch homolog protein 1

Best gene implication in ageing for this target family came from gene P07207 annotated in UniProt release 2014_02. Annotation GO 8340 (determination of adult lifespan) was Inferred from Mutant Phenotype

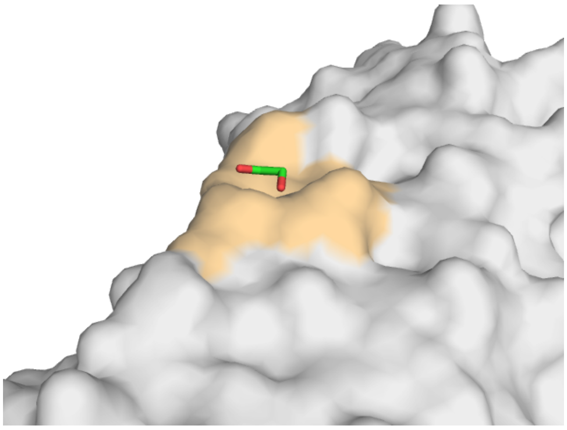

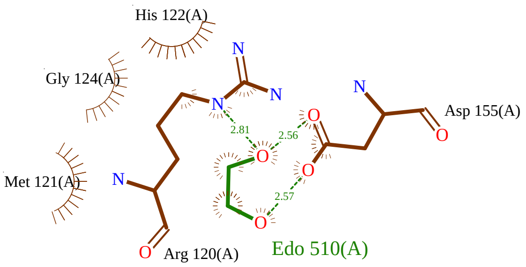

protein	amino acids contacts (binding site)
PDB:2he0:chainA:P46531	- R M H G D
PDB:4cuf:chainA:P46531	C - - - - -
PDB:4d0f:chainA:P46531	C - - - - -
sp:P46531:NOTC1_HUMAN	C R M H G D
sp:Q07008:NOTC1_RAT	C R M H G D
sp:Q01705:NOTC1_MOUSE	C R M H G D
sp:P07207:NOTCH_DROME	C R M H G N

	whole protein		domain-based		contact-based	
protein	ident	simil	ident	simil	ident	simil
PDB:2he0:chainA:P46531	0.98	0.99	0.99	1.0	0.83	0.67
PDB:4cuf:chainA:P46531	0.95	0.99	0.98	1.0	0.17	0.0
PDB:4d0f:chainA:P46531	0.93	0.98	0.98	1.0	0.17	0.0
sp:P46531:NOTC1_HUMAN	1.0	1.0	1.0	1.0	1.0	1.0
sp:Q07008:NOTC1_RAT	0.91	0.97	0.95	0.99	1.0	1.0
sp:Q01705:NOTC1_MOUSE	0.91	0.97	0.95	0.99	1.0	1.0
sp:P07207:NOTCH_DROME	0.33	0.66	0.66	0.9	0.83	0.96

N (FBgn0004647) associated phenotypes

cell adhesion defective, cell non-autonomous, cell shape defective, chemosensitive behavior defective, cold sensitive, courtship behavior defective, decreased cell death, decreased cell number, dominant, flightless, germline clone, gravitaxis defective, heat sensitive, homeotic, hyperplasia, immune response defective, increased cell death, increased cell number, lethal - all die before end of P-stage, lethal - all die before end of pupal stage, lethal - all die during pharate adult stage, maternal effect, memory defective, mitotic cell cycle defective, neuroanatomy defective, neurogenic phenotype, partially, partially lethal - majority die, planar polarity defective, poor, reduced, short lived, size defective, somatic clone, some die during pharate adult stage, some die during pupal stage, sterile, temperature conditional

(Information from FlyBase)

N (UniProt:P07207) annotation

Function: Signaling protein, which regulates, with both positive and negative signals, the differentiation of at least central and peripheral nervous system and eye, wing disk, oogenesis, segmental appendages such as antennae and legs, and muscles, through lateral inhibition or induction. Functions as a receptor for membrane- bound ligands Delta and Serrate to regulate cell-fate determination. Upon ligand activation, and releasing from the cell membrane, the Notch intracellular domain (NICD) forms a transcriptional activator complex with Su(H) (Suppressor of hairless) and activates genes of the E(spl) complex. Essential for proper differentiation of ectoderm. Fringe (fng) acts in the Golgi to determine the type of O-linked fucose on the EGF modules in N, altering the ability of N to bind with Delta (Dl). O-fut1 also has a role in modulating the interaction. Rumi acts in the endoplasmic reticulum to glucosylate the EGF modules in N, this is required for correct folding and cleavage of N. (PubMed:10935637, PubMed:18243100).

Subunit: Homomer. Interacts with Su(H) when activated. Interacts with Dx via its ANK repeats. Interacts with Dl via the EGF repeats and the Dl EGF repeats. Interacts with Nedd4 and Su(dx). Interacts with O-fut1; the interaction glycosylates N and transports N to early endosomes. (PubMed:10935637, PubMed:14573873, PubMed:15620649, PubMed:15620650, PubMed:17329366, PubMed:7671825, PubMed:8162848).
Subcellular location: Cell membrane ECO:0000269—PubMed:17329366; Single-pass type I membrane protein (PubMed:17329366). Endosome (PubMed:17329366). Note=Transported to early endosomes by O-fut1.
Subcellular location: Processed neurogenic locus Notch protein: Nucleus. Note=Upon activation and S3 cleavage, it is released from the cell membrane and enters into the nucleus in conjunction with Su(H).
Domain: Crystal structure of the ANK repeat domain shows that there are 7 repeats and the stabilizing C-terminal repeat enhances the protein stability by extending the ankyrin domain. (PubMed:14573873).
Ptm: Upon binding its ligands such as Delta or Serrate, it is cleaved (S2 cleavage) in its extracellular domain, close to the transmembrane domain. S2 cleavage is probably mediated by Kuz. It is then cleaved (S3 cleavage) downstream of its transmembrane domain, releasing it from the cell membrane. S3 cleavage requires Psn.
Ptm: O-glycosylated. O-fucosylated by O-fut1 in the EGF repeat doamin which inhibits both Serrate/Ser- and Delta/Dl-binding. (PubMed:12909620).
Ptm: Ubiquitinated by Nedd4; which promotes ligand-independent endocytosis and proteasomal degradation. May also be ubiquitinated by Su(dx). (PubMed:15620649).

(Information from UniProt)

Mitogen-activated protein kinase 14

Best gene implication in ageing for this target family came from gene O62618 annotated in UniProt release 2014_02. Annotation GO 8340 (determination of adult lifespan) was Inferred from Mutant Phenotype

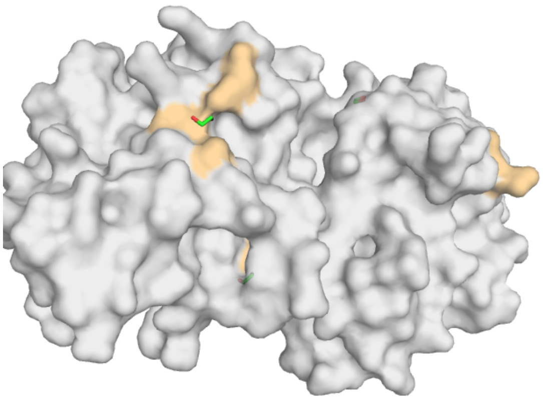

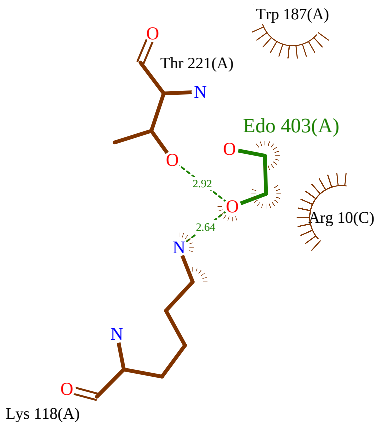

protein	amino acids contacts (binding site)												
PDB:4lop:chainA:P47811	Y	R	Q	T	G	H	K	I	I	R	W	Y	T
PDB:4loq:chainA:P47811	Y	R	Q	T	G	H	K	I	I	R	W	Y	T
sp:Q16539:MK14_HUMAN	Y	R	Q	T	G	L	K	I	I	R	W	Y	T
tr:L7RSM2:L7RSM2_HUMAN	Y	R	Q	T	G	L	K	I	I	R	W	Y	T
tr:G3V617:G3V617_RAT	Y	R	Q	T	G	H	K	I	I	R	W	Y	T
tr:Q56A33:Q56A33_RAT	Y	R	Q	T	G	H	K	I	I	R	W	Y	T
tr:B2KF35:B2KF35_MOUSE	Y	R	Q	T	G	H	K	I	I	R	W	Y	T
sp:P47811:MK14_MOUSE	Y	R	Q	T	G	H	K	I	I	R	W	Y	T
tr:Q5U421:Q5U421_MOUSE	Y	R	Q	T	G	H	K	I	I	R	W	Y	T
sp:O62618:MK14A_DROME	Y	K	Q	T	N	M	R	V	I	R	W	Y	T
tr:E1JIV6:E1JIV6_DROME	Y	K	Q	T	N	M	R	V	I	R	W	Y	T
sp:O61443:MK14B_DROME	Y	K	Q	T	S	T	R	V	I	R	W	Y	T
sp:Q17446:PMK1_CAEEL	Y	V	I	S	G	T	K	I	I	R	W	Y	T
sp:Q8MXI4:PMK2_CAEEL	H	E	N	T	G	D	K	I	I	R	W	Y	P
sp:P32485:HOG1_YEAST	I	R	N	T	S	Q	Q	V	I	R	Y	Y	P

protein	whole protein ident	whole protein simil	domain-based ident	domain-based simil	contact-based ident	contact-based simil
PDB:4lop:chainA:P47811	1.0	1.0	1.0	1.0	1.0	1.0
PDB:4loq:chainA:P47811	1.0	1.0	1.0	1.0	1.0	1.0
sp:Q16539:MK14_HUMAN	0.99	1.0	0.99	1.0	0.92	0.92
tr:L7RSM2:L7RSM2_HUMAN	0.96	0.99	0.95	0.99	0.92	0.92
tr:G3V617:G3V617_RAT	1.0	1.0	1.0	1.0	1.0	1.0
tr:Q56A33:Q56A33_RAT	0.96	0.99	0.95	0.99	1.0	1.0
tr:B2KF35:B2KF35_MOUSE	0.71	0.71	0.81	0.82	1.0	1.0
sp:P47811:MK14_MOUSE	1.0	1.0	1.0	1.0	1.0	1.0
tr:Q5U421:Q5U421_MOUSE	0.96	0.99	0.96	0.99	1.0	1.0
sp:O62618:MK14A_DROME	0.67	0.88	0.72	0.9	0.62	0.81
tr:E1JIV6:E1JIV6_DROME	0.67	0.88	0.72	0.9	0.62	0.81
sp:O61443:MK14B_DROME	0.68	0.89	0.74	0.92	0.62	0.86
sp:Q17446:PMK1_CAEEL	0.61	0.84	0.67	0.88	0.69	0.76
sp:Q8MXI4:PMK2_CAEEL	0.49	0.74	0.54	0.78	0.62	0.77
sp:P32485:HOG1_YEAST	0.41	0.68	0.52	0.84	0.38	0.8

Mpk2 (FBgn0015765) associated phenotypes

heat stress response defective, immune response defective, non-enhancer of variegation, non-suppressor of variegation, nutrition conditional, osmotic stress response defective, oxidative stress response defective, partially lethal - majority die, reduced, short lived, starvation stress response defective, stress response defective

(Information from FlyBase)

Mpk2 (UniProt:O62618) annotation

Function: Kinase involved in a signal transduction pathway. May down-regulate insect immunity gene expression after prolonged infection. (PubMed:9417090, PubMed:9584193).
Cofactor:Mg(2+)
Enzyme regulation: Activated by threonine and tyrosine phosphorylation by Mkk3 in response to environmental stress. (PubMed:9417090, PubMed:9584193).
Subcellular location: Nucleus (PubMed:9417090).
Developmental stage: Expressed both maternally and zygotically. Levels are highest at the preblastoderm stage but low levels are present throughout development. (PubMed:9584193).
Domain: The TXY motif contains the threonine and tyrosine residues whose phosphorylation activates the MAP kinases.
Ptm: Dually phosphorylated on Thr-184 and Tyr-186, which activates the enzyme. (PubMed:9417090).

(Information from UniProt)

p38b (FBgn0024846) associated phenotypes

RU486 conditional, circadian rhythm defective, decreased cell size, developmental rate defective,

drug conditional, heat stress response defective, immune response defective, locomotor behavior defective, long lived, melanotic mass phenotype, neuroanatomy defective, nutrition conditional, osmotic stress response defective, oxidative stress response defective, partially lethal - majority die, progressive, short lived, small body, starvation stress response defective

(Information from FlyBase)

p38b (UniProt:O61443) annotation
Function: Kinase involved in dpp signal transduction pathway in the process of wing morphogenesis when the levels of dpp are enhanced or inhibited. May down-regulate insect immunity gene expression after prolonged infection. (PubMed:10022918, PubMed:9584193).
Cofactor:Mg(2+)
Enzyme regulation: Activated by threonine and tyrosine phosphorylation by Mkk3. (PubMed:9584193).
Subcellular location: Nucleus.
Tissue specificity: At mid-embryogenesis, highest expression is seen in developing anterior and posterior midguts. Almost ubiquitous expression throughout all development.
Developmental stage: Expressed both maternally and zygotically in the embryo, expression seen in all developmental stages.
Domain: The TXY motif contains the threonine and tyrosine residues whose phosphorylation activates the MAP kinases.
Ptm: Dually phosphorylated on Thr-183 and Tyr-185, which activates the enzyme.

(Information from UniProt)

pmk-1 (WBGene00004055) associated phenotypes
cell stress response variant, drug induced gene expression variant, gene expression level reduced, lethal, life span variant, organism oxidative stress response hypersensitive, pathogen induced gene expression variant, pathogen susceptibility increased, sterile

(Information from WormBase)

pmk-1 (UniProt:Q17446) annotation
Function: Responds to activation by environmental stress and pro- inflammatory cytokines by phosphorylating downstream targets. Functions downstream of the MAPKK sek-1 and the MAPKKK nsy-1 as the MAP kinase required for pathogen resistance. (PubMed:11703092, PubMed:12142542).
Cofactor: Mg(2+) Evidence=(PubMed:11703092);
Enzyme regulation: Activated by phosphorylation on threonine and tyrosine. Inhibited by pyridinyl-imidazole related compounds. (PubMed:11703092).
Domain: The TXY motif contains the threonine and tyrosine residues whose phosphorylation activates the MAP kinases.
Ptm: Dually phosphorylated on Thr-191 and Tyr-193, which activates the enzyme.

(Information from UniProt)

pmk-2 (UniProt:Q8MXI4) annotation
Function: Responds to activation by environmental stress and pro- inflammatory cytokines by phosphorylating downstream targets. (PubMed:11703092).
Cofactor: Mg(2+) Evidence=(PubMed:11703092);
Enzyme regulation: Activated by phosphorylation on threonine and tyrosine. Inhibited by pyridinyl-imidazole related compounds. (PubMed:11703092).
Subcellular location: Cytoplasm (PubMed:11703092).
Domain: The TXY motif contains the threonine and tyrosine residues whose phosphorylation activates the MAP kinases.
Ptm: Dually phosphorylated on Thr-222 and Tyr-224, which activates the enzyme.

(Information from UniProt)

Mitogen-activated protein kinase 10

Best gene implication in ageing for this target family came from gene Q8WQG9 annotated in UniProt release 2014_02. Annotation GO 8340 (determination of adult lifespan) was Inferred from Mutant Phenotype

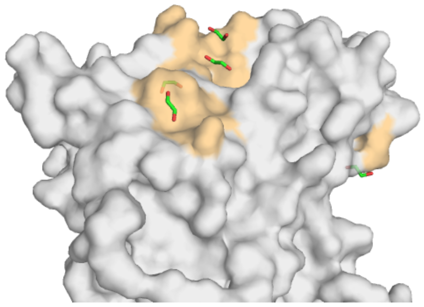

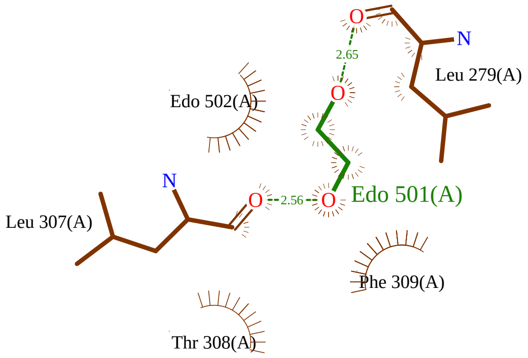

protein	amino acids contacts (binding site)
PDB:2r9s:chainA:P53779	E N K A A Y N Q G Y R Q L C P E Q A G L T F P K L P D S R D K I D H I
PDB:3fi2:chainA:P53779	E N K A A Y N Q G Y R Q L C P E Q A G L T F P K L P D S R D K I D H I
PDB:3fi3:chainA:P53779	E N K A A Y N Q G Y R Q L C P E Q A G L T F P K L P D S R D K I D H I
PDB:3fv8:chainA:P53779	E N K A A Y N Q G Y R Q L C P E Q A G L T F P K L P D S R D K I D H I
sp:P53779:MK10_HUMAN	E N K A A Y N Q G Y R Q L C P E Q A G L T F P K L P D S R D K I D H I
tr:B0VXR6:B0VXR6_RAT	E N K A A Y N Q G Y R Q L C P E Q A G L T F P K L P D S R D K I D H I
tr:D3ZQ33:D3ZQ33_RAT	E N K A A Y N Q G Y R Q L C P E Q A G L T F P K L P D S R D K I D H I
tr:Q3TQZ7:Q3TQZ7_MOUSE	E N K A A Y N Q G Y R Q L C P E Q A G L T F P K L P D S R D K I D H I
tr:Q80W82:Q80W82_MOUSE	E N K A A Y N Q G Y R Q L C P E Q A G L T F P K L P D S R D K I D H I
tr:Q78GB8:Q78GB8_MOUSE	E N K A A Y N Q G Y R Q L C P E Q A G L T F P K L P D S R D K I D H I
tr:Q8C9D4:Q8C9D4_MOUSE	E N K A A Y N Q G Y R Q L C P E Q A G L T F P K L P D S R D K I D H I
tr:E9QN59:E9QN59_MOUSE	E N K A A Y N Q G Y R Q L C P E Q A G L T F P K L P D S R D K I D H I
tr:E1JHD6:E1JHD6_DROME	E N R A A Y N V G Y R Q L S P S Q T G Y S F D R L P D S R N K I E H V
sp:P92208:JNK_DROME	E N R A A Y N V G Y R Q L S P S Q T G Y S F D R L P D S R N K I E H V
sp:Q8WQG9:JNK1_CAEEL	T N R S A F Q V G Y R Q L D R S Q Q A T P F E V L S D A R D R I D H V
sp:P32485:HOG1_YEAST	Q D N S A T P A Q K E L L P K D S D P I P F S E R - - P V D K I D H V

	whole protein		domain-based		contact-based	
protein	ident	simil	ident	simil	ident	simil
PDB:2r9s:chainA:P53779	0.99	0.99	0.99	0.99	1.0	1.0
PDB:3fi2:chainA:P53779	0.98	0.99	0.98	0.99	1.0	1.0
PDB:3fi3:chainA:P53779	0.99	0.99	0.99	0.99	1.0	1.0
PDB:3fv8:chainA:P53779	0.98	0.99	0.98	0.99	1.0	1.0
sp:P53779:MK10_HUMAN	1.0	1.0	1.0	1.0	1.0	1.0
tr:B0VXR6:B0VXR6_RAT	0.92	0.92	1.0	1.0	1.0	1.0
tr:D3ZQ33:D3ZQ33_RAT	0.99	1.0	1.0	1.0	1.0	1.0
tr:Q3TQZ7:Q3TQZ7_MOUSE	0.89	0.9	1.0	1.0	1.0	1.0
tr:Q80W82:Q80W82_MOUSE	0.89	0.9	1.0	1.0	1.0	1.0
tr:Q78GB8:Q78GB8_MOUSE	0.92	0.92	1.0	1.0	1.0	1.0
tr:Q8C9D4:Q8C9D4_MOUSE	1.0	1.0	1.0	1.0	1.0	1.0
tr:E9QN59:E9QN59_MOUSE	0.94	0.94	1.0	1.0	1.0	1.0
tr:E1JHD6:E1JHD6_DROME	0.62	0.74	0.78	0.93	0.66	0.81
sp:P92208:JNK_DROME	0.62	0.74	0.78	0.93	0.66	0.81
sp:Q8WQG9:JNK1_CAEEL	0.45	0.6	0.63	0.82	0.46	0.63
sp:P32485:HOG1_YEAST	0.29	0.64	0.38	0.76	0.23	0.47

bsk (FBgn0000229) associated phenotypes

RU486 conditional, cell death defective, chemical resistant, chemical sensitive, conditional, decreased

cell growth, dominant, heat sensitive, increased cell death, long lived, neuroanatomy defective, neurophysiology defective, nutrition conditional, oxidative stress response defective, planar polarity defective, progressive, short lived, somatic clone, starvation stress response defective, stress response defective, wound healing defective

(Information from FlyBase)

bsk (UniProt:P92208) annotation

Function: Responds to activation by environmental stress by phosphorylating a number of transcription factors, primarily components of AP-1 such as Jra and also the transcriptional repressor aop, and thus regulates transcriptional activity. Component of the immune response activated by bacterial infection, and is involved in wound healing and in dorsal closure, a morphogenetic movement during embryogenesis. Controls the expression of a phosphatase, puckered, at the edges of wounded epidermal tissue and in the dorsal epithelium during dorsal closure. (PubMed:10433922, PubMed:11784101, PubMed:8946915, PubMed:9224720).

Cofactor:Mg(2+)

Enzyme regulation: Activated by threonine and tyrosine phosphorylation by the dual specificity kinase, hep. Inhibited by dual specificity phosphatase, puckered. (PubMed:10433922).

Subcellular location: Cytoplasm

Tissue specificity: During gastrulation, expression is seen in cells undergoing morphogenetic movements. By stage 9 of embryonic development, expression is ubiquitous. At stages 12-14, expression occurs in epidermis and central nervous system. At stage 15, expression is restricted to ventral nerve cord, brain and some peripheral neurons. In larvae, expression is seen in all imaginal disks, with highest levels in wing and eye disks, and in the CNS. Adults express the protein in fat body and hemocytes. (PubMed:8946915, PubMed:8946916).

Developmental stage: Expressed maternally and zygotically through to adult (male and female). (PubMed:8946915).

Domain: The TXY motif contains the threonine and tyrosine residues whose phosphorylation activates the MAP kinases.

Ptm: Dually phosphorylated on Thr-181 and Tyr-183, which activates the enzyme.

(Information from UniProt)

jnk-1 (WBGene00002178) associated phenotypes

aldicarb hypersensitive, pathogen resistance increased, protein phosphorylation reduced, shortened life span, thermotolerance reduced

(Information from WormBase)

jnk-1 (UniProt:Q8WQG9) annotation

Function: Responds to activation by environmental stress by phosphorylating a number of transcription factors, and thus regulates transcriptional activity (By similarity). May coordinate locomotion by acting via type-D GABAergic motor neurons and regulate synaptic vesicle transport in conjunction with unc-16. (, PubMed:10393177, PubMed:11738026).

Cofactor:Mg(2+) Evidence=(PubMed:10393177);

Enzyme regulation: Activated by threonine and tyrosine phosphorylation by either of the dual specificity kinases, jkk-1 and sek-1. (PubMed:10393177).

Subunit: Binds to the scaffolding protein, unc-16. Unc-16 also binds other components of the JNK signaling pathway. (PubMed:11738026).

Domain: The TXY motif contains the threonine and tyrosine residues whose phosphorylation activates the MAP kinases.

Ptm: Dually phosphorylated on Thr-276 and Tyr-278, which activates the enzyme.

(Information from UniProt)

Bloom syndrome protein

Best gene implication in ageing for this target family came from gene O18017 annotated in UniProt release 2014_02. Annotation GO 8340 (determination of adult lifespan) was Inferred from Mutant Phenotype

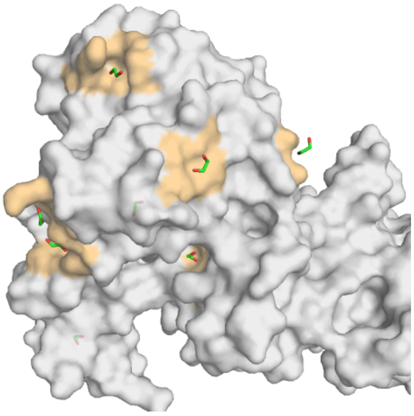

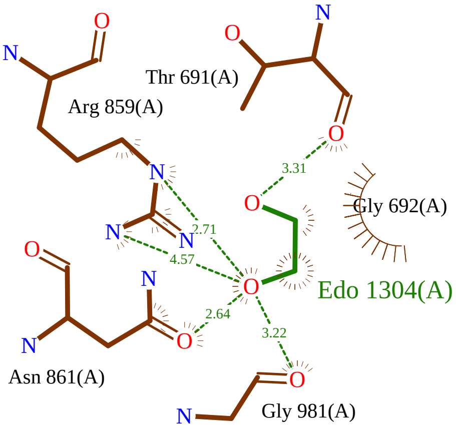

protein	amino acids contacts (binding site)
PDB:4o3m:chainA:P54132	H K M K H G H T G Y K L L S V R D P Q V R N N G G K V H L V K W
sp:P54132:BLM_HUMAN	H K M K H G H T G Y K L L S V R D P Q V R N N G G K V H L V K W
tr:D3ZQW1:D3ZQW1_RAT	H K M K R G H T G Y K L L S V R D P Q V R N N G G K V H L V K W
tr:E9PZ97:E9PZ97_MOUSE	H K M K H G H T G Y K L L S V R D P Q V R N N G G K V H L V K W
sp:O88700:BLM_MOUSE	H K M K H G H T G Y K L L S V R D P Q V R N N G G K V H L V K W
sp:Q9VGI8:BLM_DROME	G T M H S G K T G N N Y I N V R D C K W R N T G A S V T C V N E
sp:O18017:BLM_CAEEL	H K Y D K G N T G H G L L P V K D S K L R N A G A E L S L M T Q
sp:P35187:SGS1_YEAST	R D L Y H K P T G Y G K L D I Q D P V F R N A G G N P P A V T -

	whole protein		domain-based		contact-based	
protein	ident	simil	ident	simil	ident	simil
PDB:4o3m:chainA:P54132	1.0	1.0	1.0	1.0	1.0	1.0
sp:P54132:BLM_HUMAN	1.0	1.0	1.0	1.0	1.0	1.0
tr:D3ZQW1:D3ZQW1_RAT	0.75	0.91	0.89	0.96	0.97	0.99
tr:E9PZ97:E9PZ97_MOUSE	0.76	0.91	0.91	0.97	1.0	1.0
sp:O88700:BLM_MOUSE	0.76	0.91	0.91	0.97	1.0	1.0
sp:Q9VGI8:BLM_DROME	0.27	0.62	0.44	0.77	0.38	0.54
sp:O18017:BLM_CAEEL	0.22	0.48	0.4	0.69	0.41	0.65
sp:P35187:SGS1_YEAST	0.21	0.55	0.29	0.6	0.38	0.54

Blm (FBgn0002906) associated phenotypes

DNA repair defective, chemical sensitive, dominant, increased cell death, maternal effect, mitotic cell cycle defective, partially, radiation sensitive

(Information from FlyBase)

Blm (UniProt:Q9VGI8) annotation

Function: Participates in DNA replication and repair. Exhibits a magnesium-dependent ATP-dependent DNA-helicase activity that unwinds single- and double-stranded DNA in a 3'-5' direction (By similarity).
Subcellular location: Nucleus

(Information from UniProt)

him-6 (WBGene00001865) associated phenotypes

XXX progeny, autosomal nondisjunction meiosis, embryonic lethal, germline mortal, high incidence male progeny, male fertility reduced, male sterile, nullo X gamete, reduced brood size

(Information from WormBase)

him-6 (UniProt:O18017) annotation

Function: Participates in DNA replication and repair. Exhibits a magnesium-dependent ATP-dependent DNA-helicase activity that unwinds single- and double-stranded DNA in a 3'-5' direction (By similarity).

Subcellular location: Nucleus

(Information from UniProt)

Tyrosine-protein phosphatase non-receptor type 11

Best gene implication in ageing for this target family came from gene G5EC24 via mapping the annotation from WormBase WBGene00004214 annotated in WormBase 2014-03-11. Annotation GO 8340 (determination of adult lifespan) was Inferred from Mutant Phenotype

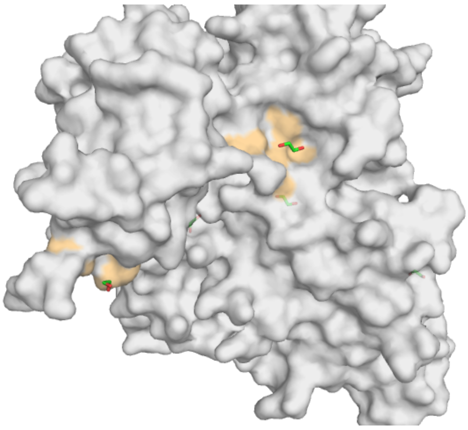

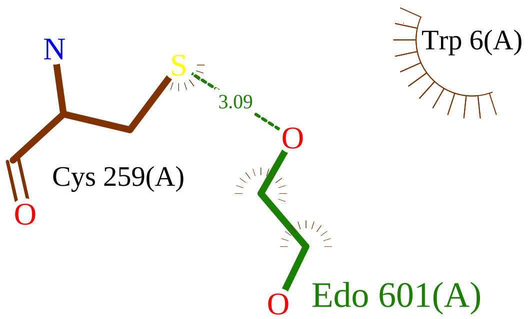

protein	amino acids contacts (binding site)
PDB:4gwf:chainA:Q06124	S R W F F T G Y L A K S F E Q C H G E Q V R K V N T E R W G V R S Q Q M Q H
PDB:4h1o:chainA:Q06124	S R W F F T G Y L A K S F E Q C H - E Q V R K V N T E R W G V R S Q Q M Q H
sp:Q06124:PTN11_HUMAN	S R W F F T G Y L A K S F E Q C H G E Q V R K V N T E R W G V R S Q Q M Q H
sp:P41499:PTN11_RAT	S R W F F T G Y L A K S F E Q C H G E Q V R K V N T E R W G V R S Q Q M Q H
sp:P35235:PTN11_MOUSE	S R W F F T G Y L A K S F E Q C H G E Q V R K V N T E R W G V R S Q Q M Q H
sp:P29349:CSW_DROME	S R W F F N G F L P K - F E Q S H V S V V N R H Q P A R W G V R S Q Q Y Q H
tr:G5EC24:G5EC24_CAEEL	R R Y F F E S L I T K - F D S A H - F E I S R D K D T R W G C R T Y Q K A F

	whole protein		domain-based		contact-based	
protein	ident	simil	ident	simil	ident	simil
PDB:4gwf:chainA:Q06124	1.0	1.0	1.0	1.0	1.0	1.0
PDB:4h1o:chainA:Q06124	1.0	1.0	1.0	1.0	0.97	0.95
sp:Q06124:PTN11_HUMAN	1.0	1.0	1.0	1.0	1.0	1.0
sp:P41499:PTN11_RAT	0.99	1.0	0.99	1.0	1.0	1.0
sp:P35235:PTN11_MOUSE	0.99	1.0	1.0	1.0	1.0	1.0
sp:P29349:CSW_DROME	0.41	0.57	0.49	0.66	0.61	0.72
tr:G5EC24:G5EC24_CAEEL	0.32	0.64	0.35	0.67	0.29	0.46

csw (FBgn0000382) associated phenotypes

dominant, germline clone, heat sensitive, increased cell death, lethal - all die before end of pupal stage, maternal effect, rescuable maternal effect, somatic clone, some die during pupal stage

(Information from FlyBase)

csw (UniProt:P29349) annotation

Function: Required in all receptor tyrosine kinase signaling pathways. Functions downstream of the receptor tyrosine kinase torso, acting in concert with D-Raf via tailless. Also functions downstream of Egfr (epidermal growth factor receptor) and btl (fibroblast growth factor receptor). The SH2 domain suggests that csw effects its role by mediating heteromeric protein interactions. Ma-

ternally required for normal determination of cell fates at the termini of the embryo. Required for cell fate specification of the ventral ectoderm, in the developing embryonic CNS and for embryonic tracheal cell migration. Functions during imaginal development for proper formation of adult structures such as eyes, aristae, L5 wing vein and the tarsal claw. (PubMed:1638629, PubMed:8948575).
Subcellular location: Cytoplasm.
Tissue specificity: Expressed uniformly throughout all tissues during embryogenesis. (PubMed:1638629).
Developmental stage: Expressed throughout development. (PubMed:1638629).
Miscellaneous: The PTPase domain is interrupted by a PTPase insert which shares no homologies with other PTPase proteins. This PTPase insert is reminiscent of the kinase insert within the kinase catalytic domains of several receptor tyrosine kinases.

(Information from UniProt)

ptp-2 (UniProt:G5EC24) annotation
Subcellular location: Cytoplasm ECO:0000256—PIRNR:PIRNR000929.

(Information from UniProt)

DNA repair protein RAD51 homolog 1

Best gene implication in ageing for this target family came from gene P25454 via mapping the annotation from Ensembl YER095W via mapping the annotation from EntrezGene 856831 via mapping the annotation from GenAgeModels 0533 annotated in GenAge release 17.

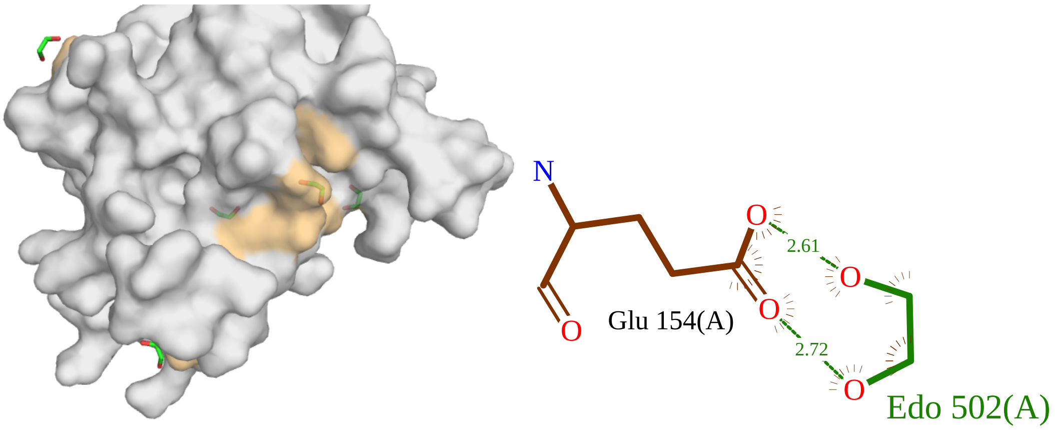

protein	amino acids contacts (binding site)
PDB:1n0w:chainA:Q06609	E F T Q E T E T R R A L L N A R I E M
sp:Q06609:RAD51_HUMAN	E F T Q E T E T R R A L L N A R I E M
tr:B5DF04:B5DF04_RAT	E F T Q E T E T R R A L L N A R I E M
sp:Q08297:RAD51_MOUSE	E F T Q E T E T R R A L L N A R I E M
sp:Q27297:RAD51_DROME	E F T Q E T E T R R A L L N A R I E M
tr:C6SV45:C6SV45_DROME	E F T Q E T E T R R A L L N A R I E M
tr:G5EGG8:G5EGG8_CAEEL	E Y T Q E T N T R R A H L N A R V E T
tr:L8E822:L8E822_CAEEL	E Y T Q E T N T R R A H L N A R V E T
tr:Q95Q25:Q95Q25_CAEEL	E Y T Q E T N T R R A H L N A R V E T
sp:P25454:RAD51_YEAST	E F S Q E T E T R R A L M N A R L E V

protein	whole protein ident	simil	domain-based ident	simil	contact-based ident	simil
PDB:1n0w:chainA:Q06609	1.0	1.0	1.0	1.0	1.0	1.0
sp:Q06609:RAD51_HUMAN	1.0	1.0	1.0	1.0	1.0	1.0
tr:B5DF04:B5DF04_RAT	0.99	1.0	1.0	1.0	1.0	1.0
sp:Q08297:RAD51_MOUSE	0.99	1.0	1.0	1.0	1.0	1.0
sp:Q27297:RAD51_DROME	0.65	0.87	0.75	0.91	1.0	1.0
tr:C6SV45:C6SV45_DROME	0.65	0.87	0.75	0.91	1.0	1.0
tr:G5EGG8:G5EGG8_CAEEL	0.56	0.83	0.64	0.89	0.74	0.86
tr:L8E822:L8E822_CAEEL	0.55	0.82	0.64	0.89	0.74	0.86
tr:Q95Q25:Q95Q25_CAEEL	0.5	0.75	0.64	0.89	0.74	0.86
sp:P25454:RAD51_YEAST	0.57	0.77	0.7	0.9	0.79	0.95

spn-A (FBgn0003479) associated phenotypes

DNA repair defective, dominant, lethal - all die before end of pupal stage, maternal effect, meiotic cell cycle defective, radiation sensitive, some die during pupal stage

(Information from FlyBase)

spn-A (UniProt:Q27297) annotation

Function: Binds to single and double-stranded DNA and exhibits DNA-dependent ATPase activity. Underwinds duplex DNA (By similarity).
Function: Spindle genes are required for each of the symmetry- breaking steps that generate polarity during egg axis formation; oocyte positioning at the posterior of the cyst to generate the first AP polarity and inhibition of gurken (grk) signaling to the follicle cell layer to polarize first the AP axis and then DV axis. May have a role in female meiosis. (PubMed:8625736, PubMed:9362456).
Subcellular location: Nucleus
Tissue specificity: Highly expressed in ovaries. (PubMed:8625736).

(Information from UniProt)

Histone acetyltransferase ESA1

Best gene implication in ageing for this target family came from gene Q92794 annotated in UniProt release 2014_02. Annotation GO subterm of 7568 (aging) was Inferred from Mutant Phenotype

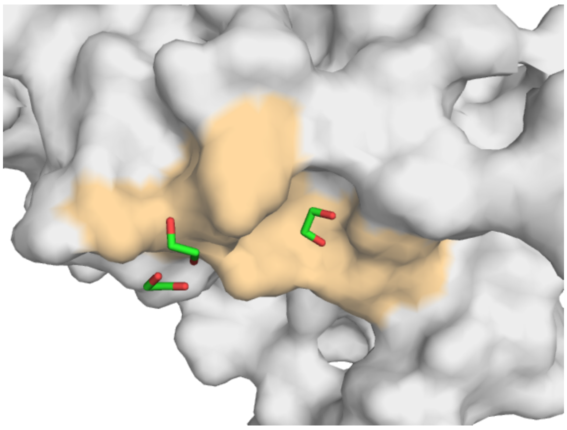

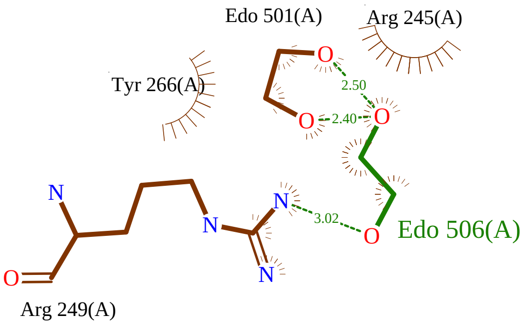

protein	amino acids contacts (binding site)						
PDB:3to9:chainA:Q08649	Q	Y	R	T	R	N	Y
tr:C9K0F9:C9K0F9_HUMAN	K	Y	T	I	Q	N	Y
tr:A5PLL3:A5PLL3_HUMAN	K	Y	T	I	Q	N	Y
tr:A5PKX7:A5PKX7_HUMAN	K	Y	T	I	Q	N	Y
sp:Q92794:KAT6A_HUMAN	K	Y	T	I	Q	N	Y
tr:Q3V1G6:Q3V1G6_MOUSE	K	Y	T	I	Q	N	Y
tr:Q3UPM9:Q3UPM9_MOUSE	K	Y	T	I	Q	N	Y
tr:G3X940:G3X940_MOUSE	K	Y	T	I	Q	N	Y
tr:D0IQI5:D0IQI5_DROME	K	Y	K	I	Q	N	Y
tr:Q9W1A9:Q9W1A9_DROME	K	Y	K	I	Q	N	Y
tr:L8E6K3:L8E6K3_CAEEL	F	Y	K	E	Q	T	Y
tr:Q21789:Q21789_CAEEL	F	Y	K	E	Q	T	Y
tr:D7SFM3:D7SFM3_CAEEL	F	Y	K	E	Q	T	Y
tr:K8ESF3:K8ESF3_CAEEL	F	Y	K	E	Q	T	Y
tr:G5EGU3:G5EGU3_CAEEL	F	Y	K	E	Q	T	Y
tr:K8ES81:K8ES81_CAEEL	F	Y	K	E	Q	T	Y
tr:G5EGP0:G5EGP0_CAEEL	F	Y	K	E	Q	T	Y
tr:K8F7U3:K8F7U3_CAEEL	F	Y	K	E	Q	T	Y
tr:G5EBH5:G5EBH5_CAEEL	F	Y	K	E	Q	T	Y
tr:K8ERR8:K8ERR8_CAEEL	F	Y	K	E	Q	T	Y
sp:Q08649:ESA1_YEAST	Q	Y	R	T	R	N	Y

	whole protein		domain-based		contact-based	
protein	ident	simil	ident	simil	ident	simil
PDB:3to9:chainA:Q08649	0.99	1.0	0.99	1.0	1.0	1.0
tr:C9K0F9:C9K0F9_HUMAN	0.16	0.3	0.58	0.85	0.43	0.67
tr:A5PLL3:A5PLL3_HUMAN	0.18	0.38	0.58	0.85	0.43	0.67
tr:A5PKX7:A5PKX7_HUMAN	0.13	0.27	0.58	0.85	0.43	0.67
sp:Q92794:KAT6A_HUMAN	0.07	0.16	0.58	0.85	0.43	0.67
tr:Q3V1G6:Q3V1G6_MOUSE	0.13	0.27	0.58	0.85	0.43	0.67
tr:Q3UPM9:Q3UPM9_MOUSE	0.11	0.24	0.58	0.85	0.43	0.67
tr:G3X940:G3X940_MOUSE	0.07	0.16	0.58	0.85	0.43	0.67
tr:D0IQI5:D0IQI5_DROME	0.13	0.3	0.53	0.85	0.43	0.71
tr:Q9W1A9:Q9W1A9_DROME	0.06	0.14	0.53	0.85	0.43	0.71
tr:L8E6K3:L8E6K3_CAEEL	0.22	0.51	0.46	0.82	0.29	0.62
tr:Q21789:Q21789_CAEEL	0.21	0.49	0.46	0.82	0.29	0.62
tr:D7SFM3:D7SFM3_CAEEL	0.1	0.23	0.46	0.82	0.29	0.62
tr:K8ESF3:K8ESF3_CAEEL	0.1	0.23	0.46	0.82	0.29	0.62
tr:G5EGU3:G5EGU3_CAEEL	0.1	0.23	0.46	0.82	0.29	0.62
tr:K8ES81:K8ES81_CAEEL	0.1	0.23	0.46	0.82	0.29	0.62
tr:G5EGP0:G5EGP0_CAEEL	0.09	0.2	0.46	0.82	0.29	0.62
tr:K8F7U3:K8F7U3_CAEEL	0.09	0.2	0.46	0.82	0.29	0.62
tr:G5EBH5:G5EBH5_CAEEL	0.08	0.2	0.46	0.82	0.29	0.62
tr:K8ERR8:K8ERR8_CAEEL	0.08	0.2	0.46	0.82	0.29	0.62
sp:Q08649:ESA1_YEAST	1.0	1.0	1.0	1.0	1.0	1.0

enok-RA (UniProt:D0IQI5) annotation
Subcellular location: Nucleus ECO:0000256—RuleBase:RU361211.

(Information from UniProt)

enok (FBgn0034975) associated phenotypes
female semi-sterile, germline clone, neuroanatomy defective, size defective, somatic clone

(Information from FlyBase)

enok (UniProt:Q9W1A9) annotation
Subcellular location: Nucleus ECO:0000256—RuleBase:RU361211.

(Information from UniProt)

lsy-12 (WBGene00045419) associated phenotypes
egg laying defective, locomotion variant, loss of asymmetry ASE, protruding vulva, reduced brood size, sick

(Information from WormBase)

lsy-12 (UniProt:L8E6K3) annotation
Subcellular location: Nucleus ECO:0000256—RuleBase:RU361211.

(Information from UniProt)

lsy-12 (UniProt:Q21789) annotation
Subcellular location: Nucleus ECO:0000256—RuleBase:RU361211.

(Information from UniProt)

lsy-12 (UniProt:D7SFM3) annotation
Subcellular location: Nucleus ECO:0000256—RuleBase:RU361211.

(Information from UniProt)

lsy-12 (UniProt:K8ESF3) annotation
Subcellular location: Nucleus ECO:0000256—RuleBase:RU361211.

(Information from UniProt)

lsy-12 (UniProt:G5EGU3) annotation
Subcellular location: Nucleus ECO:0000256—RuleBase:RU361211.

(Information from UniProt)

lsy-12 (UniProt:K8ES81) annotation
Subcellular location: Nucleus ECO:0000256—RuleBase:RU361211.

(Information from UniProt)

lsy-12 (UniProt:G5EGP0) annotation
Subcellular location: Nucleus ECO:0000256—RuleBase:RU361211.

(Information from UniProt)

lsy-12 (UniProt:K8F7U3) annotation
Subcellular location: Nucleus ECO:0000256—RuleBase:RU361211.

(Information from UniProt)

lsy-12 (UniProt:G5EBH5) annotation
Subcellular location: Nucleus ECO:0000256—RuleBase:RU361211.

(Information from UniProt)

lsy-12 (UniProt:K8ERR8) annotation
Subcellular location: Nucleus ECO:0000256—RuleBase:RU361211.

(Information from UniProt)

E3 ubiquitin-protein ligase CBL-B

Best gene implication in ageing for this target family came from gene O46034 annotated in UniProt release 2014_02. Annotation GO 8340 (determination of adult lifespan) was Inferred from Mutant Phenotype

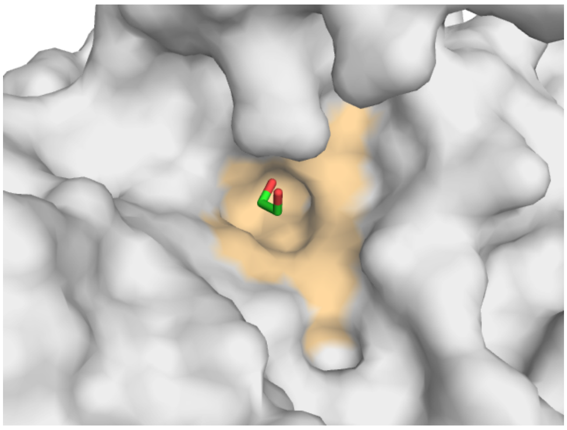

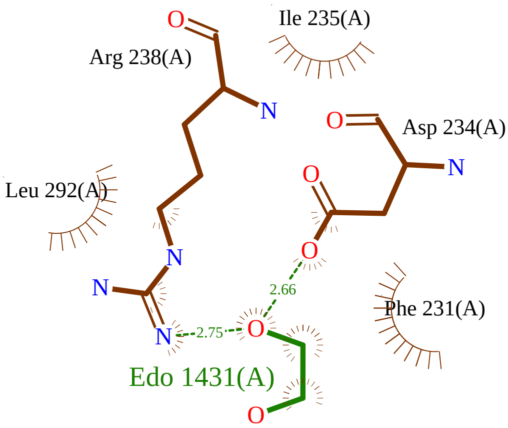

protein	amino acids contacts (binding site)
PDB:3pfv:chainA:Q13191	R F D I R R L
PDB:3zni:chainA:Q13191	R F D I R R L
tr:B5MC15:B5MC15_HUMAN	R F D I R R L
sp:P22681:CBL_HUMAN	R F D I R R L
tr:E7ENW2:E7ENW2_HUMAN	R F D I R R L
sp:Q13191:CBLB_HUMAN	R F D I R R L
tr:D3ZV15:D3ZV15_RAT	R F D I R R L
tr:G3V689:G3V689_RAT	R F D I R R L
sp:P22682:CBL_MOUSE	R F D I R R L
tr:B9EKI5:B9EKI5_MOUSE	R F D I R R L
tr:O46034:O46034_DROME	R F D V R R L
tr:P91683:P91683_DROME	R F D V R R L
tr:Q9VSK2:Q9VSK2_DROME	R F D V R R L
tr:Q6ABG8:Q6ABG8_CAEEL	F F D V R R P
tr:Q95ZR5:Q95ZR5_CAEEL	F F D V R R P
tr:Q19019:Q19019_CAEEL	F F D V R R P

	whole protein		domain-based		contact-based	
protein	ident	simil	ident	simil	ident	simil
PDB:3pfv:chainA:Q13191	1.0	1.0	1.0	1.0	1.0	1.0
PDB:3zni:chainA:Q13191	1.0	1.0	1.0	1.0	1.0	1.0
tr:B5MC15:B5MC15_HUMAN	0.82	0.82	1.0	1.0	1.0	1.0
sp:P22681:CBL_HUMAN	0.47	0.71	0.9	0.96	1.0	1.0
tr:E7ENW2:E7ENW2_HUMAN	0.93	0.93	1.0	1.0	1.0	1.0
sp:Q13191:CBLB_HUMAN	1.0	1.0	1.0	1.0	1.0	1.0
tr:D3ZV15:D3ZV15_RAT	0.47	0.72	0.9	0.96	1.0	1.0
tr:G3V689:G3V689_RAT	0.91	0.94	1.0	1.0	1.0	1.0
sp:P22682:CBL_MOUSE	0.47	0.72	0.9	0.96	1.0	1.0
tr:B9EKI5:B9EKI5_MOUSE	0.91	0.94	1.0	1.0	1.0	1.0
tr:O46034:O46034_DROME	0.3	0.4	0.77	0.93	0.86	0.95
tr:P91683:P91683_DROME	0.3	0.4	0.77	0.93	0.86	0.95
tr:Q9VSK2:Q9VSK2_DROME	0.36	0.64	0.77	0.93	0.86	0.95
tr:Q6ABG8:Q6ABG8_CAEEL	0.24	0.41	0.59	0.86	0.57	0.65
tr:Q95ZR5:Q95ZR5_CAEEL	0.25	0.44	0.59	0.86	0.57	0.65
tr:Q19019:Q19019_CAEEL	0.24	0.43	0.59	0.86	0.57	0.65

Cbl (FBgn0020224) associated phenotypes

decreased cell death, developmental rate defective, dominant, large body, lethal - all die before end of P-stage, male limited, neuroanatomy defective, oxidative stress response defective, partially lethal - majority die, short lived, somatic clone, some die during P-stage, starvation stress response defective

(Information from FlyBase)

sli-1 (WBGene00004829) associated phenotypes

head morphology variant

(Information from WormBase)

ELAV-like protein 1

Best gene implication in ageing for this target family came from gene Q24474 via mapping the annotation from Ensembl FBgn0010263 via mapping the annotation from EntrezGene 33498 via mapping the annotation from GenAgeModels 0877 annotated in GenAge release 17.

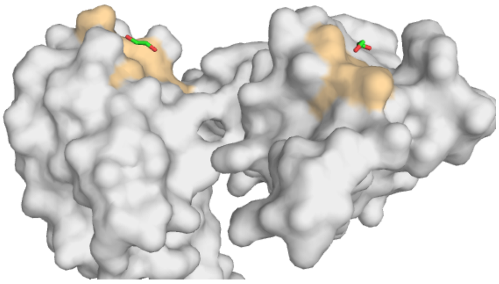

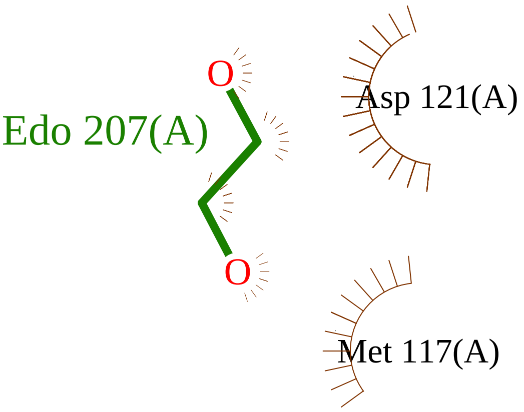

protein	amino acids contacts (binding site)
PDB:4ed5:chainA:Q15717	E I Y T M D
tr:M0QZR9:M0QZR9_HUMAN	E I Y T M D
sp:Q15717:ELAV1_HUMAN	E I Y T M D
tr:B4DVB8:B4DVB8_HUMAN	E I Y T M D
tr:D3ZE54:D3ZE54_RAT	E I Y T M D
tr:B5DF91:B5DF91_RAT	E I Y T M D
tr:Q8BTQ1:Q8BTQ1_MOUSE	E I Y T M D
sp:P70372:ELAV1_MOUSE	E I Y T M D
tr:Q24474:Q24474_DROME	E I I N M D
tr:Q26293:Q26293_DROME	E I I N M D
tr:Q9VQJ0:Q9VQJ0_DROME	E I I N M D
sp:P16914:ELAV_DROME	E V F T M E
tr:A2RVE5:A2RVE5_DROME	E I I N M D
tr:Q20084:Q20084_CAEEL	L V Y S M E

protein	whole protein ident	simil	domain-based ident	simil	contact-based ident	simil
PDB:4ed5:chainA:Q15717	1.0	1.0	1.0	1.0	1.0	1.0
tr:M0QZR9:M0QZR9_HUMAN	0.44	0.46	0.72	0.75	1.0	1.0
sp:Q15717:ELAV1_HUMAN	1.0	1.0	1.0	1.0	1.0	1.0
tr:B4DVB8:B4DVB8_HUMAN	0.92	0.92	1.0	1.0	1.0	1.0
tr:D3ZE54:D3ZE54_RAT	0.67	0.76	0.8	0.89	1.0	1.0
tr:B5DF91:B5DF91_RAT	0.98	1.0	0.99	1.0	1.0	1.0
tr:Q8BTQ1:Q8BTQ1_MOUSE	0.92	0.93	0.96	0.97	1.0	1.0
sp:P70372:ELAV1_MOUSE	0.98	1.0	0.99	1.0	1.0	1.0
tr:Q24474:Q24474_DROME	0.44	0.63	0.71	0.91	0.67	0.85
tr:Q26293:Q26293_DROME	0.44	0.62	0.69	0.89	0.67	0.85
tr:Q9VQJ0:Q9VQJ0_DROME	0.44	0.62	0.69	0.89	0.67	0.85
sp:P16914:ELAV_DROME	0.36	0.57	0.57	0.81	0.5	0.85
tr:A2RVE5:A2RVE5_DROME	0.3	0.43	0.69	0.89	0.67	0.85
tr:Q20084:Q20084_CAEEL	0.35	0.59	0.64	0.89	0.33	0.63

Rbp9 (FBgn0010263) associated phenotypes

conditional, locomotor behavior defective, partially, partially lethal - majority die, short lived

(Information from FlyBase)

elav (FBgn0260400) associated phenotypes
flight defective, flightless, heat sensitive, locomotor behavior defective, neuroanatomy defective, neurophysiology defective, partially lethal - majority die, progressive, sterile, uncoordinated, visual behavior defective

(Information from FlyBase)

elav (UniProt:P16914) annotation
Function: Required for the proper development and maintenance of neurons presumably by affecting RNA metabolism. (PubMed:3144044).

(Information from UniProt)

exc-7 (WBGene00001368) associated phenotypes
aldicarb resistant, excretory canal morphology variant, pale

(Information from WormBase)

Mitogen-activated protein kinase 11

Best gene implication in ageing for this target family came from gene O62618 annotated in UniProt release 2014_02. Annotation GO 8340 (determination of adult lifespan) was Inferred from Mutant Phenotype

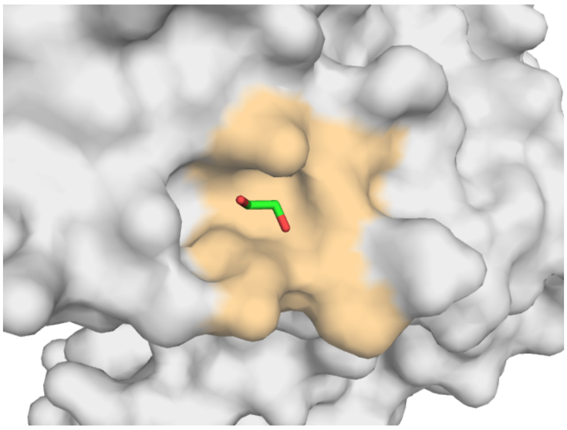

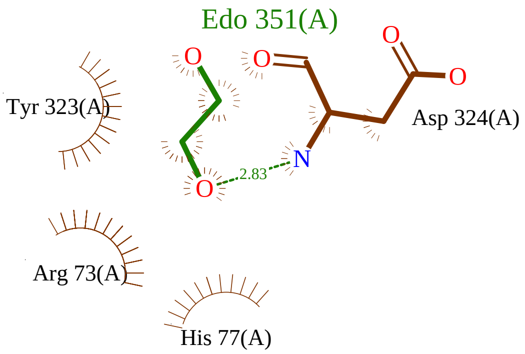

protein	amino acids contacts (binding site)				
PDB:3gp0:chainA:Q15759	R	H	P	Y	D
sp:Q15759:MK11_HUMAN	R	H	P	Y	D
tr:L7RSM2:L7RSM2_HUMAN	R	H	P	Y	D
sp:Q16539:MK14_HUMAN	R	H	P	Y	D
tr:D4A3U7:D4A3U7_RAT	R	H	P	Y	D
tr:G3V617:G3V617_RAT	R	H	P	Y	D
tr:Q56A33:Q56A33_RAT	R	H	P	Y	D
sp:P47811:MK14_MOUSE	R	H	P	Y	D
sp:Q9WUI1:MK11_MOUSE	R	H	P	Y	D
tr:Q5U421:Q5U421_MOUSE	R	H	P	Y	D
sp:O62618:MK14A_DROME	R	H	P	Y	D
tr:E1JIV6:E1JIV6_DROME	R	H	P	Y	D
sp:Q17446:PMK1_CAEEL	R	C	E	M	D
sp:P32485:HOG1_YEAST	K	H	A	F	D

protein	whole protein ident	simil	domain-based ident	simil	contact-based ident	simil
PDB:3gp0:chainA:Q15759	1.0	1.0	1.0	1.0	1.0	1.0
sp:Q15759:MK11_HUMAN	1.0	1.0	1.0	1.0	1.0	1.0
tr:L7RSM2:L7RSM2_HUMAN	0.71	0.92	0.68	0.92	1.0	1.0
sp:Q16539:MK14_HUMAN	0.73	0.92	0.68	0.92	1.0	1.0
tr:D4A3U7:D4A3U7_RAT	0.97	0.99	1.0	1.0	1.0	1.0
tr:G3V617:G3V617_RAT	0.74	0.92	0.68	0.92	1.0	1.0
tr:Q56A33:Q56A33_RAT	0.71	0.92	0.68	0.92	1.0	1.0
sp:P47811:MK14_MOUSE	0.73	0.92	0.68	0.92	1.0	1.0
sp:Q9WUI1:MK11_MOUSE	0.97	0.99	1.0	1.0	1.0	1.0
tr:Q5U421:Q5U421_MOUSE	0.71	0.92	0.68	0.92	1.0	1.0
sp:O62618:MK14A_DROME	0.61	0.85	0.58	0.86	1.0	1.0
tr:E1JIV6:E1JIV6_DROME	0.61	0.85	0.58	0.86	1.0	1.0
sp:Q17446:PMK1_CAEEL	0.59	0.85	0.48	0.83	0.4	0.38
sp:P32485:HOG1_YEAST	0.38	0.68	0.42	0.82	0.4	0.88

Mpk2 (FBgn0015765) associated phenotypes

heat stress response defective, immune response defective, non-enhancer of variegation, non-suppressor of variegation, nutrition conditional, osmotic stress response defective, oxidative stress response defective, partially lethal - majority die, reduced, short lived, starvation stress response defective, stress response defective

(Information from FlyBase)

Mpk2 (UniProt:O62618) annotation

Function: Kinase involved in a signal transduction pathway. May down-regulate insect immunity gene expression after prolonged infection. (PubMed:9417090, PubMed:9584193).
Cofactor:Mg(2+)
Enzyme regulation: Activated by threonine and tyrosine phosphorylation by Mkk3 in response to environmental stress. (PubMed:9417090, PubMed:9584193).
Subcellular location: Nucleus (PubMed:9417090).
Developmental stage: Expressed both maternally and zygotically. Levels are highest at the preblastoderm stage but low levels are present throughout development. (PubMed:9584193).
Domain: The TXY motif contains the threonine and tyrosine residues whose phosphorylation activates the MAP kinases.
Ptm: Dually phosphorylated on Thr-184 and Tyr-186, which activates the enzyme. (PubMed:9417090).

(Information from UniProt)

pmk-1 (WBGene00004055) associated phenotypes

cell stress response variant, drug induced gene expression variant, gene expression level reduced, lethal, life span variant, organism oxidative stress response hypersensitive, pathogen induced gene expression variant, pathogen susceptibility increased, sterile

(Information from WormBase)

pmk-1 (UniProt:Q17446) annotation

Function: Responds to activation by environmental stress and pro- inflammatory cytokines by phosphorylating downstream targets. Functions downstream of the MAPKK sek-1 and the MAPKKK nsy-1 as the MAP kinase required for pathogen resistance. (PubMed:11703092, PubMed:12142542).
Cofactor: Mg(2+) Evidence=(PubMed:11703092);
Enzyme regulation: Activated by phosphorylation on threonine and tyrosine. Inhibited by pyridinyl-imidazole related compounds. (PubMed:11703092).
Domain: The TXY motif contains the threonine and tyrosine residues whose phosphorylation activates the MAP kinases.
Ptm: Dually phosphorylated on Thr-191 and Tyr-193, which activates the enzyme.

(Information from UniProt)

Mitogen-activated protein kinase 14

Best gene implication in ageing for this target family came from gene O62618 annotated in UniProt release 2014_02. Annotation GO 8340 (determination of adult lifespan) was Inferred from Mutant Phenotype

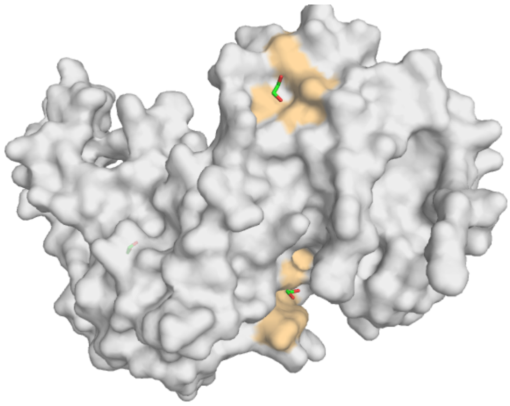

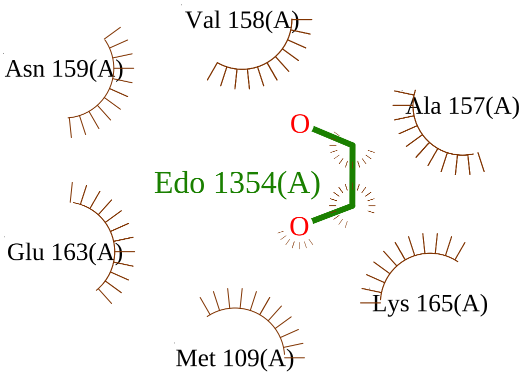

protein	amino acids contacts (binding site)
PDB:3o8p:chainA:Q16539	K Y E L H M A V N E K L G F L R L F L L
PDB:3oc1:chainA:Q16539	K Y E L H M A V N E K L D - L R L F L L
PDB:3zs5:chainA:Q16539	K Y E L H M A V N E K L D F L R L F L L
PDB:4fa2:chainA:Q16539	K Y E L H M A V N E K L D F L R L F L L
sp:Q16539:MK14_HUMAN	K Y E L H M A V N E K L D F L R L F L L
tr:G3V617:G3V617_RAT	K Y E L H M A V N E K L D F L R L F L L
sp:P47811:MK14_MOUSE	K Y E L H M A V N E K L D F L R L F L L
sp:O62618:MK14A_DROME	K Y E L H M A V N E R L D F L E M F L L
tr:E1JIV6:E1JIV6_DROME	K Y E L H M A V N E R L D F L E M F L L
sp:Q8MXI4:PMK2_CAEEL	K Y E L V M A V N E K L D F K S V A T L
sp:P32485:HOG1_YEAST	K Y E L E Q L I N D K C D F I D L F L M

protein	whole protein ident	whole protein simil	domain-based ident	domain-based simil	contact-based ident	contact-based simil
PDB:3o8p:chainA:Q16539	1.0	1.0	1.0	1.0	1.0	1.0
PDB:3oc1:chainA:Q16539	1.0	1.0	1.0	1.0	0.9	0.86
PDB:3zs5:chainA:Q16539	1.0	1.0	1.0	1.0	0.95	0.96
PDB:4fa2:chainA:Q16539	1.0	1.0	1.0	1.0	0.95	0.96
sp:Q16539:MK14_HUMAN	1.0	1.0	1.0	1.0	0.95	0.96
tr:G3V617:G3V617_RAT	0.99	1.0	0.99	1.0	0.95	0.96
sp:P47811:MK14_MOUSE	0.99	1.0	0.99	1.0	0.95	0.96
sp:O62618:MK14A_DROME	0.67	0.87	0.72	0.91	0.8	0.91
tr:E1JIV6:E1JIV6_DROME	0.67	0.87	0.72	0.91	0.8	0.91
sp:Q8MXI4:PMK2_CAEEL	0.49	0.74	0.55	0.81	0.65	0.71
sp:P32485:HOG1_YEAST	0.41	0.68	0.52	0.85	0.5	0.67

Mpk2 (FBgn0015765) associated phenotypes

heat stress response defective, immune response defective, non-enhancer of variegation, non-suppressor of variegation, nutrition conditional, osmotic stress response defective, oxidative stress response defective, partially lethal - majority die, reduced, short lived, starvation stress response defective, stress response defective

(Information from FlyBase)

Mpk2 (UniProt:O62618) annotation

Function: Kinase involved in a signal transduction pathway. May down-regulate insect immunity gene expression after prolonged infection. (PubMed:9417090, PubMed:9584193).
Cofactor:Mg(2+)
Enzyme regulation: Activated by threonine and tyrosine phosphorylation by Mkk3 in response

to environmental stress. (PubMed:9417090, PubMed:9584193).
Subcellular location: Nucleus (PubMed:9417090).
Developmental stage: Expressed both maternally and zygotically. Levels are highest at the preblastoderm stage but low levels are present throughout development. (PubMed:9584193).
Domain: The TXY motif contains the threonine and tyrosine residues whose phosphorylation activates the MAP kinases.
Ptm: Dually phosphorylated on Thr-184 and Tyr-186, which activates the enzyme. (PubMed:9417090).
(Information from UniProt)

pmk-2 (UniProt:Q8MXI4) annotation
Function: Responds to activation by environmental stress and pro- inflammatory cytokines by phosphorylating downstream targets. (PubMed:11703092).
Cofactor: Mg(2+) Evidence=(PubMed:11703092);
Enzyme regulation: Activated by phosphorylation on threonine and tyrosine. Inhibited by pyridinyl-imidazole related compounds. (PubMed:11703092).
Subcellular location: Cytoplasm (PubMed:11703092).
Domain: The TXY motif contains the threonine and tyrosine residues whose phosphorylation activates the MAP kinases.
Ptm: Dually phosphorylated on Thr-222 and Tyr-224, which activates the enzyme.
(Information from UniProt)

NAD-dependent protein deacetylase sirtuin-2

Best gene implication in ageing for this target family came from gene Q9I7I7 annotated in UniProt release 2014_02. Annotation GO 8340 (determination of adult lifespan) was Inferred from Mutant Phenotype

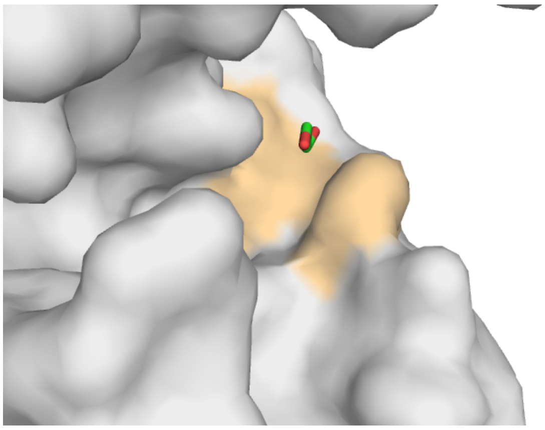

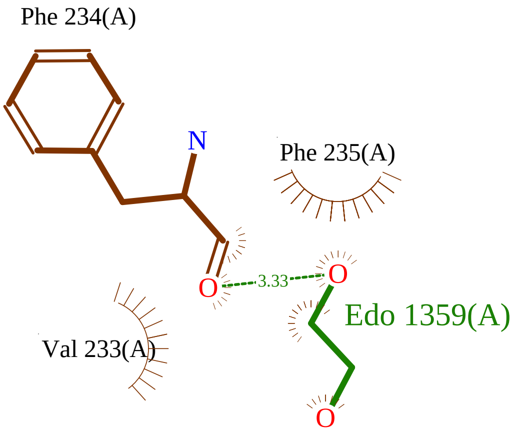

protein	amino acids contacts (binding site)
PDB:3zgo:chainA:Q8IXJ6	V F F
tr:E7EWX6:E7EWX6_HUMAN	V F F
sp:Q8IXJ6:SIR2_HUMAN	V F F
sp:Q5RJQ4:SIR2_RAT	V F F
tr:Q3UJK6:Q3UJK6_MOUSE	V F F
sp:Q8VDQ8:SIR2_MOUSE	V F F
sp:Q9I7I7:SIRT2_DROME	V F F
sp:P53686:HST2_YEAST	V F F

protein	whole protein ident	simil	domain-based ident	simil	contact-based ident	simil
PDB:3zgo:chainA:Q8IXJ6	0.99	1.0	0.99	1.0	1.0	1.0
tr:E7EWX6:E7EWX6_HUMAN	0.61	0.61	0.38	0.38	1.0	1.0
sp:Q8IXJ6:SIR2_HUMAN	1.0	1.0	1.0	1.0	1.0	1.0
sp:Q5RJQ4:SIR2_RAT	0.78	0.87	0.88	0.97	1.0	1.0
tr:Q3UJK6:Q3UJK6_MOUSE	0.79	0.87	0.89	0.97	1.0	1.0
sp:Q8VDQ8:SIR2_MOUSE	0.88	0.97	0.9	0.97	1.0	1.0
sp:Q9I7I7:SIRT2_DROME	0.43	0.72	0.47	0.79	1.0	1.0
sp:P53686:HST2_YEAST	0.29	0.57	0.29	0.57	1.0	1.0

Sirt2 (FBgn0038788) associated phenotypes

heat sensitive, lethal - all die before end of pupal stage, some die during pupal stage

(Information from FlyBase)

Sirt2 (UniProt:Q9I7I7) annotation

Function: NAD-dependent protein deacetylase (By similarity). May be involved in the regulation of life span. (, PubMed:17159295).

Cofactor: Zn(2+)Note=Binds 1 zinc ion per subunit. ;

Disruption phenotype: Causes lethality during development. Induced silencing shortens life span. (PubMed:17159295).

(Information from UniProt)

Heterogeneous nuclear ribonucleoprotein L

Best gene implication in ageing for this target family came from gene Q0E914 annotated in UniProt release 2014_02. Annotation GO 8340 (determination of adult lifespan) was Inferred from Mutant Phenotype

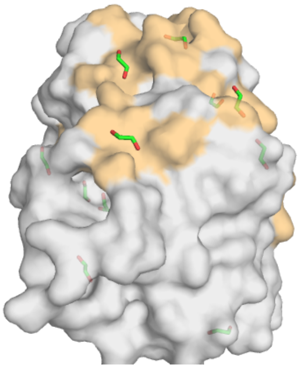

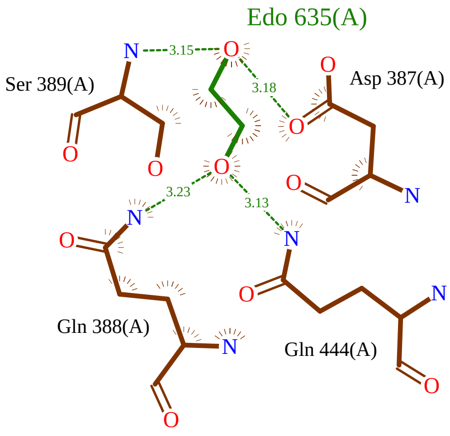

protein	amino acids contacts (binding site)
PDB:3tyt:chainA:Q8R081	P L D Q S K D F N A D G T H N F Q D F S E S N H P G V K R P T F S G W D K S D
tr:Q6NTA2:Q6NTA2_HUMAN	P L D Q S K D F N A D G T H N F Q D F S E S N H P G V K R P S F S G W E K S D
sp:P14866:HNRPL_HUMAN	P L D Q S K D F N A D G T H N F Q D F S E S N H P G V K R P S F S G W E K S D
tr:Q5U1Y5:Q5U1Y5_RAT	P L D Q S K D F N A D G T H N F Q D F S E S N H P G V K R P T F S G W D K S D
tr:F2Z3R2:F2Z3R2_RAT	P L D Q S K D F N A D G T H N F Q D F S E S N H P G V K R P T F S G W D K S D
tr:F1LQ48:F1LQ48_RAT	P L D Q S K D F N A D G T H N F Q D F S E S N H P G V K R P T F S G W D K S D
tr:G3UY38:G3UY38_MOUSE	P L D Q S K D F N A D G T H N F Q D F S E S N H P G V K R P T F S G W D K S D
sp:Q8R081:HNRPL_MOUSE	P L D Q S K D F N A D G T H N F Q D F S E S N H P G V K R P T F S G W D K S D
tr:Q0E914:Q0E914_DROME	A L D H D T D F N G D A Q H I P G E Y T G S N P P D V - P A T F P L F S I S Q
tr:A1ZBN3:A1ZBN3_DROME	A L D H D T D F N G D A Q H I P G E Y T G S N P P D V - P A T F P L F S I S Q
tr:Q8IHH3:Q8IHH3_DROME	A L D H D T D F N G D A Q H I P G E Y T G S N P P D V - P A T F P L F S I S Q
tr:D5AEJ9:D5AEJ9_DROME	A L D H D T D F N G D A Q H I P G E Y T G S N P P D V - P A T F P L F S I S Q
tr:Q7JMZ7:Q7JMZ7_DROME	A L D H D T D F N G D A Q H I P G E Y T G S N P P D V - P A T F P L F S I S Q
tr:Q6NND8:Q6NND8_DROME	A L D H D T D F N G D A Q H I P G E Y T G S N P P D V - P A T F P L F S I S Q
tr:Q95QR5:Q95QR5_CAEEL	C L E H G K D F N G T P D F S A L D Y S S S N F P T E H K P V F P S F E V E I

	whole protein		domain-based		contact-based	
protein	ident	simil	ident	simil	ident	simil
PDB:3tyt:chainA:Q8R081	1.0	1.0	1.0	1.0	1.0	1.0
tr:Q6NTA2:Q6NTA2_HUMAN	0.95	0.96	0.99	1.0	0.95	0.98
sp:P14866:HNRPL_HUMAN	0.98	0.99	0.99	1.0	0.95	0.98
tr:Q5U1Y5:Q5U1Y5_RAT	0.42	0.42	1.0	1.0	1.0	1.0
tr:F2Z3R2:F2Z3R2_RAT	1.0	1.0	1.0	1.0	1.0	1.0
tr:F1LQ48:F1LQ48_RAT	0.94	0.94	1.0	1.0	1.0	1.0
tr:G3UY38:G3UY38_MOUSE	0.78	0.78	1.0	1.0	1.0	1.0
sp:Q8R081:HNRPL_MOUSE	1.0	1.0	1.0	1.0	1.0	1.0
tr:Q0E914:Q0E914_DROME	0.15	0.29	0.39	0.72	0.37	0.58
tr:A1ZBN3:A1ZBN3_DROME	0.17	0.38	0.39	0.72	0.37	0.58
tr:Q8IHH3:Q8IHH3_DROME	0.17	0.38	0.39	0.72	0.37	0.58
tr:D5AEJ9:D5AEJ9_DROME	0.18	0.43	0.39	0.72	0.37	0.58
tr:Q7JMZ7:Q7JMZ7_DROME	0.26	0.48	0.39	0.72	0.37	0.58
tr:Q6NND8:Q6NND8_DROME	0.25	0.48	0.39	0.72	0.37	0.58
tr:Q95QR5:Q95QR5_CAEEL	0.25	0.51	0.3	0.66	0.33	0.58

sm (FBgn0003435) associated phenotypes

feeding behavior defective, long lived, neuroanatomy defective, partially lethal - majority die, short lived, some die during pupal stage

(Information from FlyBase)

Peptidoglycan-recognition protein LF

Best gene implication in ageing for this target family came from gene Q8SXQ7 annotated in UniProt release 2014_02. Annotation GO 8340 (determination of adult lifespan) was Inferred from Mutant Phenotype

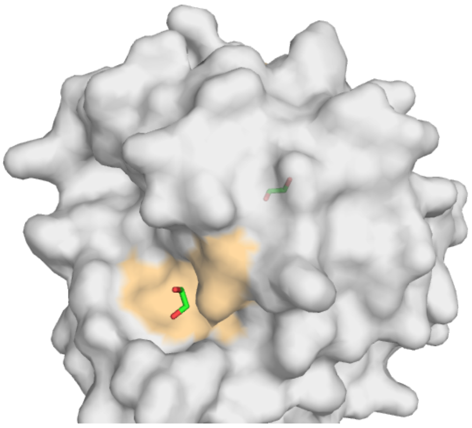

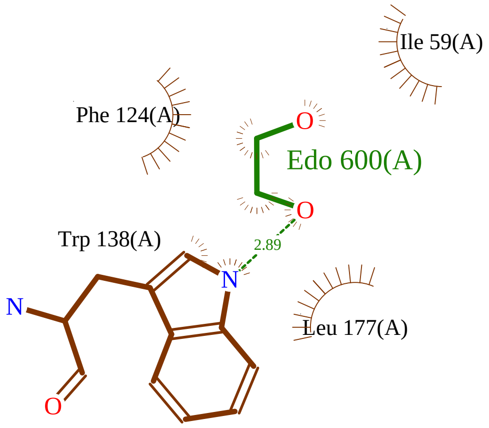

protein	amino acids contacts (binding site)
PDB:2xz4:chainA:Q8SXQ7	I H H T A F F Y F W V L H S
sp:Q96PD5:PGRP2_HUMAN	I H H T Y Y Q Y F W T T H C
tr:M0R485:M0R485_RAT	I H H T Y F R Y L - - - - -
tr:Q54A77:Q54A77_MOUSE	I H H T Y F R Y F W T A H C
sp:Q8VCS0:PGRP2_MOUSE	I H H T Y F R Y F W T A H C
sp:Q8SXQ7:PGPLF_DROME	I H H T A F F Y F W V L H S

	whole protein		domain-based		contact-based	
protein	ident	simil	ident	simil	ident	simil
PDB:2xz4:chainA:Q8SXQ7	1.0	1.0	1.0	1.0	1.0	1.0
sp:Q96PD5:PGRP2_HUMAN	0.1	0.24	0.12	0.27	0.57	0.65
tr:M0R485:M0R485_RAT	0.05	0.13	0.06	0.15	0.43	0.15
tr:Q54A77:Q54A77_MOUSE	0.1	0.22	0.13	0.29	0.64	0.68
sp:Q8VCS0:PGRP2_MOUSE	0.1	0.22	0.13	0.29	0.64	0.68
sp:Q8SXQ7:PGPLF_DROME	1.0	1.0	1.0	1.0	1.0	1.0

PGRP-LF (FBgn0035977) associated phenotypes

immune response defective, long lived, partially lethal - majority die

(Information from FlyBase)

PGRP-LF (UniProt:Q8SXQ7) annotation

Function: Peptidoglycan-recognition protein probably involved in innate immnunity by binding to peptidoglycans (PGN) of bacteria and activating the immune response.
Subcellular location: Membrane ; Multi-pass membrane protein
Induction: Regulated by the imd/Relish pathway. (PubMed:12032070).

(Information from UniProt)

CREB-binding protein

Best gene implication in ageing for this target family came from gene Q92793 via mapping the annotation from Ensembl ENSG00000005339 via mapping the annotation from EntrezGene 1387 via mapping the annotation from GenAgeHuman 0064 annotated in GenAge release 17.

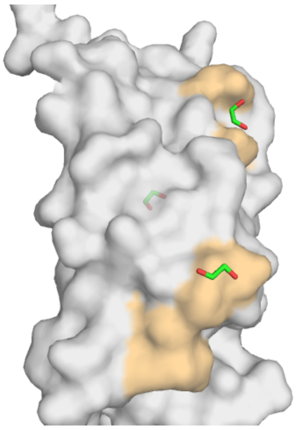

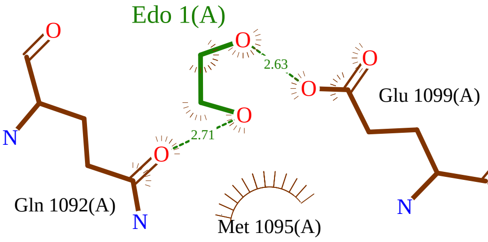

protein	amino acids contacts (binding site)
PDB:3dwy:chainA:Q92793	P R Q M E R D P L R P V D P Q G I P F K L D G Q Y Q W R S R Y F
PDB:3p1f:chainA:Q92793	P R Q M E R D P L R P V D P Q G I P F K L D G Q Y Q W R S R Y F
PDB:3svh:chainA:Q92793	P R Q M E R D P L R P V D P Q G I P F K L D G Q Y Q W R S R Y F
PDB:4a9k:chainA:Q92793	P R Q M E R D P L R P V D P Q G I P F K L D G Q Y Q W R S R Y F
PDB:4nyw:chainA:Q92793	P R Q M E R D P L R P V D P Q G I P F K L D G Q Y Q W R S R Y F
PDB:4ouf:chainA:Q92793	P R Q M E R D P L R P V D P Q G I P F K L D G Q Y Q W R S R Y F
sp:Q92793:CBP_HUMAN	P R Q M E R D P L R P V D P Q G I P F K L D G Q Y Q W R S R Y F
tr:F1M9G7:F1M9G7_RAT	P R Q M E R D P L R P V D P Q G I P F K L D G Q Y Q W R S R Y F
tr:Q8QZV8:Q8QZV8_MOUSE	P R Q M E R D P L R P V D P Q G I P F K L D G Q Y Q W R S R Y F
tr:F8VPR5:F8VPR5_MOUSE	P R Q M E R D P L R P V D P Q G I P F K L D G Q Y Q W R S R Y F
tr:Q8T3Z6:Q8T3Z6_DROME	R L E K N R N R Y R P V D S V G V P H R L H K Y Y E L L S P Y A
tr:Q9VCG6:Q9VCG6_DROME	R L E K N R N R Y R P V D S V G V P H R L H K Y Y E L L S P Y A
tr:O17681:O17681_CAEEL	- -
tr:Q9TZB8:Q9TZB8_CAEEL	- -
sp:P35817:BDF1_YEAST	S Q Q K S K A S Y L P V D P V N L P F K L N W Q Y D Y P T I N M

	whole protein		domain-based		contact-based	
protein	ident	simil	ident	simil	ident	simil
PDB:3dwy:chainA:Q92793	1.0	1.0	1.0	1.0	1.0	1.0
PDB:3p1f:chainA:Q92793	0.98	0.99	0.98	0.99	1.0	1.0
PDB:3svh:chainA:Q92793	1.0	1.0	1.0	1.0	1.0	1.0
PDB:4a9k:chainA:Q92793	1.0	1.0	1.0	1.0	1.0	1.0
PDB:4nyw:chainA:Q92793	1.0	1.0	1.0	1.0	1.0	1.0
PDB:4ouf:chainA:Q92793	1.0	1.0	1.0	1.0	1.0	1.0
sp:Q92793:CBP_HUMAN	1.0	1.0	1.0	1.0	1.0	1.0
tr:F1M9G7:F1M9G7_RAT	0.96	0.99	0.98	0.99	1.0	1.0
tr:Q8QZV8:Q8QZV8_MOUSE	0.95	0.98	0.98	1.0	1.0	1.0
tr:F8VPR5:F8VPR5_MOUSE	0.96	0.99	0.98	1.0	1.0	1.0
tr:Q8T3Z6:Q8T3Z6_DROME	0.02	0.07	0.3	0.65	0.34	0.58
tr:Q9VCG6:Q9VCG6_DROME	0.03	0.13	0.3	0.65	0.34	0.58
tr:O17681:O17681_CAEEL	0.01	0.04	0.0	0.0	0.0	0.0
tr:Q9TZB8:Q9TZB8_CAEEL	0.03	0.07	0.0	0.0	0.0	0.0
sp:P35817:BDF1_YEAST	0.04	0.17	0.29	0.71	0.34	0.58

tbrd-1 (FBgn0039124) associated phenotypes

male fertile

(Information from FlyBase)

Protein deglycase DJ-1

Best gene implication in ageing for this target family came from gene A1Z9J4 via mapping the annotation from Ensembl FBgn0033885 via mapping the annotation from EntrezGene 36543 via mapping the annotation from GenAgeModels 0178 annotated in GenAge release 17.

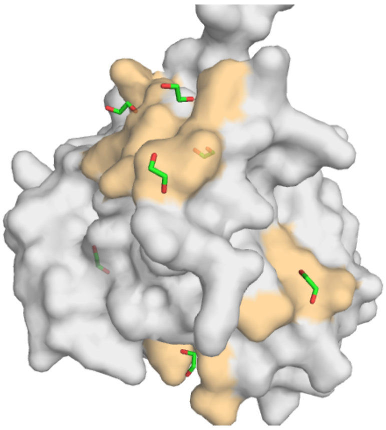

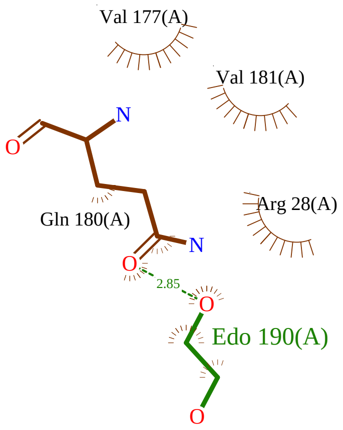

protein	amino acids contacts (binding site)
PDB:3b36:chainA:Q99497	L A K E R R G I K G L A V R G P Y V N Q N I E Q C A F K H L A K S N R V E K D
PDB:3b38:chainA:Q99497	L A K E R R G I K G L A V R G P Y V N Q N I E Q C A F K H L A K S N R V E K D
PDB:3b3a:chainA:Q99497	L A K E R R G I K G L A V R G P Y V N Q N I E Q C A F K H L A K S N E V E K D
PDB:4mtc:chainA:Q99497	L A K E R R G I K G L A V R G P Y V N Q N I E Q C A F K H L A K S N R V E K D
PDB:4n0m:chainA:Q99497	L A K E R R G I K G L A V R G P Y V N Q N I E Q C A F K H L A K S N R V E K D
PDB:4n12:chainA:Q99497	L A K E R R G I K G L A V R G P Y V N Q N I E Q X A F K H L A K S N R V E K D
PDB:4p2g:chainA:Q99497	L A K E R R G I K G L A V R G P Y V N Q N I E Q X A F K H L A K S N R V E K D
sp:Q99497:PARK7_HUMAN	L A K E R R G I K G L A V R G P Y V N Q N I E Q C A F K H L A K S N R V E K D
sp:O88767:PARK7_RAT	L A K E R R G I K G L A V R G P Y V N Q N I E Q C A F K H L A K S S R V E K D
sp:Q99LX0:PARK7_MOUSE	L A K E R R G I K G L A V R G P Y V N Q N I E Q C A F K H L A K S S R V E K D
tr:A1Z9J4:A1Z9J4_DROME	L A P E R R K I L G L H V R G D Y V L K A V C Q C A K S H D M Q I D K V V Q D
tr:P90994:P90994_CAEEL	L A E E A R E I R G L D V R - K F V Q N T V S Q C A K L H S V K S D R V V V S

	whole protein		domain-based		contact-based	
protein	ident	simil	ident	simil	ident	simil
PDB:3b36:chainA:Q99497	0.99	1.0	0.99	1.0	1.0	1.0
PDB:3b38:chainA:Q99497	0.98	0.98	0.99	1.0	1.0	1.0
PDB:3b3a:chainA:Q99497	0.99	1.0	0.99	1.0	0.98	0.99
PDB:4mtc:chainA:Q99497	0.99	1.0	0.99	1.0	1.0	1.0
PDB:4n0m:chainA:Q99497	0.99	1.0	0.99	1.0	1.0	1.0
PDB:4n12:chainA:Q99497	0.99	0.99	0.99	0.99	0.98	0.97
PDB:4p2g:chainA:Q99497	0.99	1.0	0.99	1.0	0.98	0.97
sp:Q99497:PARK7_HUMAN	1.0	1.0	1.0	1.0	1.0	1.0
sp:O88767:PARK7_RAT	0.92	0.98	0.91	0.98	0.94	0.97
sp:Q99LX0:PARK7_MOUSE	0.92	0.98	0.91	0.98	0.94	0.97
tr:A1Z9J4:A1Z9J4_DROME	0.46	0.69	0.54	0.81	0.49	0.56
tr:P90994:P90994_CAEEL	0.52	0.82	0.52	0.82	0.47	0.6

DJ-1alpha (FBgn0033885) associated phenotypes

chemical resistant, chemical sensitive, increased cell death, locomotor behavior defective, short lived, stress response defective

(Information from FlyBase)

djr-1.1 (WBGene00015184) associated phenotypes

solitary feeding increased

(Information from WormBase)

djr-1.1 (UniProt:P90994) annotation

Function: Catalyzes the conversion of methylglyoxal (MG) or glyoxal (GO) to D-lactate or glycolic acid respectively in a single glutathione (GSH)-independent step. May play a role in detoxifying endogenously produced glyoxals. Involved in protection against glyoxal-induced cell death. (PubMed:22523093).

Biophysicochemical properties: Kinetic parameters: KM=0.3 mM for methylglyoxal (PubMed:22523093); KM=3.61 mM for glyoxal (PubMed:22523093); Note=kcat is 13.8 min(-1) with methylglyoxal as substrate and 356.4 min(-1) with glyoxal as substrate. (PubMed:22523093);
Subcellular location: Cytoplasm (PubMed:22523093). Nucleus (PubMed:22523093). Note=Ubiquitously localized throughout the whole cells with higher expression in the nucleus. (PubMed:22523093).
Tissue specificity: Expressed exclusively in the intestine. (PubMed:22523093).

(Information from UniProt)

NAD-dependent protein deacetylase sirtuin-3, mitochondrial

Best gene implication in ageing for this target family came from gene Q9I7I7 annotated in UniProt release 2014_02. Annotation GO 8340 (determination of adult lifespan) was Inferred from Mutant Phenotype

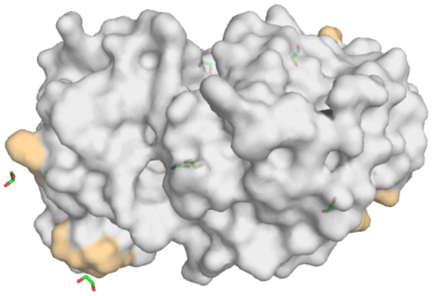

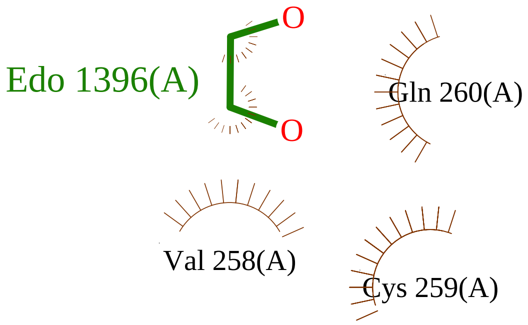

protein	amino acids contacts (binding site)
PDB:4bve:chainA:Q9NTG7	R A Q R S H Y P N Y K G L L N I G K E G T F A V C Q G R R Q
PDB:4bvf:chainA:Q9NTG7	R A Q R S H Y P N Y K G L L N I G K E G T F A V C Q G R R Q
PDB:4bvg:chainA:Q9NTG7	R A Q R S H Y P N Y K G L L N I G K E G T F A V C Q G R R Q
tr:E9PK80:E9PK80_HUMAN	R A Q R S H Y P N Y K G L L N I G K E G T F A V C Q G R R Q
tr:E9PM75:E9PM75_HUMAN	R A Q R S H Y P N Y K G L L N I G K E G T F A V C Q G R R Q
sp:Q9NTG7:SIR3_HUMAN	R A Q R S H Y P N Y K G L L N I G K E G T F A V C Q G R R Q
tr:C6ZII9:C6ZII9_RAT	R A S R S H Y P H Y R E L L N I G K E G S F V V C R G R Q Q
sp:Q8R104:SIR3_MOUSE	R A S R S H Y P H Y R E L L N I G K E G T F V V C R G W L Q
sp:Q9I7I7:SIRT2_DROME	H G R K A K Y P S F I G L L N I T K E G S F H K C R M K Q T
sp:P53686:HST2_YEAST	N P A K C S Y P N F R D V L N I T L E G S F A G C G P K E T

	whole protein		domain-based		contact-based	
protein	ident	simil	ident	simil	ident	simil
PDB:4bve:chainA:Q9NTG7	1.0	1.0	1.0	1.0	1.0	1.0
PDB:4bvf:chainA:Q9NTG7	1.0	1.0	1.0	1.0	1.0	1.0
PDB:4bvg:chainA:Q9NTG7	1.0	1.0	1.0	1.0	1.0	1.0
tr:E9PK80:E9PK80_HUMAN	0.8	0.8	0.98	0.98	1.0	1.0
tr:E9PM75:E9PM75_HUMAN	0.86	0.86	0.8	0.8	1.0	1.0
sp:Q9NTG7:SIR3_HUMAN	1.0	1.0	1.0	1.0	1.0	1.0
tr:C6ZII9:C6ZII9_RAT	0.63	0.74	0.86	0.96	0.73	0.85
sp:Q8R104:SIR3_MOUSE	0.62	0.72	0.85	0.96	0.73	0.82
sp:Q9I7I7:SIRT2_DROME	0.34	0.55	0.46	0.7	0.4	0.7
sp:P53686:HST2_YEAST	0.26	0.49	0.35	0.64	0.37	0.66

Sirt2 (FBgn0038788) associated phenotypes
heat sensitive, lethal - all die before end of pupal stage, some die during pupal stage

(Information from FlyBase)

Sirt2 (UniProt:Q9I7I7) annotation

Function: NAD-dependent protein deacetylase (By similarity). May be involved in the regulation of life span. (, PubMed:17159295).

Cofactor: Zn(2+)Note=Binds 1 zinc ion per subunit. ;

Disruption phenotype: Causes lethality during development. Induced silencing shortens life span. (PubMed:17159295).

(Information from UniProt)
